# Impairment of Multiple Mitochondrial Energy Metabolism Pathways in the Heart of Chagas Disease Cardiomyopathy Patients

**DOI:** 10.3389/fimmu.2021.755782

**Published:** 2021-11-12

**Authors:** Priscila Camillo Teixeira, Axel Ducret, Hanno Langen, Everson Nogoceke, Ronaldo Honorato Barros Santos, João Paulo Silva Nunes, Luiz Benvenuti, Debora Levy, Sergio Paulo Bydlowski, Edimar Alcides Bocchi, Andréia Kuramoto Takara, Alfredo Inácio Fiorelli, Noedir Antonio Stolf, Pablo Pomeranzeff, Christophe Chevillard, Jorge Kalil, Edecio Cunha-Neto

**Affiliations:** ^1^ Laboratory of Immunology, Heart Institute (Incor) Hospital das Clínicas da Faculdade de Medicina da Universidade de São Paulo, São Paulo, Brazil; ^2^ Roche Pharma Research and Early Development, Roche Innovation Center Basel, F. Hoffmann-La Roche, Basel, Switzerland; ^3^ Division of Surgery, Heart Institute, School of Medicine, University of São Paulo, São Paulo, Brazil; ^4^ INSERM, UMR_1090, Aix Marseille Université, TAGC Theories and Approaches of Genomic Complexity, Institut MarMaRa, Marseille, France; ^5^ Division of Clinical Immunology and Allergy, Faculdade de Medicina da Universidade de São Paulo, São Paulo, Brazil; ^6^ Instituto Nacional de Ciência e Tecnologia, INCT, iii- Institute for Investigation in Immunology, São Paulo, Brazil; ^7^ Anatomical Pathology Division, Heart Institute (Incor) Hospital das Clínicas da Faculdade de Medicina da Universidade de São Paulo, São Paulo, Brazil; ^8^ Heart Failure Team, Heart Institute (Incor) Hospital das Clínicas da Faculdade de Medicina da Universidade de São Paulo, São Paulo, Brazil

**Keywords:** chronic Chagas disease cardiomyopathy, ischemic cardiomyopathy, idiopathic dilated cardiomyopathy, proteomics, two-dimensional electrophoresis with fluorescent labeling, mitochondria, energy metabolism, interferon-gamma

## Abstract

Chagas disease cardiomyopathy (CCC) is an inflammatory dilated cardiomyopathy occurring in 30% of the 6 million infected with the protozoan *Trypanosoma cruzi* in Latin America. Survival is significantly lower in CCC than ischemic (IC) and idiopathic dilated cardiomyopathy (DCM). Previous studies disclosed a selective decrease in mitochondrial ATP synthase alpha expression and creatine kinase activity in CCC myocardium as compared to IDC and IC, as well as decreased *in vivo* myocardial ATP production. Aiming to identify additional constraints in energy metabolism specific to CCC, we performed a proteomic study in myocardial tissue samples from CCC, IC and DCM obtained at transplantation, in comparison with control myocardial tissue samples from organ donors. Left ventricle free wall myocardial samples were subject to two-dimensional electrophoresis with fluorescent labeling (2D-DIGE) and protein identification by mass spectrometry. We found altered expression of proteins related to mitochondrial energy metabolism, cardiac remodeling, and oxidative stress in the 3 patient groups. Pathways analysis of proteins differentially expressed in CCC disclosed mitochondrial dysfunction, fatty acid metabolism and transmembrane potential of mitochondria. CCC patients’ myocardium displayed reduced expression of 22 mitochondrial proteins belonging to energy metabolism pathways, as compared to 17 in DCM and 3 in IC. Significantly, 6 beta-oxidation enzymes were reduced in CCC, while only 2 of them were down-regulated in DCM and 1 in IC. We also observed that the cytokine IFN-gamma, previously described with increased levels in CCC, reduces mitochondrial membrane potential in cardiomyocytes. Results suggest a major reduction of mitochondrial energy metabolism and mitochondrial dysfunction in CCC myocardium which may be in part linked to IFN-gamma. This may partially explain the worse prognosis of CCC as compared to DCM or IC.

## Introduction

Heart failure can be seen as a progressive disorder resulting from loss of cardiomyocyte function and contractility decline in the ability of the heart, due to molecular and structural modifications, collectively called cardiac remodeling (1). Chagas’ disease is a neglected disease and a significant cause of morbidity and mortality in Central and South America, affecting about 6 million people (2). The disease is caused by infection with the intracellular protozoan parasite *Trypanosoma cruzi (T. cruzi)*. About 30% of infected patients develop chronic Chagas’ disease cardiomyopathy (CCC), an inflammatory dilated cardiomyopathy that occurs decades after the initial infection, while 60% remain asymptomatic (ASY) and 10% develop gastrointestinal motility disorders. Chagas disease is the most common cause of non-ischemic cardiomyopathy in Latin America, where 6 million people are infected, causing approximately 10,000 deaths/year, mainly due cardiac compromise (2). Clinical progression, length of survival and overall prognosis are significantly worse in CCC patients compared with patients with dilated cardiomyopathy of non-inflammatory etiology (3–6). Due to migration to non-endemic countries, Chagas disease cardiomyopathy is now a global health problem (7). Refractory heart failure due to CCC is one of the main indications for heart transplantation in endemic countries. A recent report disclosed 25 cases of heart transplantation due to CCC in the USA, indicating the presence of patients with severe complications from Chagas disease (8). As the currently licensed anti-*T. cruzi* drugs are not effective in preventing the progression of heart lesions of CCC (9), treatment is only supportive. The absence of alternative specific treatment for CCC is a consequence of limited knowledge about the pathogenesis.

The pathogenesis of CCC is incompletely understood, and multiple mechanisms have been proposed, but myocarditis seems to play an important role [Reviewed in (2, 10)]. After acute infection by *T. cruzi*, parasitism is partially controlled by the immune response. Low-grade parasite persistence fuels the systemic production of inflammatory cytokines like IFN-gamma and TNF-alpha by T cells, which is more intense in CCC than ASY patients along the chronic phase of infection (11–13). CCC is characterized by a myocarditis rich in monocytes and IFN-gamma-producing T cells attracted to the heart by locally produced chemokines such as CCL5 and CXCL9 (14), with cardiomyocyte damage, fibrosis and hypertrophy; *T. cruzi* parasites are very scarce. Indeed, IFN-gamma is the most highly expressed cytokine in CCC myocardium (11, 12, 14–19) and a significant number of genes expressed in CCC myocardium is modulated by IFN-gamma (15, 20). T cells infiltrating the heart of CCC patients recognize both *T. cruzi* (21, 22) and pathogen-cross reactive autoimmune targets in the heart, like cardiac myosin and *T cruzi* antigen B13 (21). This aggressive myocarditis is without parallel in other etiologies of dilated cardiomyopathy, which may suggest the pathogenesis of cardiomyopathy due to Chagas disease may be distinct from non-inflammatory cardiomyopathy. In addition, the myocardium from CCC patients with ventricular dysfunction displays selectively decreased levels and activity of several mitochondrial energy metabolism enzymes, including mitochondrial ATP synthase alpha expression and creatine kinase activity, as compared with idiopathic dilated or ischemic cardiomyopathy (DCM and IC respectively) (23). Patients with CCC also displayed decreased *in vivo* myocardial ATP flux as determined by ^31^P-NMR spectroscopy (24). These mitochondrial changes are thought to contribute to the worse prognosis of CCC as compared to other cardiomyopathies (25).

Systems biology approaches have been used to understand the pathogenesis of CCC, including gene expression and miRNA expression profiling (15, 20) and DNA methylation studies (26). Collectively, omics studies in Chagas disease indicate induction of genes related to cardiac hypertrophy, fibrosis, mitochondria/oxidative stress and arrhythmia, in common with other cardiomyopathies, but with a prominent set of differentially expressed inflammatory and IFN-gamma-dependent genes. Proteomic analysis has been widely applied to study cardiovascular disease also pointing toward the embryonic/hypertrophic phenotype and fibrosis (27–30). Studies are also needed to map the main proteins involved in the development of cardiovascular diseases. Given the striking inflammatory pattern and increased number of IFN-gamma-inducible gene among differentially expressed genes in CCC myocardium, we could hypothesize that proteomic analysis may shed light on the mechanisms of pathogenesis in CCC and its worse prognosis as compared to other cardiomyopathies. Given the ability of IFN-gamma to directly induce genes expression on cardiomyocytes (15) we assessed its ability to cause functional impairment in cardiomyocytes.

## Material and Methods

### Patients and Samples of Human Myocardium

Myocardial samples were obtained from left ventricular-free wall heart tissue from end-stage heart failure patients at the moment of heart transplantation. Samples from 4 CCC (serological diagnosis, positive epidemiology for Chagas disease), 4 idiopathic dilated cardiomyopathy (DCM; dilated cardiomyopathy in the absence of ischemic disease, negative epidemiology and serology for Chagas disease) and 4 coronary angiography-proven dilated cardiomyopathy (IC) patients were collected for proteomic analysis (**Table 1**). Left ventricular free wall samples were also obtained from healthy hearts from organ donors, which were not designated for transplantation for technical reasons (control group). Additional myocardium samples from patients with CCC, DCM and IC and organ donors were used for confirmatory experiments (real-time PCR and immunoblotting) (**Supplemental Table S1**). Samples were cleared from pericardium and fat tissue, quickly frozen in liquid nitrogen and stored at -70°C. Protein homogenates were obtained using lysing solution (1:10w/v) containing 7mol/L urea, 10mmol/L Tris, 5mmol/L magnesium acetate and 4% CHAPS, pH 8.0, with mechanical homogenization (PowerGen, Fisher Scientific). The homogenate was then sonicated for three cycles of 10 seconds each to 10 Watts (60 Dismembrator Sonic, Fisher Scientific), centrifuged at 12,000g for 30 minutes. Supernatants were collected and stored at -70°C. Protein quantification was performed with the Bradford method (BioRad).

**Table 1:** Baseline characteristics of patients included in the differential proteomic analysis.

Etiol^*^	Patient	Gender	Age	EF (%)^†^	RVDD (cm)^‡^	Fibrosis^§^	Myocarditis^||^
CCC	#1	M	50	11	82	2+	2/3+
CCC	#2	M	57	29	71	1+	2/3+
CCC	#3	M	58	29	64	2+	2+
CCC	#4	M	59	17	64	2+	3+
IC	#1	M	49	25	76	1+	0
IC	#2	M	61	33	79	3+	0
IC	#3	M	52	20	62	3+	0
IC	#4	M	55	16	83	2+	0
DCM	#1	M	53	19	77	1/2+	0
DCM	#2	M	55	25	51	3+	0
DCM	#3	M	56	16	99	2+	0
DCM	#4	M	61	27	76	3+	0
N	#1	M	17	na	na	na	na
N	#2	M	22	na	na	na	na
N	#3	M	28	na	na	na	na
N	#4	M	40	na	na	na	na

*Etiol., Etiology; ^†^EF, Ejection Fraction (reference value, ≥55%); ^‡^ LVDD, Left Ventricular Diastolic Diameter (reference value, 39-53mm); ^§^ and ^||^ as rated by histopatology (0, absent; 1 +, mild; 2 +, moderate; 3 +, intense); N, individuals without cardiomyopathies; CCC, chronic Chagas disease cardiomyopathy; DCM, idiopathic dilated cardiomyopathy; IC, ischemic cardiomyopathy; M, Male; na, not applicable or not available.

### Two-Dimensional Electrophoresis (2D-DIGE)

For the separation of the myocardium proteins we used two-dimensional electrophoresis with a multiplexing fluorescent labeling approach (**Supplemental Figure S1**), that enables detection of small differences in protein levels as well as inclusion of an internal standard. The standard is a pool of all the samples within the experiment and therefore contains all proteins relevant for the experiment. By using an internal standard, gel-to-gel variation can be eliminated, quantification is accurate and system variation can be separated from biological variation. The first step was the individual labelling of each sample; 50ug protein of each sample (concentration 5μg/μL and pH 8.5) were labeled with 400pmol of one of the fluorophores (CY2, 3, or 5 - GE Healthcare) reconstituted with dimethylformamide. The reaction occurred on ice for 30 minutes, and stopped by adding 1μL of lysine 10mM. The individual samples were labeled with Cy3 or Cy5 fluorophores, and the standard sample (“pool”) was labeled with the fluorophore Cy2. For each gel, two individual samples, one labeled with Cy3 and one labeled with Cy5, plus the internal standard (“pool”) labeled with Cy2 were combined and applied to the two-dimensional electrophoresis. This procedure was performed for the preparation of the analytical gels. For the identification of proteins by mass spectrometry, a “preparative gel” was performed using the pool sample (500μg total protein: 450µg of unlabeled protein and 50µg of labeled protein with Cy2, the internal standard). The first dimension was performed in isoelectric focusing system “Ettan IPGphor” (GE Healthcare). The strips of immobilized pH gradient at pH 3 to 11 nonlinear gradient of 24cm (IPG strip) were rehydrated for at least 12 hours, with rehydration solution “DeStreak Rehydratation Solution” containing 0.5% “IPG buffer 3-11NL” (GE Healthcare). The electrodes were placed at the ends of the strips, and were then taken to the platform of “Ettan IPGphor (GE Healthcare) for isoelectric focusing so that the end of the focus was accumulated 64kV. After isoelectrofocusing, the strips were subjected to two rounds of equilibrium for the preparation of proteins for the second dimension. The first step, the reduction of proteins, was performed with the equilibrium solution (50mM tris-HCl, 6M urea, 30% glycerol, 2% SDS and 0.002% bromophenol blue, pH = 8.8) plus 10mg/mL of DTT (dithiothreitol) for 15 minutes. Second, alkylation of proteins was performed with the equilibrium solution plus 25 mg/ml iodoacetamide for 15 minutes. The second dimension was performed in the electrophoresis vertical “Ettan Daltsix” (6 gels of 25.5 x 20.5cm) (GE Healthcare). For gels with samples labeled with the fluorophore, we used glass plates with low fluorescence. The polyacrylamide gels were 12.5%, over which the strips were placed. The set was sealed with 0.5% agarose in electrophoresis buffer. The electrophoresis was performed in buffer containing 25mM Tris-HCl, 192mM glycine, and 0.1% SDS, pH 8.3 with a power of 2.5W/gel for 30 minutes and 100W total by the end of the race. To maintain the temperature to 20°C in the tank “DaltSix” was used a cooler “MultiTemp III (GE Healthcare) at 10°C. After the second dimension, gels were fixed in a solution containing 40% methanol and 10% acetic acid. Only the “preparative gel” was stained in a solution containing 8% ammonium sulfate, 0.8% phosphoric acid, 0.08% Coomassie Blue G-250 and 20% methanol, and then bleached in water. The gels were kept in a solution containing 15% ethanol. The “preparative gel” stained with Colloidal Coomassie Blue were scanned by the device “ImageScanner” (GE Healthcare) using green filter, transparent and 300dpi resolution. The “analytical gels” containing samples labeled with fluorophores were scanned by the device “Typhoon 9410 Variable Mode Imager” (GE Healthcare), using the following parameters: Cy2, 488nm excitation and 520 nm-BP 40nm emission filter; Cy3, 532nm excitation and 580nm - BP 40nm emission filter; Cy5, 633nm excitation and 670nm - BP 40nm emission; with resolution 100micra and the sensitivity ranged from 450 to 550PTM.

### Statistical Analysis of Differential Protein Expression Using the DIGE

The analysis of differential protein expression using the DIGE technique was performed using the DeCyder Differential Analysis version 6.5 software (GE Healthcare). The volume of each spot was normalized in relation to the total volume of spots selected, and the gels were normalized together using the image of the pool of samples labeled with Cy2. The proteomic profiles of each disease group were compared with healthy donor subjects (control group). For each experimental group, spots present in at least 80% of samples were considered. One of the gels was chosen as a reference (“master” gel, Cy2 labeling in the preparative gel) for the spots matching between the gels. Statistically significant differences of 2D-DIGE data were computed by analysis of variance (ANOVA) and Student t-tests. False discovery rate was applied as a multiple test correction in order to keep the overall error rate as low as possible, according to software manual and literature (31). Power analysis was conducted on statistically changed spots, and only spots that reached a sensitivity threshold > 0.8 were considered as differentially expressed. We considered a protein to be differentially expressed if one or more of the associated spots was differentially expressed (p<0.01). The Ingenuity Pathways Analysis (IPA^©^, Qiagen, Redwood City, USA) software was used to analyze the differentially expressed protein profile. Qiagen Ingenuity Pathway analysis software provides a P value representing Fisher’s exact test representation of proteins in each pathway that are present or not in each pathway. The more proteins in the pathway, the lower the P value. No FDR calculation was applied in this analysis.

### Automatic in Gel Protein Digestion

After detection of spots, these were picked from the “preparative gel” (previously stained with Colloidal Coomassie Blue) and processed by automated system “Ettan Spot Handling Workstation” (GE Healthcare). In this system the spots were removed from the gel and submitted to tryptic “in gel” digestion. The tryptic peptide fragments were extracted from the gel spots for further analysis by mass spectrometry. In summary, gel spots were treated with solutions containing ammonium bicarbonate and acetonitrile. Each spot gel was dried and trypsin (1.6mg/ml in 20mM ammonium bicarbonate) was added. Digestion was performed at 37°C for 6 hours. The product of tryptic digestion was extracted from the spot gel with a solution containing 50% acetonitrile and 0.45% trifluoroacetic acid (TFA). The product was then dried and resuspended in 5μL of 50% acetonitrile and 0.5% TFA prior to mass spectrometry analysis.

### Analysis by Mass Spectrometry and Protein Identification

An aliquot (1μL) of the tryptic digest was placed onto a MALDI target slide and 1.5μL matrix solution with standards [5 mg/ml α-Cyano-4-hydroxycinnamic acid in 50% Acetonitrile and 0.1% TFA, containing the peptides bradykinin (Brad, 10fmol/µL, MW = 904.46 kDa) and adrenocorticotropic hormone (ACTH, 40fmol/µL, MW = 2465.19 kDa)]. The standard peptides were used for calibration during the mass determination. The samples were analyzed on a MALDI-TOF/TOF mass spectrometer (MS), “Matrix assisted laser desorption/ionization - Time-of-Flight, Ultraflex III (Bruker). The analysis was performed in the “Reflectron” positive node. The detection mass range was between 880 – 3480, and the number of shots ca. 200-300. The spectrum calibration was done by quadratic fit using the internal calibrating peptides: ACTH (18–39), mass selected 2465.20 and des-Arg_Brad_MH+_mono, mass selected 904.47. The result was a list of values that correspond to the ratio mass/charge (m/z) of each peptide present in the tryptic digested sample. Peptide matching and protein searches were performed automatically with the use of in-house developed software “Poseidon” (32) or using the tool Mascot (www.matrixscience.com). The peptide masses (Peptide Mass Fingerprinting, PMF) were compared with the theoretical peptide masses of all available proteins from *Homo sapiens* specie in the database “SwissProt”. Monoisotopic masses were used and a mass tolerance of 0.0025% was allowed. The probability of a false positive match with a given MS-spectrum was determined for each analysis (Score). Unmatched peptides or miscleavage sites were not considered. Analysis in the MS/MS mode was performed using the instrument’s software. The significance threshold was set at P-value < 0.05. No mass and pI constraints were applied, and trypsin was set as enzyme. One missed cleavage per peptide was allowed, and carbamidomethylation was set as fixed modification while methionine oxidation as variable modification. Mass tolerance was set at 30ppm for MS spectra.

Functional analysis of proteins expressed in the myocardium was performed using the bioinformatics tools as the “Locus Link” (www.ncbi.nlm.nih.gov/locuslink) and based on the classification “Gene Ontology”. The names and acronyms, as well as the access number of proteins, were obtained by the tool UniProt (http://www.uniprot.org).

### Analysis of Protein Levels by Immunoblotting

Extracts of myocardial samples containing 30μg of protein were heated for 5 minutes at 95°C and subjected to one-dimensional electrophoresis (SDS-PAGE) using 12.5% polyacrylamide gel and the vertical electrophoresis system Ruby SE600 (GE Healthcare). After electrophoresis, proteins were transferred from the gel to a nitrocellulose membrane using the TE Semi-Dry Transfer Unit (GE Healthcare). The nitrocellulose membranes were incubated with monoclonal antibodies to specific proteins found with altered levels in the proteomic analysis: anti-CATA (catalase, mouse monoclonal antibody, 1 μg/mL, Sigma), anti-ACADVL (Very long-chain specific acyl-CoA dehydrogenase, mouse monoclonal antibody, 1 μg/mL, Abcam) and anti-DECR1 (2,4-dienoyl-CoA reductase 1, mouse monoclonal antibody, 1 μg/mL, Abcam). Each membrane was subjected to incubation with compatible secondary antibodies conjugated with peroxidase, developed using ECL Plus Western Blotting Detection Reagents (GE Healthcare) and detection using X-ray equipment. Analysis of densitometry was performed using the program ImageQuant TL (GE Healthcare). The relative abundance of a specific protein across the lanes of the blot was given by the normalization to the total amount of protein in each lane. Statistical analysis was performed with the Graphpad Prism software (Graphpad Software Inc, San Diego, USA). One-way ANOVA was used to compare the different unmatched groups, followed by the Tukey-Kramer test, which compares every mean with every other group mean and allows for the possibility of unequal sample sizes.

### Analysis of mRNA Expression by Real-Time Reverse Transcriptase (RT)-PCR

Total RNA from left ventricle samples was isolated using the RNeasy Fibrous tissue kit (Qiagen). Contaminating DNA was removed by treatment with RNase-free DNase I. cDNA was obtained from 5µg total RNA using Super-script II™ reverse transcriptase (Invitrogen). mRNA expression was analyzed by real-time quantitative reverse transcriptase (RT)-PCR with SYBR Green I PCR Master Mix (Applied Biosystems) and 250nM of sense and anti-sense primers using the ABI Prism 7500 Real Time PCR System (Applied Biosystems). The following primers were designed using Primer Express software version 3.0 (Applied Biosystems): HPRT1 (hypoxanthine phosphoribosyltransferase 1); (F) 5’ ATTAAAGCACTGAATAGAAAT 3’, (R) 3’ TAAAGATAAGTCACGAAATTA 5’, amplicon 108bp; ACADVL: (F) 5’-CCATACCCGTCCGTGCT-3’; (R) 5’-GAGCGTCATTCTTGGCGG-3’, amplicon 109bp; DERC1: (F) 5’ AAGCACAGAAAGGAGCAGCA 3’, (R) 5’ TCATGGCTTCCACACCTGC 3’, amplicon 108bp. After every PCR, an amplicon melting point curve was obtained. This yielded a single peak with the expected temperature provided by Primer Express software, confirming the specificity of the PCR. HPRT mRNA expression was used for normalization. The amount of mRNA in the left ventricle samples was calculated using the 2^-δCt^ method (33). Statistical analysis was also performed with the Graphpad Prism software (Graphpad Software Inc, San Diego, USA). One-way ANOVA was used to compare the different unmatched groups, followed by the Tukey-Kramer test, which compares every mean with every other group mean and allows for the possibility of unequal sample sizes.

### Lipid Peroxidation Evaluation

Lipid peroxidation levels were evaluated by the TBARS (thiobarbituric acid reactive substance) method based on the reaction of malonaldehyde (MDA), the major lipid oxidation product, with thiobarbituric acid (TBA), which leads to the formation of the TBA-MDA adduct (TBARS). The adduct has fluorescence property and was isolated and detected by high performance liquid chromatography (HPLC) with a fluorescence detector (34). Briefly, 50mg tissue was processed in 500µL TKM solution (Tris-HCl 50mM, KCl 25mM; MgCl_2_ 5mM; pH 7.5). For a 200µL aliquot of the tissue homogenate, further 200µL of TKM solution was added and subsequently 500μL of a 0.4%(w/v) solution of TBA in HCl 0.2N/H_2_O (2:1), 75μL of 0.2% (w/v) solution of BHT in 95% ethanol and 50μL of 10% (w/v) SDS. The mixture was heated to 90°C for 45 min, cooled on ice, and extracted with an equal volume of isobutanol. The isobutanolic phase was injected through a Shimadzu auto injector model SIL-10AD/VP (Shimadzu, Kyoto, Japan) in a Shimadzu HPLC system, consisting of two pumps LC-6AD connected to a Lichrosorb 10 RP-18 (Phenomenex, Torrance, CA) reversed-phase column. The flow rate was 0.8mL/min (5–25% acetonitrile in H_2_O deionized). The MDA-TBA adduct was detected with a RF-10A/XL fluorescence detector set at an excitation wavelength of 515nm and an emission wavelength of 550nm, and data were processed using Shimadzu ClassVP 5.03 software. Malonaldehyde-bisdiethylacetal was used as a standard. Statistical analysis was performed with the Graphpad Prism software (Graphpad Software Inc, San Diego, USA). One-way ANOVA was used to compare the different unmatched groups, followed by the Tukey-Kramer test, which compares every mean with every other group mean and allows for the possibility of unequal sample sizes.

### Effect of IFN-Gamma on Cardiomyocyte Mitochondrial Membrane Potential

Human adult ventricular cardiomyocyte cell line AC16 was propagated using DMEM:F12 supplemented with 12.5% inactivated fetal bovine serum (FBS) without antibiotics and used during 8 passages. A quantity of 0.10x10e4 cells were seeded per 0.34cm² and incubated in a humidified incubator at 37°C and 5% CO_2_ for 24 hours. Then, cells were treated with 5, 10 or 25ng/ml of IFN-gamma for 48 hours. At the end of treatment, cells were stained using 1µM of tetramethylrhodamine, methyl ester, perchlorate (TMRM, ThermoFisher Scientific), 400nM of mitotracker DeepRed (ThermoFisher Scientific), 500ng/ml of propidium iodide (PI, Sigma) and 1µM of Hoechst 33342 (ThermoFisher Scientific) at 37°C and 5% CO_2_ for 30 minutes and cells were washed once with Hanks’ Balanced Salt solution (HBSS) containing calcium and magnesium. Micrographs were captured using ImageXpress Micro XLS Widefield High-Content Analysis system at 100x magnification and the mitochondrial membrane potential was evaluated in live cells (PI negative) using and the web-based software Columbus 2.7.1.133403 (PerkinElmer Inc.). Mitochondrial ΔΨ was measured only in regions of co-localization of TMRE and mitotracker deepred fluorescences and data are reported as the ratio to not-treated cells. Cell viability was calculated as the ratio of the number of live cells (PI-negative) and total cells (PI-negative plus PI-positive cells) x 100; n=3.

## Results

We performed a differential proteomic analysis in the left ventricular free wall myocardial samples from patients with CCC, DCM and IC in comparison to subjects without cardiomyopathy (control group N), using a multiplexing methodology of 2-Dimentional Fluorescence Difference Gel Electrophoresis (2D-DIGE) (**Supplemental Figure S2**). Patients from the three cardiomyopathy groups included in the analysis displayed cardiomyocyte hypertrophy and fibrosis upon histopathological analysis, but lymphocytic myocarditis was only observed in myocardial samples from CCC patients (**Table 1**). No significant differences were found in age, ejection fraction (EF) or left ventricular diastolic diameter (LVDD) among the three cardiomyopathy groups. However, the control group – organ donors whose heart was not designated for transplantation – was inherently younger than the patient groups, since usually the preferential donors for organ transplantation are among younger individuals.

The proteomic analysis covered a total of 683 spots present in at least 80% of each of the fluorescent analytical gels. **Supplemental Figures S2A, B** display the Coomassie stained gel (preparative gel), and a representative 2D-DIGE stained gel (analytical gel), respectively. A total of 565 spots could be matched in the Coomassie colloidal blue stain-preparative gel and submitted to mass spectrometry identification; from which we were able to identify 439 proteins; and among those, 230 were distinct proteins (**Supplemental Table S2**). The majority of proteins (63%) were identified in a single spot. The remaining 37% of proteins have been identified in more than one spot. For some of these “multispot” proteins, only some of the spots were differentially expressed, while other spots corresponding to the same protein showed no difference in expression. This can be explained by the fact that some proteins could have different isoforms or post-translational modification that change their charge and consequently their isoelectric point. In this study, the deeper analysis of post-translational modification was not performed due to the complexity of such analysis and limitations of the method. **Supplemental Figures S3A–C** show Volcano plots displaying fold change and P values of the spots in the myocardial samples from CCC, DCM and IC patients as compared to the control group.

**Figure 1:**
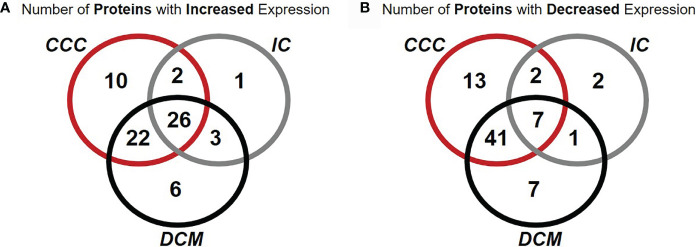
Venn diagrams representing the occurrence of proteins differentially expressed in common or unique relationships between groups of patients with cardiomyopathy group compared with individuals without cardiomyopathies. Number of proteins with increased **(A)** or decreased **(B)** expression of at least one spot in samples from patients when compared with samples from subjects without cardiomyopathy. Over 67% of proteins differentially expressed in CCC were contained in a single spot.

**Figure 1** and **Supplemental Figure S4** show Venn diagrams indicating the number of differentially expressed identified proteins and spots, that were shared or unique in any of the disease groups as compared to the control group. When compared with the control group, myocardial samples from patients with CCC and DCM showed a higher number of differentially expressed proteins than the IC group. Approximately 77% of differentially expressed proteins in CCC myocardium were shared with the DCM group, 29% were shared with the IC group, and 26% of differentially expressed proteins were shared between the 3 groups.

**Figure 2:**
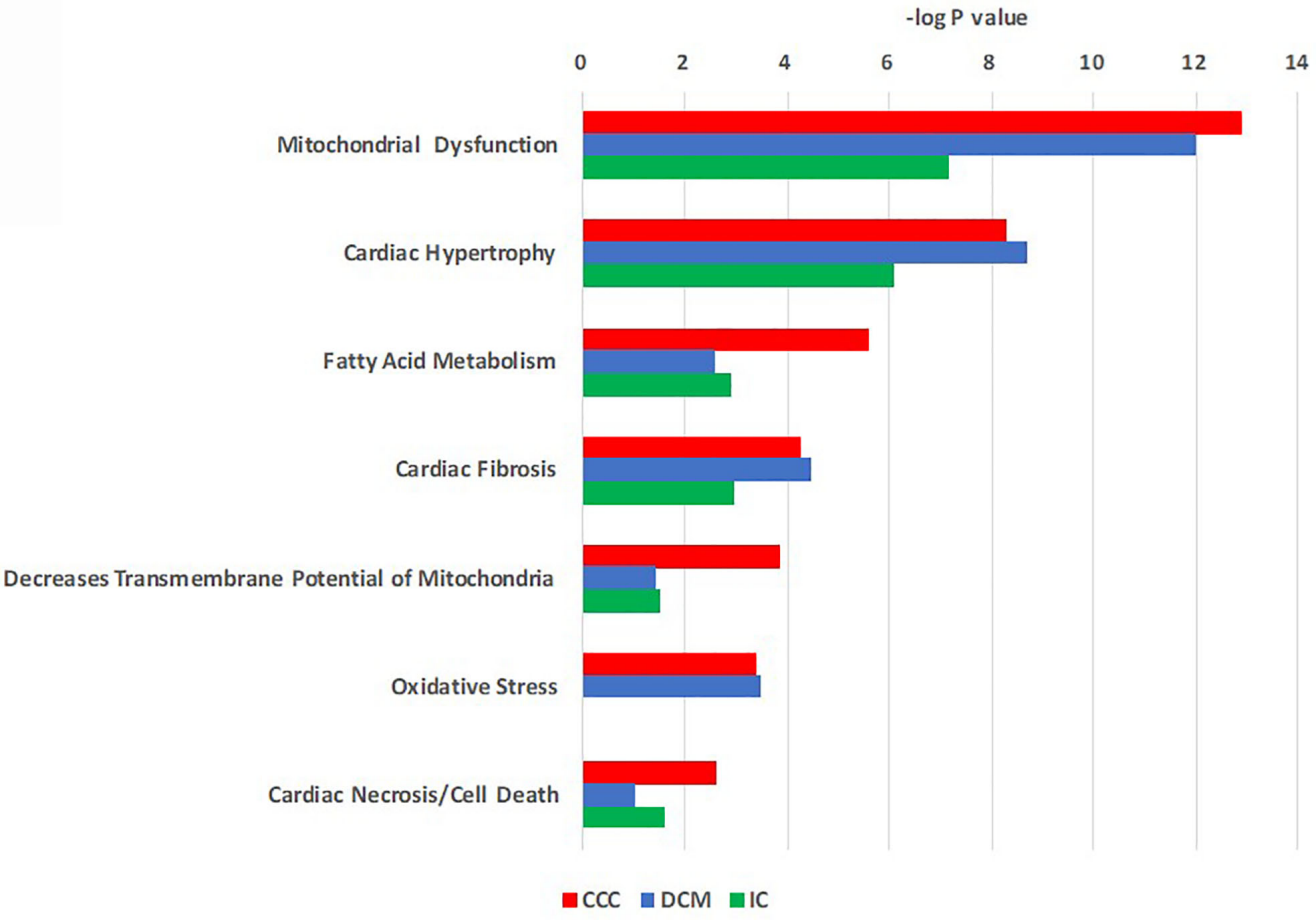
Toxicity function pathways analysis of differentially expressed proteins in CCC, DCM and IC myocardium. Proteins differentially expressed in heart tissue were analyzed using Ingenuity Pathways Analysis^®^ (Qiagen) using the tox-list function, which classifies gene or protein sets into pathological/toxicological pathways. Bars indicate the –log p value for a given pathway or process.

Pathways analysis of differentially expressed proteins (**Figure 2**) disclosed enrichment in cardiac hypertrophy and cardiac fibrosis for all 3 diseases, while oxidative stress was enriched for both CCC and DCM. We observed a higher enrichment in CCC and DCM in the mitochondrial dysfunction pathway, and much higher enrichment in CCC in the fatty acid metabolism (beta-oxidation), transmembrane potential of mitochondria, and cardiac necrosis pathways. The compilation of the data from all analyzed spots and proteins can be found in **Supplemental Table S2**. **Table 2** and **Supplemental Figure S5A** shows the cellular component classification of the total identified proteins. Most of the identified proteins were classified as cytoplasmic (35%), mitochondrial (28%), or cytoskeletal (13%). **Table 3** and **Supplemental Figure S5B** shows the functional classification of the total identified proteins. Through this classification, most of the proteins were classified as proteins related to metabolism (37%), structural and contractile proteins (14%) and proteins involved in the stress response and apoptosis (12%). Analysis of differentially expressed proteins in samples from each clinical group disclosed a similar distribution, as can also be observed in the **Tables 2** and **3**.

**Table 2:** Cellular component classification of the proteins differentially expressed in the myocardium from patients with CCC, IC and DCM.

Cellular Component	Total prot.^*^	T. diff. prot.^†^	CCC/N		IC/N		DCM/N	
			↑	↓	↑	↓	↑	↓
Cytoplasm	81	54	25	22	9	3	21	25
Cytoskeleton	30	22	6	11	3	5	7	9
Endoplasmic reticulum	8	5	5	0	0	0	5	0
Membrane	13	8	3	4	2	0	2	3
Mitochondria	64	41	13	22	13	3	14	17
Nucleus	20	8	3	4	0	0	3	3
Secreted	10	8	6	0	5	0	7	0
Other	4	3	2	0	1	1	1	0
**Total**	**230**	**149**	**63**	**63**	**33**	**12**	**60**	**57**

*Total prot., Total number of proteins identified in each cellular component; ^†^T. diff. prot., Total number of proteins in each cellular component that were found to be differentially expressed in the myocardium from patients with CCC, IC and DCM as compared to individuals without cardiomyopathies (N).

Bold means the total number of protein in each column.

↑ means upregulated expression ↓ means downregulated expression. Bold means the total number of protein in each column.

**Table 3:** Functional classification of the proteins differentially expressed in the myocardium from patients with CCC, IC and DCM.

Function	Total prot.^*^	T. diff. prot.^†^	CCC/N		IC/N		DCM/N	
			↑	↓	↑	↓	↑	↓
1. Structural and Contractile Proteins	32	25	7	13	4	5	8	10
2. Metabolism	86	60	19	32	16	6	19	29
2.1. Glycolysis	15	11	5	5	3	0	3	6
2.2. Lipid Metabolism/ß-Oxidation	11	7	0	6	1	1	1	2
2.3. Tricarboxylic Acid Cycle	8	7	1	4	1	0	2	3
2.4. Oxidative Phosphorylation and Electron Transport	18	14	5	8	5	2	4	8
2.5. Creatine Kinase System (Energy Transduction)	3	2	0	2	0	1	0	2
2.6. Other Metabolic Processes	31	19	8	7	6	2	9	8
3. Stress Response and Apoptosis	27	22	13	6	2	0	12	6
4. Immune Response	3	3	3	0	2	0	2	0
5. Cell Signaling	13	4	2	1	0	0	1	2
6. Transcription and Translation Processes	18	6	2	2	0	0	3	3
7. Transport	8	3	2	1	2	0	2	1
8. Proteasome-Ubiquitin Process	6	3	3	0	0	0	2	0
9. Other Functions	37	23	12	8	7	1	11	6
**Total**	**230**	**149**	**63**	**63**	**33**	**12**	**60**	**57**

*Total prot., Total number of proteins identified in each functional classification; ^†^T. diff. prot., Total number of proteins in each functional classification that were found to be differentially expressed in the myocardium from patients with CCC, IC and DCM as compared to individuals without cardiomyopathies (N).

↑ means upregulated expression ↓ means downregulated expression. Bold means the total number of protein in each column

**Table 4** displays the differentially expressed proteins in the different clinical groups (CCC, DCM or IC) as compared with individuals without cardiomyopathy (control group N), classified by biological process, and shows which of these proteins were shared among the groups. We found 128 differentially expressed proteins in myocardial samples from patients with CCC when compared to the control group (**Table 4**, section a). Among these, 25 proteins (ca. 20%) were found exclusively modulated in CCC samples. Among the 25, 12 were upregulated; between those, 3 belonged to the stress response/apoptosis process and 2 to metabolism (glycolysis). Thirteen proteins were down-modulated exclusively in CCC myocardium. Five belonged to the mitochondrial energy metabolism (oxidation, tricarboxylic acid cycle and oxidative phosphorylation), 4 were structural/contractile proteins, and one belonged to the stress response and apoptosis process. Regarding IC (**Table 4**, section b) and DCM (**Table 4**, section c), we identified 113 and 44 differentially expressed proteins as compared to the control group. The **Table 4** also includes the list of the protein that were differentially expressed in more than one specific disease group as compared to control group.

**Table 4:** List of the differentially expressed proteins identified in the myocardium from patients with CCC, IC and DCM when compared to individuals without cardiomyopathies.

a) Differentially Expressed Proteins Only in CCC patients			Total Spots^‡^	CCC/N		IC/N		DCM/N	
Entry name*	Protein names^†^	Function		↑↓	S^§^	↑↓	S^§^	↑↓	S^§^
ARP3	Actin-like protein 3	1. Structural and Contractile Proteins	1	**↑**	1				
ENOA	Alpha-enolase (Non- neural enolase)	2.1. Glycolysis	6	**↑**	1				
G3P	Glyceraldehyde-3-phosphate dehydrogenase (GAPDH)	2.1. Glycolysis	7	**↑**	2				
MCCB	Methylcrotonoyl-CoA carboxylase beta chain, mitochondrial	2.6. Other Metabolic Processes	1	**↑**	1				
CATA	Catalase	3. Stress Response and Apoptosis	2	**↑**	2				
HSPB7	Heat shock protein beta-7	3. Stress Response and Apoptosis	1	**↑**	1				
TR10B	Tumor necrosis factor receptor superfamily member 10B	3. Stress Response and Apoptosis	1	**↑**	1				
LEG3	Galectin-3	4. Immune Response	1	**↑**	1				
BLK	Tyrosine-protein kinase BLK	5. Cell Signaling	1	**↑**	1				
PSME1	Proteasome activator complex subunit 1 (Interferon gamma up-regulated I-5111 protein)	8. Proteasome-Ubiquitin Process/Immune response	1	**↑**	1				
APOA1	Apolipoprotein A-I precursor	9. Other Functions	2	**↑**	1				
F90AM	Putative protein FAM90A22	9. Other Functions	1	**↑**	1				
ACTA	Actin, aortic smooth muscle (Alpha-actin-2)	1. Structural and Contractile Proteins	1	**↓**	1				
MYL4	Myosin light polypeptide 4 (Myosin light chain 1, embryonic muscle/atrial isoform)	1. Structural and Contractile Proteins	1	**↓**	1				
STML2	Stomatin-like protein 2	1. Structural and Contractile Proteins	1	**↓**	1				
TPM1	Tropomyosin-1 alpha chain	1. Structural and Contractile Proteins	1	**↓**	1				
ACADVL	Very long-chain specific acyl-CoA dehydrogenase, mitochondrial	2.2. Lipid Metabolism/ß-Oxidation	5	**↓**	3				
HCDH	Hydroxyacyl-coenzyme A dehydrogenase, mitochondrial	2.2. Lipid Metabolism/ß-Oxidation	4	**↓**	4				
THIM	3-ketoacyl-CoA thiolase, mitochondrial	2.2. Lipid Metabolism/ß-Oxidation	2	**↓**	1				
MDHM	Malate dehydrogenase, mitochondrial	2.3. Tricarboxylic Acid Cycle	4	**↓**	3				
NDUS1	NADH-ubiquinone oxidoreductase 75 kDa subunit, mitochondrial	2.4. Oxidative Phosphorylation and Electron Transport	3	**↓**	2				
ROA2	Heterogeneous nuclear ribonucleoproteins A2/B1	2.6. Other Metabolic Processes	2	**↓**	1				
HSPB1	Heat-shock protein beta-1 (Heat shock 27 kDa protein)	3. Stress Response and Apoptosis	3	**↓**	1				
KELL	Kell blood group glycoprotein	9. Other Functions	1	**↓**	1				
SKT	Sickle tail protein homolog	9. Other Functions	1	**↓**	1				
b) Differentially Expressed Proteins Only in IC patients			Total Spots^‡^	CCC/N		IC/N		DCM/N	
Entry name*	Protein names^†^	Function			↑↓	S^§^	↑↓	S^§^	↑↓	S^§^
ACON	Aconitate hydratase, mitochondrial (Aconitase)	2.3. Tricarboxylic Acid Cycle	8			**↑**	3		
KI26A	Kinesin-like protein KIF26A	1. Structural and Contractile Proteins	1			**↓**	1		
W19L5	Putative WBSCR19-like protein 5	9. Other Functions	1			**↓**	1		
c) Differentially Expressed Proteins Only in DCM patients			Total Spots^‡^	CCC/N		IC/N		DCM/N	
Entry name*	Protein names^†^	Function		↑↓	S^§^	↑↓	S^§^	↑↓	S^§^
PDLI1	PDZ and LIM domain protein 1	1. Structural and Contractile Proteins	1					**↑**	1
VINC	Vinculin (Metavinculin)	1. Structural and Contractile Proteins	2					**↑**	2
ODO2	2-oxoglutarate dehydrogenase E2 component, mitochondrial	2.3. Tricarboxylic Acid Cycle	2					**↑**	1
COQ9	Ubiquinone biosynthesis protein COQ9, mitochondrial	2.6. Other Metabolic Processes	1					**↑**	1
HSP7C	Heat shock cognate 71 kDa protein (Heat shock 70 kDa protein 8).	3. Stress Response and Apoptosis	2					**↑**	2
SAMP	Serum amyloid P-component precursor	3. Stress Response and Apoptosis	1					**↑**	1
ZN799	Zinc finger protein 799	6. Transcription and Translation Processes	1					**↑**	1
IFIT5	Interferon-induced protein with tetratricopeptide repeats 5	9. Other Functions	1					**↑**	1
CAZA2	F-actin capping protein subunit alpha-2 (CapZ alpha-2)	1. Structural and Contractile Proteins	1					**↓**	1
MYOZ2	Myozenin-2	1. Structural and Contractile Proteins	3					**↓**	1
TPIS	Triosephosphate isomerase	2.1. Glycolysis	3					**↓**	1
NDUAD	NADH dehydrogenase [ubiquinone] 1 alpha subcomplex subunit 13	2.4. Oxidative Phosphorylation and Electron Transport	1					**↓**	1
GSTM2	Glutathione S-transferase Mu 2	2.6. Other Metabolic Processes	1					**↓**	1
CRYAB	Alpha crystallin B chain	3. Stress Response and Apoptosis	3					**↓**	1
GBG5	Guanine nucleotide-binding protein G(I)/G(S)/G(O) subunit gamma-5	5. Cell Signaling	2					**↓**	1
EDC4	Enhancer of mRNA-decapping protein 4	6. Transcription and Translation Processes	1					**↓**	1
d) Differentially Expressed Proteins Shared by CCC and IC patients			Total Spots^‡^	CCC/N		IC/N		DCM/N	
Entry name*	Protein names^†^	Function			↑↓	S^§^	↑↓	S^§^	↑↓	S^§^
NDUS3	NADH dehydrogenase [ubiquinone] iron-sulfur protein 3, mitochondrial	2.4. Oxidative Phosphorylation and Electron Transport	2	**↑**	1	**↑**	1		
CHCH3	Coiled-coil-helix-coiled-coil-helix domain-containing protein 3, mitochondrial	9. Other Functions	2	**↑**	1	**↑**	1		
ACTN2	Alpha-actinin-2 (Alpha actinin skeletal muscle isoform 2)	1. Structural and Contractile Proteins	4	**↓**	1	**↓**	1		
D3D2	3,2-trans-enoyl-CoA isomerase, mitochondrial	2.2. Lipid Metabolism/ß-Oxidation	1	**↓**	1	**↓**	1		
e) Differentially Expressed Proteins Shared by CCC and DCM patients			Total Spots^‡^	CCC/N		IC/N		DCM/N	
Entry name*	Protein names^†^	Function			↑↓	S^§^	↑↓	S^§^	↑↓	S^§^
TBA1C	Tubulin alpha-1C chain	1. Structural and Contractile Proteins	1	**↑**	1			**↑**	1
TBB5	Tubulin beta-5 chain	1. Structural and Contractile Proteins	3	**↑**	2			**↑**	2
IDH3A	Isocitrate dehydrogenase [NAD] subunit alpha, mitochondrial	2.3. Tricarboxylic Acid Cycle	2	**↑**	2			**↑**	1
DPYL2	Dihydropyrimidinase-related protein 2	2.6. Other Metabolic Processes	1	**↑**	1			**↑**	1
SCOT1	Succinyl-CoA:3-ketoacid-coenzyme A transferase 1, mitochondrial	2.6. Other Metabolic Processes	2	**↑**	1			**↑**	2
ENPL	Endoplasmin precursor (Heat shock protein 90 kDa beta member 1)	3. Stress Response and Apoptosis	2	**↑**	1			**↑**	1
GRP78	78 kDa glucose-regulated protein (Heat shock 70 kDa protein 5)	3. Stress Response and Apoptosis	3	**↑**	3			**↑**	3
HS90A	Heat shock protein HSP 90-alpha	3. Stress Response and Apoptosis	1	**↑**	1			**↑**	1
HSP71	Heat shock 70 kDa protein 1	3. Stress Response and Apoptosis	2	**↑**	1			**↑**	2
PDIA1	Protein disulfide-isomerase precursor	3. Stress Response and Apoptosis	1	**↑**	1			**↑**	1
PDIA3	Protein disulfide-isomerase A3 precursor	3. Stress Response and Apoptosis	2	**↑**	2			**↑**	2
ANXA1	Annexin A1 (Annexin I)	3. Stress Response and Apoptosis	1	**↑**	1			**↑**	1
ANXA5	Annexin A5 (Annexin V)	3. Stress Response and Apoptosis	2	**↑**	2			**↑**	2
1433Z	14-3-3 protein zeta/delta	5. Cell Signaling	1	**↑**	1			**↑**	1
SYAC	Alanyl-tRNA synthetase, cytoplasmic	6. Transcription and Translation Processes	3	**↑**	2			**↑**	1
ZN658	Zinc finger protein 658	6. Transcription and Translation Processes	1	**↑**	1			**↑**	1
MIB2	E3 ubiquitin-protein ligase MIB2	8. Proteasome-Ubiquitin Process	1	**↑**	1			**↑**	1
RNF25	E3 ubiquitin-protein ligase	8. Proteasome-Ubiquitin Process	1	**↑**	1			**↑**	1
ANXA2	Annexin A2 (Annexin II)	9. Other Functions	3	**↑**	3			**↑**	2
F13A	Coagulation factor XIII A chain	9. Other Functions	1	**↑**	1			**↑**	1
AMPL	Cytosol aminopeptidase	9. Other Functions	2	**↑**	2			**↑**	1
PDZD4	PDZ domain-containing protein 4	9. Other Functions	1	**↑**	1			**↑**	1
AINX	Alpha-internexin	1. Structural and Contractile Proteins	1	**↓**	1			**↓**	1
MLRV	Myosin regulatory light chain 2, ventricular/cardiac muscle isoform	1. Structural and Contractile Proteins	4	**↓**	1			**↓**	3
MYL3	Myosin light polypeptide 3 (Myosin light chain 1, slow-twitch muscle B/ventricular isoform)	1. Structural and Contractile Proteins	4	**↓**	2			**↓**	2
TNNT2	Troponin T, cardiac muscle	1. Structural and Contractile Proteins	7	**↓**	1			**↓**	1
VIME	Vimentin	1. Structural and Contractile Proteins	1	**↓**	1			**↓**	1
ALDOA	Fructose-bisphosphate aldolase A (Muscle-type aldolase)	2.1. Glycolysis	6	**↓**	1			**↓**	2
ALDOC	Fructose-bisphosphate aldolase C (Brain-type aldolase)	2.1. Glycolysis	2	**↓**	2			**↓**	2
ENOB	Beta-enolase (Skeletal muscle enolase)	2.1. Glycolysis	1	**↓**	1			**↓**	1
K6PF	6-phosphofructokinase, muscle type	2.1. Glycolysis	2	**↓**	1			**↓**	1
PGAM2	Phosphoglycerate mutase 2	2.1. Glycolysis	2	**↓**	2			**↓**	2
ACADM	Medium-chain specific acyl-CoA dehydrogenase, mitochondrial	2.2. Lipid Metabolism/ß-Oxidation	1	**↓**	1			**↓**	1
DECR	2,4-dienoyl-CoA reductase, mitochondrial	2.2. Lipid Metabolism/ß-Oxidation	3	**↓**	1			**↓**	2
DHSB	Succinate dehydrogenase [ubiquinone] iron-sulfur subunit, mitochondrial	2.3. Tricarboxylic Acid Cycle	1	**↓**	1			**↓**	1
FUMH	Fumarate hydratase, mitochondrial	2.3. Tricarboxylic Acid Cycle	3	**↓**	1			**↓**	1
IDHP	Isocitrate dehydrogenase [NADP], mitochondrial	2.3. Tricarboxylic Acid Cycle	3	**↓**	3			**↓**	3
ATP5H	ATP synthase D chain, mitochondrial	2.4. Oxidative Phosphorylation and Electron Transport	1	**↓**	1			**↓**	1
NDUAA	NADH dehydrogenase [ubiquinone] 1 alpha subcomplex subunit 10	2.4. Oxidative Phosphorylation and Electron Transport	1	**↓**	1			**↓**	1
NDUV1	NADH dehydrogenase [ubiquinone] flavoprotein 1, mitochondrial	2.4. Oxidative Phosphorylation and Electron Transport	4	**↓**	2			**↓**	1
QCR1	Cytochrome b-c1 complex subunit 1, mitochondrial	2.4. Oxidative Phosphorylation and Electron Transport	3	**↓**	2			**↓**	1
QCR2	Cytochrome b-c1 complex subunit 2, mitochondrial	2.4. Oxidative Phosphorylation and Electron Transport	1	**↓**	1			**↓**	1
KCRS	Creatine kinase, sarcomeric mitochondrial	2.5. Creatine Kinase System (Energy Transduction)	5	**↓**	1			**↓**	1
KU86	ATP-dependent DNA helicase 2 subunit 2	2.6. Other Metabolic Processes	1	**↓**	1			**↓**	1
BLVRB	Flavin reductase	2.6. Other Metabolic Processes	1	**↓**	1			**↓**	1
KAD1	Adenylate kinase isoenzyme 1	2.6. Other Metabolic Processes	2	**↓**	1			**↓**	1
MDHC	Malate dehydrogenase, cytoplasmic	2.6. Other Metabolic Processes	3	**↓**	1			**↓**	1
THIL	Acetyl-CoA acetyltransferase, mitochondrial	2.6. Other Metabolic Processes	1	**↓**	1			**↓**	1
AKT2	RAC-beta serine/threonine-protein kinase	3. Stress Response and Apoptosis	1	**↓**	1			**↓**	1
NEIL1	Endonuclease VIII-like 1	3. Stress Response and Apoptosis	1	**↓**	1			**↓**	1
PRDX2	Peroxiredoxin-2	3. Stress Response and Apoptosis	1	**↓**	1			**↓**	1
PRDX3	Peroxiredoxin-3	3. Stress Response and Apoptosis	1	**↓**	1			**↓**	1
PRDX6	Peroxiredoxin-6	3. Stress Response and Apoptosis	3	**↓**	1			**↓**	3
KC1E	Casein kinase I isoform epsilon	5. Cell Signaling	1	**↓**	1			**↓**	1
EIF3J	Eukaryotic translation initiation factor 3 subunit	6. Transcription and Translation Processes	1	**↓**	1			**↓**	1
IF4H	Eukaryotic translation initiation factor 4H	6. Transcription and Translation Processes	1	**↓**	1			**↓**	1
UCP2	Mitochondrial uncoupling protein 2	7. Transport	1	**↓**	1			**↓**	1
EHD4	EH domain-containing protein 4	9. Other Functions	1	**↓**	1			**↓**	1
GAB3	GRB2-associated-binding protein 3	9. Other Functions	1	**↓**	1			**↓**	1
JKIP1	Janus kinase and microtubule-interacting protein 1	9. Other Functions	1	**↓**	1			**↓**	1
MYG	Myoglobin	9. Other Functions	8	**↓**	2			**↓**	3
PEBP1	Phosphatidylethanolamine-binding protein 1	9. Other Functions	1	**↓**	1			**↓**	1
WDFY2	WD repeat and FYVE domain-containing protein 2	9. Other Functions	1	**↓**	1			**↓**	1
f) Differentially Expressed Proteins Shared by IC and DCM patients			Total Spots^‡^	CCC/N		IC/N		DCM/N	
Entry name*	Protein names^†^	Function			↑↓	S^§^	↑↓	S^§^	↑↓	S^§^
ECHM	Enoyl-CoA hydratase, mitochondrial	2.2. Lipid Metabolism/ß-Oxidation	1			**↑**	1	**↑**	1
AOFB	Amine oxidase [flavin-containing] B	2.6. Other Metabolic Processes	1			**↑**	1	**↑**	1
BPIL3	Bactericidal/permeability-increasing protein-like 3	9. Other Functions	1			**↑**	1	**↑**	1
GSTP1	Glutathione S-transferase P	2.6. Other Metabolic Processes	1			**↓**	1	**↓**	1
g) Differentially Expressed Proteins Shared by CCC, IC and DCM patients			Total Spots^‡^	CCC/N		IC/N		DCM/N	
Entry name*	Protein names^†^	Function			↑↓	S^§^	↑↓	S^§^	↑↓	S^§^
DESM	Desmin	1. Structural and Contractile Proteins	9	**↑**	4	**↑**	2	**↑**	8
GELS	Gelsolin precursor (Actin-depolymerizing factor)	1. Structural and Contractile Proteins	6	**↑**	5	**↑**	1	**↑**	5
LUM	Lumican precursor	1. Structural and Contractile Proteins	1	**↑**	1	**↑**	1	**↑**	1
TNNI3	Troponin I, cardiac muscle	1. Structural and Contractile Proteins	1	**↑**	1	**↑**	1	**↑**	1
G6PI	Glucose-6-phosphate isomerase	2.1. Glycolysis	1	**↑**	1	**↑**	1	**↑**	1
KPYM	Pyruvate kinase isozymes M1/M2	2.1. Glycolysis	4	**↑**	1	**↑**	1	**↑**	3
PGAM1	Phosphoglycerate mutase 1	2.1. Glycolysis	2	**↑**	2	**↑**	2	**↑**	2
AL4A1	Delta-1-pyrroline-5-carboxylate dehydrogenase, mitochondrial	2.4. Oxidative Phosphorylation and Electron Transport	1	**↑**	1	**↑**	1	**↑**	1
ATPA	ATP synthase subunit alpha, mitochondrial	2.4. Oxidative Phosphorylation and Electron Transport	8	**↑**	4	**↑**	3	**↑**	3
DHSA	Succinate dehydrogenase [ubiquinone] flavoprotein subunit, mitochondrial	2.4. Oxidative Phosphorylation and Electron Transport	4	**↑**	2	**↑**	2	**↑**	2
DLDH	Dihydrolipoyl dehydrogenase, mitochondrial precursor	2.4. Oxidative Phosphorylation and Electron Transport	4	**↑**	3	**↑**	1	**↑**	3
CATD	Cathepsin D	2.6. Other Metabolic Processes	2	**↑**	1	**↑**	1	**↑**	1
DHE3	Glutamate dehydrogenase 1, mitochondrial	2.6. Other Metabolic Processes	1	**↑**	1	**↑**	1	**↑**	1
MMSA	Methylmalonate-semialdehyde dehydrogenase [acylating], mitochondrial	2.6. Other Metabolic Processes	1	**↑**	1	**↑**	1	**↑**	1
TGM2	Protein-glutamine gamma-glutamyltransferase 2	2.6. Other Metabolic Processes	3	**↑**	3	**↑**	1	**↑**	3
UGPA	UTP–glucose-1-phosphate uridylyltransferase	2.6. Other Metabolic Processes	1	**↑**	1	**↑**	1	**↑**	1
GRP75	Stress-70 protein, mitochondrial	3. Stress Response and Apoptosis	2	**↑**	2	**↑**	2	**↑**	2
TRAF3	TNF receptor-associated factor 3	3. Stress Response and Apoptosis	2	**↑**	1	**↑**	1	**↑**	2
CO3	Complement C3 precursor	4. Immune Response	2	**↑**	1	**↑**	1	**↑**	2
IGHG1	Ig gamma-1 chain C region.	4. Immune Response	3	**↑**	3	**↑**	3	**↑**	3
FRRS1	Ferric-chelate reductase 1	7. Transport	1	**↑**	1	**↑**	1	**↑**	1
SNAB	Beta-soluble NSF attachment protein	7. Transport	1	**↑**	1	**↑**	1	**↑**	1
ALBU	Serum albumin precursor	9. Other Functions	4	**↑**	4	**↑**	2	**↑**	4
CSRP3	Cysteine and glycine-rich protein 3	9. Other Functions	1	**↑**	1	**↑**	1	**↑**	1
IMMT	Mitochondrial inner Membrane protein (Mitofilin)	9. Other Functions	4	**↑**	3	**↑**	2	**↑**	3
TRFE	Serotransferrin precursor (Transferrin)	9. Other Functions	4	**↑**	3	**↑**	3	**↑**	3
YP016	Uncharacterized protein MGC16385	9. Other Functions	1	**↑**	1	**↑**	1	**↑**	1
ACTC	Actin, alpha cardiac muscle 1 (Alpha-cardiac actin)	1. Structural and Contractile Proteins	15	**↓**	8	**↓**	1	**↓**	5
LDB3	LIM domain-binding protein 3	1. Structural and Contractile Proteins	5	**↓**	5	**↓**	5	**↓**	5
MYH7	Myosin-7 (Myosin heavy chain, cardiac muscle beta isoform)	1. Structural and Contractile Proteins	2	**↓**	1	**↓**	1	**↓**	1
AT5F1	ATP synthase subunit b, mitochondrial	2.4. Oxidative Phosphorylation and Electron Transport	3	**↓**	2	**↓**	1	**↓**	2
NDUV2	NADH dehydrogenase [ubiquinone] flavoprotein 2, mitochondrial	2.4. Oxidative Phosphorylation and Electron Transport	1	**↓**	1	**↓**	1	**↓**	1
KCRM	Creatine kinase M-type	2.5. Creatine Kinase System (Energy Transduction)	5	**↓**	5	**↓**	2	**↓**	5
AATC	Aspartate aminotransferase, cytoplasmic	2.6. Other Metabolic Processes	3	**↓**	2	**↓**	2	**↓**	2

*Entry name: Mnemonic identifier for a UniProtKB entry, all the entry names are followed by “_HUMAN”; ^†^Protein names: Name of the protein according to UniProtKB; ^‡^Total spots: Number of spots identified as such protein; §S: Number of differentially expressed spots identified as such protein for each comparison. Other annotations and comments, fold change and statistical values, as well as protein identification score values of all identified spots are included in the **Supplemental Table 2** from Online **Supplemental Data**.

↑ means upregulated expression ↓ means downregulated expression.

**Figure 3:**
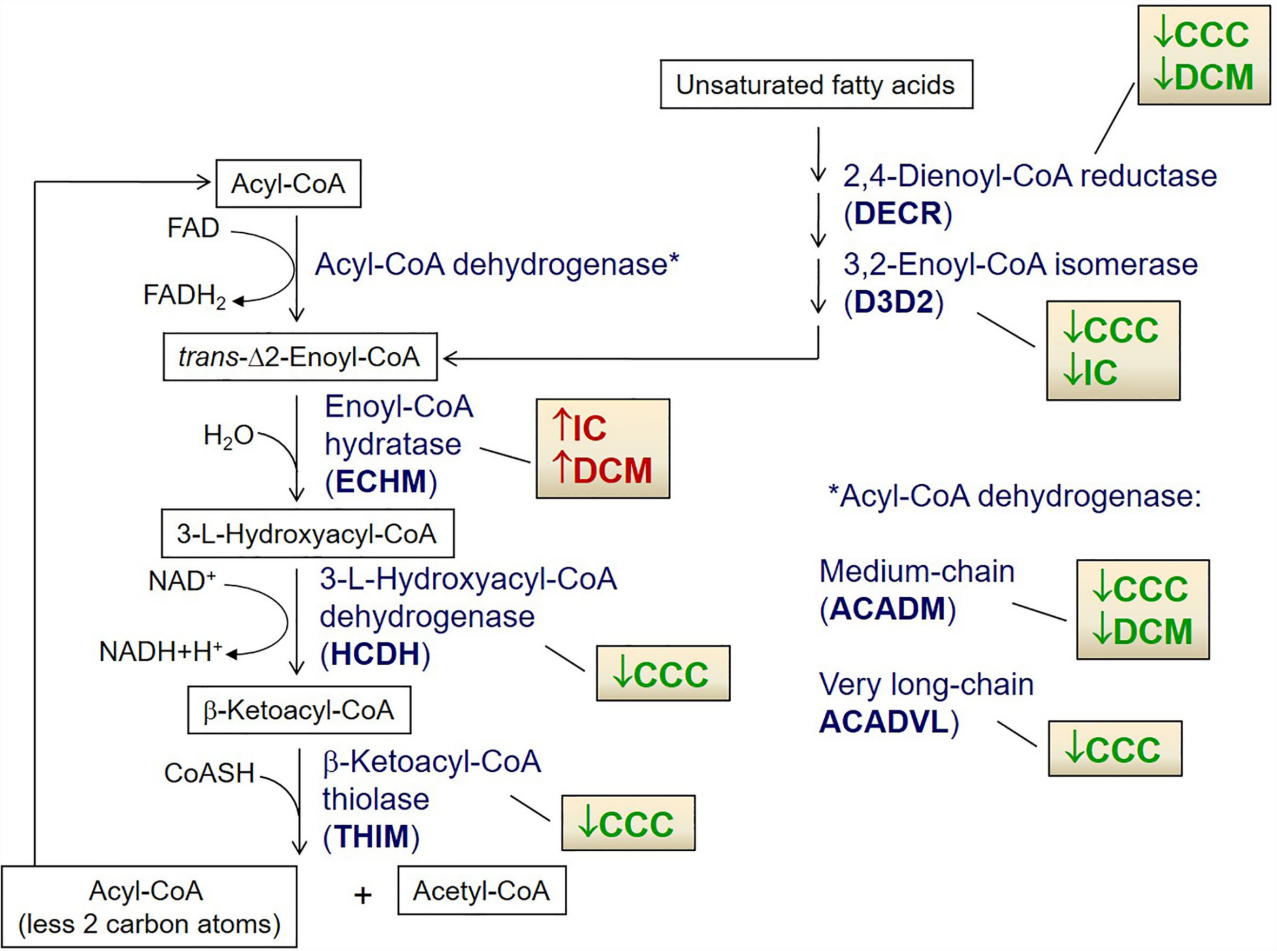
Cartoon depicting fatty acid β-oxidation enzymes differentially expressed in CCC, DCM and IC myocardium. Arrows indicate up-regulated (red) or down-regulated (green) expression as compared to control myocardial samples in the proteomic analysis. *Indicates the right place for in the metabolic pathway for Acyl coA dehydrogenases ACADVL and ACADM.

**Figure 4:**
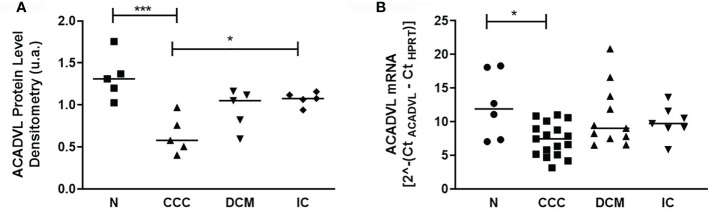
Protein and mRNA levels of ACADVL (Very long-chain specific acyl-CoA dehydrogenase) in myocardial tissue of CCC, DCM, IC and controls. **(A)** Densitometry measurement of ACADVL protein levels using immunoblot (One-Way ANOVA p = 0.0011). **(B)**. ACADVL mRNA levels assessed using real time RT-qPCR (One-Way ANOVA p = 0.0154). The horizontal lines show statistically significant changes between groups by the Tukey-Kramer test: *p < 0.05; ***p < 0.001.

We then focused the analysis on metabolism-related proteins because they were the most frequently modulated in our study. Among the energy metabolism proteins with reduced expression only in CCC samples, we found several enzymes from the beta-oxidation process, as depicted in **Figure 3**. Very long-chain specific acyl-CoA dehydrogenase (ACADVL) was found in decreased levels in 3 out of 5 spots only in CCC samples. Moreover, the total level of ACADVL was also found differentially expressed when evaluated by immunoblotting (**Figure 4A** and **Supplemental Figure S9**). CCC patients show reduced total levels of ACADVL as compared to samples from the control group and from patients with IC. mRNA levels of the ACADVL gene (**Figure 4B**) were also reduced in CCC samples as compared to the control group, suggesting transcriptional regulation. Moreover, all spots identified as hydroxyacyl-coenzyme A dehydrogenase (HCDH) and one out of two spots identified as the protein 3-ketoacyl-CoA thiolase (THIM) were found to be decreased only in CCC samples as compared to samples from individuals without cardiomyopathies. Medium-chain specific acyl-CoA dehydrogenase (ACADM) showed decreased levels in CCC and also DCM samples. The enzymes 2,4-dienoyl-CoA reductase (DECR) (**Supplemental Figure S9**) and 3,2-trans-enoyl-CoA isomerase (D3D2), which participate in the metabolism of unsaturated fatty enoyl-CoA esters, also showed reduced expression in samples of patients with CCC and DCM, and in samples of patients with CCC and IC, respectively, when compared to control samples. Enoyl-CoA hydratase (ECHM), which participates in the second reaction of beta-oxidation, shows increased expression in patients with IC and DCM, but not in patients with CCC. We further evaluate by immunoblotting the protein levels of the protein DECR, that presented decreased levels in CCC samples as compared to IC samples and samples from the control group. This reduction was also observed in the DECR mRNA level evaluated by RT-qPCR (**Figure S6**). Taken together, the number of beta-oxidation enzymes with reduced expression in CCC is considerable higher than DCM and IC samples (6, 2 and 1, respectively) (**Table 3**), which may indicate that fatty acid beta-oxidation is more impaired in CCC than DCM or IC.

Evaluating further other pathways from the energetic metabolism, nine proteins involved in glycolysis were differentially expressed in the cardiomyopathy groups (**Supplemental Figure S7A**). Three were upregulated in all patient samples, while ENOA and G3PDH were exclusively upregulated in CCC. Five enzymes showed reduced expression in both CCC and DCM (**Table 4**).

Regarding the tricarboxylic acid cycle (**Supplemental Figure S7B**), six enzymes were differentially expressed in the cardiomyopathies; three of them with reduced expression in CCC and DCM samples as compared to control group: Fumarate hydratase (FUMH); Succinate dehydrogenase [ubiquinone] iron-sulfur subunit (DHSB) and Isocitrate dehydrogenase [NADP] (IDHP). IDHP participates in the tricarboxilic acid cycle, but is also involved in antioxidant mechanisms. Moreover, we observed reduced levels of the protein Malate dehydrogenase (MDHM) exclusively in the CCC group. On the other hand, we found increased protein levels of Isocitrate dehydrogenase [NAD] subunit alpha (IDH3A) in CCC and DCM samples. Aconitate hydratase, mitochondrial (Aconitase, ACON) had increased levels in the IC samples only.

Moving to the energy production by the respiratory chain pathway, we identified 13 differentially expressed proteins belonging to complexes I, II, III and V of the oxidative phosphorylation process (**Table 4** and **Supplemental Figure S7C**). Ten of them, including uncoupling protein 2 (UCP2), were reduced in CCC and DCM. Complex I NADH-ubiquinone oxidoreductase 75 kDa subunit (NDUS1) was reduced only in samples from patients with CCC. Creatine kinase M (KCRM) and mitochondrial sarcomeric creatine kinase (KCRS), involved in the translocation of the ATP from the mitochondria to myofibrils, were found to be decreased in samples from patients with CCC and the other cardiomyopathies (**Table 4** and **Supplemental Figure S7D**).

**Figure 5:**
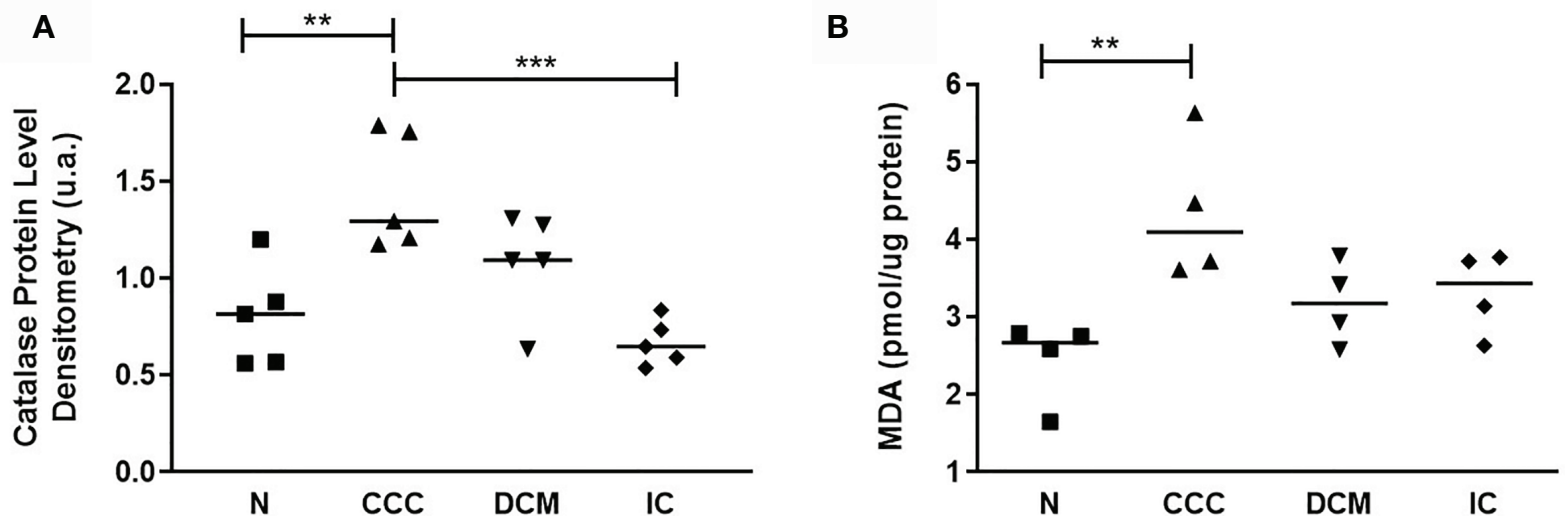
Analysis of antioxidant enzyme Catalase and lipid peroxidation status. **(A)** Catalase protein levels measured by immunoblotting; the densitometric values were normalized by the total protein for each sample (One-Way ANOVA p = 0.0008). **(B)** Malondialdehyde (MDA) production, measured by the thiobarbituric acid reactive substances (TBARS) assay (One-Way ANOVA p = 0.0113). The horizontal lines show statistically significant changes between groups by the Tukey-Kramer test: **p < 0.01; ***p < 0.001.

Regarding the stress response related proteins, we found 14 differentially expressed proteins in at least one of the patient groups, including chaperone, redox homeostasis and apoptosis-related proteins. The antioxidant peroxiredoxins (PRDX2, PRDX3 and PRDX6) displayed reduced expression in CCC and DCM. Catalase (CATA) was upregulated only in CCC patients; immunoblotting analysis showed that the total catalase protein level was increased as compared to the other IC samples and the control group (**Figure 5A**); protein disulfide-isomerases PDIA1 and PDIA3 were upregulated in CCC and DCM. Multiple chaperones were upregulated in CCC and DCM. Other proteins involved in apoptosis, such as TRAF3, which mediates pathological hypertrophy and apoptosis, was upregulated in the three cardiomyopathies. Tumor necrosis factor receptor superfamily member 10B (TR10B) - a receptor for the cytotoxic ligand TRAIL, and the antiapoptotic protein annexin A5 (ANXA5) showed increased expression CCC and DCM, while antiapoptotic heat shock 27 kDa protein (HSPB1), displayed decreased expression only in CCC.

Based on proteomic, immunoblotting and real time PCR data, the fatty acid beta-oxidation pathway seems to be significantly impaired in CCC, which could result in fatty acid accumulation. The reduction of peroxiredoxins - shared with DCM - and the increase in catalase - exclusively in CCC - is consistent with increased oxidative stress in CCC myocardium (**Supplemental Figure S9**). The increased levels of malonaldehyde (MDA), the major lipid oxidation product, were observed only in CCC, which indicates lipid peroxidation suggestive of lipotoxicity (**Figure 5B**).

We identified 20 differentially expressed proteins belonging to the structural and contractile protein group (**Table 4** and **Supplemental Figure S8**). From these, 5 proteins are exclusively modulated in CCC samples (e.g. actin-like protein 3 – ARP3 - and tropomyosin-1 alpha chain – TPM1, with increased and decreased levels, respectively). CCC and DCM samples shared 7 differentially expressed proteins, while CCC and IC shared only one differentially expressed protein. Among the proteins differentially expressed shared by CCC, DCM and IC samples (7 proteins) are Desmin (DESM), Gelsolin precursor (GELS), Lumican (LUM) and Troponin I (TNNI3), identified in spots with increased levels, and Alpha-cardiac actin (ACTC), LIM domain-binding protein 3 (LDB3) and Myosin-7 (MYH7), identified in spots with decreased levels, a pattern consistent with the fetal gene expression profile.

Among proteins that play a role or are regulated by components of the immune system, galectin-3 (LEG3) and proteasome activator complex subunit 1 (PSME1), upregulated by the inflammatory cytokines IFN-gamma and TNF-alpha, show increased levels exclusively in samples from patients with CCC. We also found B-cell related proteins to be upregulated in CCC, like tyrosine-protein kinase BLK (BLK), which plays an important role in the surface immunoglobulin signaling pathway and was also found with increased expression only in CCC samples. Likewise, we found increased protein levels of immunoglobulin (Ig gamma-1 chain C region - IGHG1) in the samples from patients with CCC, DCM and IC when compared to samples from individuals without cardiomyopathies. However, CCC samples showed the highest levels as compared to samples from the other patient groups.

**Figure 6:**
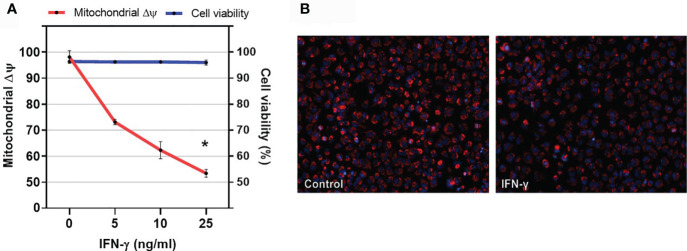
Effect of IFN-gamma on cardiomyocyte mitochondrial membrane potential. **(A)** Human cardiomyocytes AC16 were stimulated with 5, 10 or 25ng/ml of IFN-gamma for 48 hours. Then, cells were stained using 1µM TMRM, 400nM of mitotracker DeepRed, 500ng/ml of PI and 1µM of Hoechst 33342 and micrographs were captured in ImageXpress Micro XLS Widefield High-Content Analysis system at 100x magnification. Fluorescence colocalization of TMRE and mitotracker deepred in live cells (PI negative) was used to calculate mitochondrial ΔΨ. Data are reported as the ratio to not-treated cells. Cell viability is the ratio of the number of live cells (PI-negative) and total cells (PI-negative plus PI-positive cells) x 100. n = 3. *p < 0.05. **(B)** Representative fluorescence microscopy (100x) showing decrease in TMRE fluorescence after incubation with IFN-gamma for 48h.

To investigate the role of IFN-gamma and TNF-alpha on cardiomyocyte mitochondrial function, we stimulated the human adult cardiomyocyte cell line AC16 with several concentrations of IFN-gamma and measured the mitochondrial ΔΨm with supravital fluorescence microscopy. We observed that IFN-gamma impaired the ΔΨm of AC-16 48h after stimulation (**Figure 6**).

## Discussion

The proteomic analysis of myocardial tissue revealed that CCC, DCM and IC display a distinct global protein expression profile. Pathway analysis disclosed enrichment in mitochondrial dysfunction, cardiac hypertrophy and fibrosis in the three disease groups. Pathway analysis of proteins differentially expressed in CCC also showed selective enrichment in the CCC group for pathways involved in the fatty acid metabolism and decreased transmembrane potential of mitochondria. CCC patients’ myocardium displayed reduced expression of 22 mitochondrial proteins belonging to energy metabolism pathways, as compared to control samples, while IDC and IC displayed 15 and 3, respectively. Significantly, 6 lipid beta-oxidation enzymes were reduced in CCC, while only 2 of them were downregulated in DCM and 1 in IC. To our knowledge, this is the first report on the differential myocardial protein expression profile of multiple cardiomyopathies, including Chagas disease cardiomyopathy. In addition, we found increased levels of malonaldehyde, a sign of oxidative stress and a toxic product of lipid peroxidation, only in CCC samples. Finally, we observed that IFN-gamma treatment of the human cardiomyocyte cell line AC16 induces a dose-dependent reduction of mitochondrial transmembrane potential, providing a possible clue to the inflammatory origin of mitochondrial dysfunction in CCC.

Functional analysis of differentially expressed proteins shows that myocardial samples from patients with CCC display a substantial reduction in levels of proteins involved in several pathways of mitochondrial energy metabolism, particularly in the beta-oxidation pathway. The finding that several enzymes involved in beta-oxidation are decreased in CCC, while only one is also reduced in DCM suggests that this pathway may be selectively reduced in CCC patients. The finding that the mRNA levels of ACADVL were also decreased in samples from patients with CCC suggests that enzyme levels may be transcriptionally regulated. Very long chain fatty acid dehydrogenase (ACADVL) has activity mainly toward CoA-esters of fatty acids with 16–24 carbons in length (35). In addition, ACADVL catalyzes the major part of palmitoyl-CoA dehydrogenation in many human tissues and cultured cells (36), indicating its central role in the catabolism of long-chain fatty acids. This is clearly reflected by the severe clinical symptoms caused by ACADVL deficiency, such as a high incidence of cardiomyopathy in childhood (37). Patients with ACADVL deficiency may present hypertrophy and dilated cardiomyopathy (38). In addition to the impaired degradation of very long and medium acyl chains, found mainly in CCC samples, we have also observed that other beta-oxidation enzymes involved in degradation of unsaturated fatty acids such as 2,4-dienoyl-CoA reductase (DECR) and 3,2-trans-enoyl-CoA isomerase (D3D2) showed reduced expression in CCC. This reduced expression is shared with DCM and IC, respectively. Activation of NF-κB is most likely to occur in heart tissue from CCC patients, due to the systemic and local expression of IFN-gamma, TNF-alpha and other proinflammatory cytokines (11, 18, 19, 39). Studies showed that activation of nuclear factor NF-κB during cardiac hypertrophy decreases the activity of the protein PPAR (peroxisome proliferator-activated receptor) beta/gamma, leading to a decrease in fatty acid oxidation (40). Furthermore, malate Dehydrogenase (MDHM), exclusively reduced in CCC, is part of the tricarboxylic acid (TCA) cycle. We also found decreased levels of other TCA cycle enzymes, and increased expression of a single TCA enzyme, that were shared with other cardiomyopathies. Studies from our group showed that the gene SERCA Ca^++^ ATPase of sarcoplasmic reticulum (SERCA2), involved in cardiac homeostasis of Ca^++^ was found with reduced expression in the myocardium of patients with CCC (15), possibly indicating a reduction in calcium signaling and excitation/contraction coupling. Studies show that several enzymes of the TCA cycle can be activated by Ca^++^. Disturbed Ca^++^ homeostasis could lead to the inactivation of dehydrogenases of the TCA cycle by calcium, resulting in a decreased concentration of NADH and hence a reduction in ATP production (41). The finding that several components of complexes I, II, III, and V from the oxidative phosphorylation process showed reduced expression in CCC samples may also contribute to the impairment of ATP production (42). We have also observed reduced expression of several components of the creatine kinase complex, responsible for the translocation of ATP from mitochondria to the myofibrils. Creatine kinase M (KCRM) and mitochondrial creatine kinase (KCRS) showed reduced expression in samples from both CCC and other cardiomyopathies, as previously reported for heart failure (43–45); this finding corroborates previous results from our group (23). Our previous studies also showed that total protein levels (as detected by Immunobloting) and enzymatic activity of KCRM are significantly decreased in CCC myocardium samples as compared to samples from individuals without cardiomyopathies, as well as from DCM and IC patients. Reduction of protein levels of enzymes involved in multiple pathways that lead to ATP production is consistent with an energy deficit in CCC heart tissue. This reduced state of ATP production has been corroborated *in vivo* myocardial ATP flux evaluations (24, 46).

Our finding of increased protein levels of catalase (CATA) only in CCC myocardial samples, together with the reduction in several peroxyredoxins including PRDX6, an IFN-gamma regulated gene, suggests a significant disturbance in the antioxidant system. It is possible that the increased protein level of catalase found in CCC myocardium samples is secondary to a compensatory mechanism for oxidative stress, which could be more intense in CCC than in DCM or IC. Indeed, IFN-gamma, a cytokine that is highly expressed in CCC heart tissue (17, 18, 20) induces expression of NOX2 and increases oxidative stress (47). Increased oxidative stress is intimately involved in the pathogenesis of heart failure. Isocitrate dehydrogenase [NADP] (IDHP), which was found with reduced expression in samples of patients with CCC and DCM, is responsible for the production of NADPH. Indeed, the decrease in the levels of IDHP is related to increased reactive oxygen species, DNA fragmentation, lipid peroxidation, and mitochondrial damage with significant reduction in ATP levels (48). The reduced protein levels of beta-oxidation enzymes involved in degradation of fatty acids may lead to an accumulation of lipid substrates in heart tissue. Given the deficiency in the antioxidant defense system in CCC, it is possible to hypothesize that oxidant conditions prevail in CCC heart tissue, in line with results in experimental *T. cruzi* infection (49–52) which together with our finding of reduced beta- oxidation enzymes could lead to lipid peroxidation.

Our data suggest that CCC myocardium displays signs of reduced mitochondrial activity and energy production. Garg et al. were the first to suggest that myocardial mitochondrial dysfunction and oxidative stress in the pathogenesis of murine models of CCC (reviewed in (53)). Decreased mitochondrial rRNA (15), rDNA (54) and *in vivo* ATP production (24) were observed in CCC myocardium. The IFN-gamma-producing T-cell rich inflammatory profile is a major difference between CCC and DCM; indeed, IFN-gamma is the most highly expressed cytokine in CCC heart tissue (14, 17–19, 55) and the top upstream gene regulator upon pathways analysis in CCC myocardium (20). It is known that IFN-gamma has multiple deleterious effects on cardiomyocyte mitochondria. It induces TNF-alpha and potentiates TNF-alpha-mediated NF-kB signaling, leading to NOS2 production of reactive nitrogen species (RNS) (56, 57). This has been reported to cause mitochondrial fragmentation, disturbance in mitochondrial membrane potential, and reduction of ATP production (58). In addition, IFN-gamma was shown to reduced fatty acid beta-oxidation (59) and oxidative phosphorylation (60), inducing a shift towards glycolysis (61). These factors are probably involved in IFN-gamma-induced cardiomyocyte dysfunction and apoptosis (62). We hypothesize that many of the mitochondrial energy metabolism changes observed in CCC are locally induced by the high levels of IFN-gamma in CCC myocardium.

IFN-gamma induced proteins like Galectin 3 (LEG3) and Proteasome activator complex subunit 1 - PSME1), found to be upregulated in CCC myocardial tissue, may play a role in the pathology of heart failure and cardiac remodeling; with Galectin-3 showing a stimulatory effect on macrophage migration, fibroblast proliferation and development of fibrosis. Increased levels of Galectin-3 were also found in the hearts of transgenic mice with increased expression of IFN-gamma (63) and mice chronically infected by *T. cruzi*, and shows increased expression in areas of inflammation in CCC myocardium (64). Moreover, mice genetically deficient in Galectin-3 display less myocardial fibrosis at chronic infection (65). Significantly, plasma Galectin-3 levels are a prognostic factor for heart failure (66) and were associated with long-term mortality in CCC (67). PSME1 is the alpha subunit of the immunoproteasome PA28 complex. Significantly, it has been reported that genes encoding the IFN-gamma-induced immunoproteasome subunits display increased expression in CCC heart tissue and the myocardium of *T. cruzi*-infected mice (68). The reduced protein levels of AKT2 (Rac-beta serine/threonine-protein kinase) - which has a role in apoptosis inhibition, through the inhibition of JNK and p38 activation (69) - and the increased protein levels of TR10B (Tumor necrosis factor receptor superfamily member 10B) - a receptor for the apoptosis-inducing ligand TRAIL (TNF-related apoptosis-inducing ligand) - could both facilitate apoptosis (70) and increase susceptibility to oxidative stress.

The involvement of sarcomeric/structural proteins in the pathogenesis of cardiomyopathy is a consequence of the cardiac remodeling and is part of the hypertrophy/embryonic gene expression signature. The finding of 4 structural proteins exclusively down-modulated in CCC may suggest the remodeling is even more intense in CCC than in other cardiomyopathies.

Our study has limitations. The number of samples for the proteomic studies was limited and control samples were significantly younger than patient’s samples. This is unavoidable as organ donors tend to be much younger than organ recipients. It thus follows that some of the protein expression changes shared by all cardiomyopathy groups in comparison to organ donor controls could be due to age alone. However, most of the energy metabolism enzyme changes were not shared among the 3 patient groups as compared to organ donor controls.

**Figure 7:**
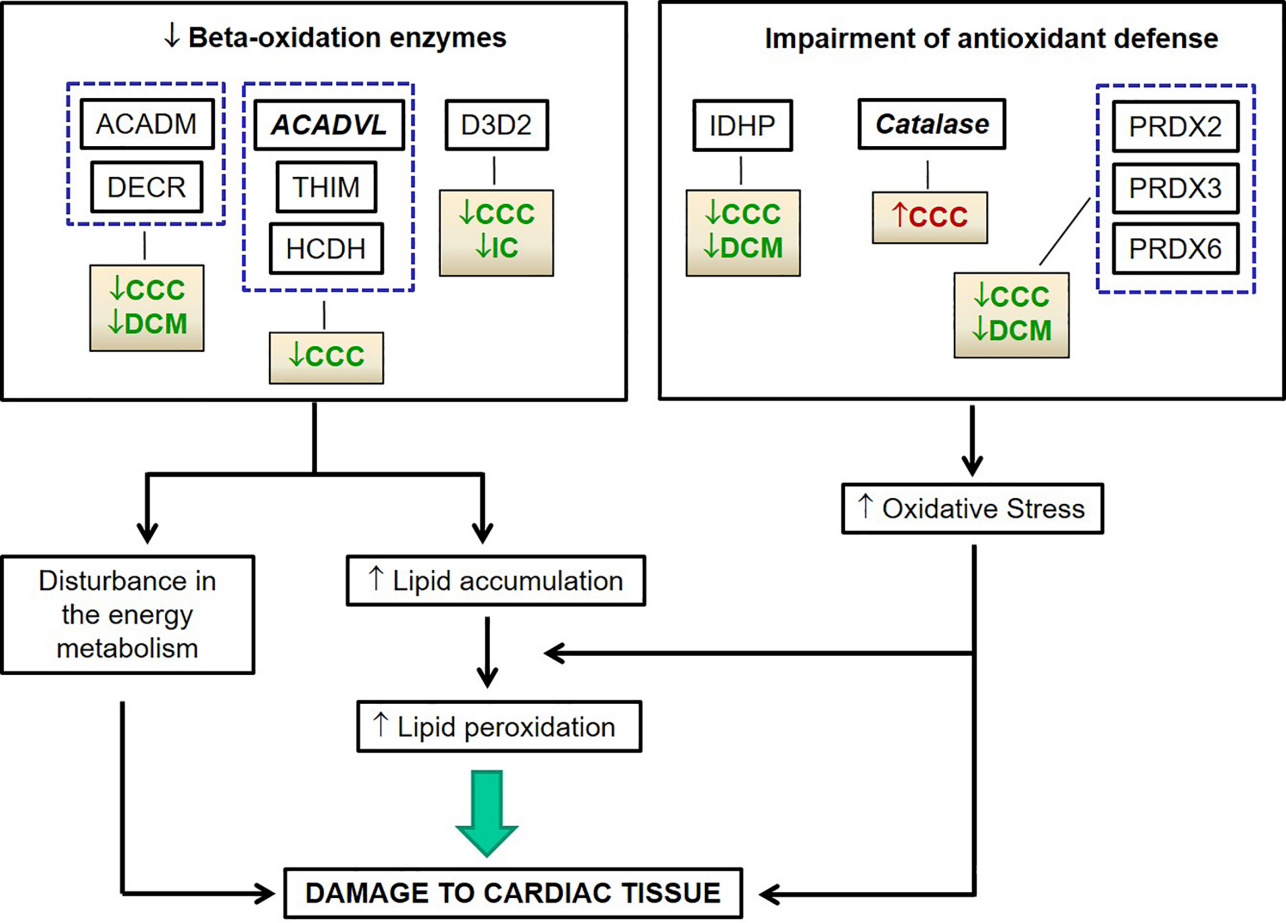
Hypothetical chain of events subsequent to reduced beta-oxidation and oxidative stress related proteins in CCC myocardium leading to cardiac damage.

In summary, proteomic analysis disclosed profound disturbances in several pathways of energy metabolism – including beta-oxidation, tricarboxylic acid cycle, oxidative phosphorylation, and creatine kinases, suggesting a major reduction of mitochondrial energy metabolism which is more significant in CCC than other end-stage dilated cardiomyopathies. In addition, disturbances in the local antioxidant defense system corroborate findings in experimental models and may be related to the active inflammatory process. Oxidative stress-dependent peroxidation of hypothetically accumulated fatty acids as a consequence of decreased levels of beta-oxidation enzymes in CCC might lead to the increased levels of highly toxic molecules such as malonaldehyde (**Figure 7**). Given the high levels of IFN-gamma in CCC heart tissue, we hypothesize that many mitochondrial changes and oxidative stress observed in CCC are due to the continued high expression of this cytokine. This hypothesis is investigated in the back-to-back submitted paper. These factors may play a role in the increased aggressiveness of CCC as compared to other dilated cardiomyopathies and places mitochondrial function as a therapeutic target in Chagas disease. Although anti-cytokine treatment is not an option in Chagas disease due to the risk of reactivation of infection, downstream pathways that promote mitochondrial function are likely therapeutic targets. Indeed, fenofibrate, a PPAR- agonist capable to induce fatty acid beta-oxidation, was able to restore cardiac function in a murine model of Chagas disease (71). Likewise, treatment of chronically *T. cruzi*-infected mice with mitochondria-targeting SIRT1 and/or AMPK agonists SRT1720, resveratrol and metformin reduced myocardial NF-κB transcriptional activity, inflammation and oxidative stress, resulting in beneficial results for restoration of cardiac function (50, 51). Therapy targeting mitochondrial function and energy imbalance should thus in principle be beneficial to restore cardiac function in CCC and other IFN-gamma-dependent inflammatory heart diseases, like viral myocarditis and inflammatory cardiomyopathy of other etiologies and age-related myocardial inflammation and functional decline (72), myocardial infarction (73) and anthracycline antitumor agent cardiotoxicity.

## Data Availability Statement

The datasets presented in this study can be found in online repositories. The names of the repository/repositories and accession number(s) can be found in the article/**Supplementary Material**.

## Ethics Statement

The protocol was also approved by the INSERM Internal Review Board and the Brazilian National Ethics in Research Commission (CONEP). The patients/participants provided their written informed consent to participate in this study.

## Author Contributions

Study design: PT, EC-N, and JK. Phenotype characterization: RS, LB, EB, AF, NS, and PP. Experimental analysis: PT, AD, HL, EN, JS, DL, SB, and AK. Statistical analysis: PT, JS, and EC-N. Manuscript preparation: PT, EC-N, JS, and CC. All authors contributed to the article and approved the submitted version.

## Funding

This research was supported by the Brazilian Council for Scientific and Technological Development - CNPq and the São Paulo State Research Funding Agency - FAPESP (grant 2013/50302-3). PT was a recipient of a São Paulo State Research Funding Agency - FAPESP fellowship. EC-N and JK are recipients of Brazilian Council for Scientific and Technological Development - CNPq productivity awards 1A. JPSN received a fellowship from Institute MarMaRa. Proteomic analysis was partially performed at and funded by F. Hoffmann-La Roche, Basel, Switzerland. This work was supported by the Institut National de la Santé et de la Recherche Médicale (INSERM); the Aix-Marseille University; the French Agency for Research (Agence Nationale de la Recherche-ANR (grant numbers: “Br-Fr-Chagas”, “landscardio”). This project has received funding from the Excellence Initiative of Aix-Marseille University - A*Midex a French “Investissements d’Avenir programme”- Institute MarMaRa AMX-19-IET-007. The funders had no role in study design, data collection and analysis, decision to publish, or preparation of the manuscript.

## Author Disclaimer

The content of this publication, as well the opinions and views expressed herein, was part of a scientific collaboration between the authors and should not be considered, in whole or in parts, as being statements by the company Hoffmann-La Roche.

## Conflict of Interest

PT, AD, and EN are current employees of F. Hoffmann-La Roche Ltd and may own company stock.

The remaining authors declare that the research was conducted in the absence of any commercial or financial relationships that could be construed as a potential conflict of interest.

## Publisher’s Note

All claims expressed in this article are solely those of the authors and do not necessarily represent those of their affiliated organizations, or those of the publisher, the editors and the reviewers. Any product that may be evaluated in this article, or claim that may be made by its manufacturer, is not guaranteed or endorsed by the publisher.

## Supplementary Material

Supplementary Figure 1 Scheme of the 2D-DIGE approach for the differential proteomic analysis. The approach relies on multiplexing, and simultaneous co-separation of multiple, fluorescently labeled samples, including a pooled internal standard on each gel (Scheme adapted from Ettan DIGE brochure – GE Healthcare).

Supplementary Figure 2 Representative images from two-dimensional electrophoresis (2D-DIGE) experiments. **(A)** Preparative 2-DE gel used to protein identification by mass spectrometry; **(B)** Representative 2-DE gel of left ventricle myocardial proteins with overlapping images; **(C)** Representative 2-DE gel of left ventricle myocardial proteins displaying each individual fluorescent images (Cy3: DCM, Cy5: CCC, Cy2: Pool).

Supplementary Figure 3 Volcano plots. The plots display the number of differentially expressed spots (p<0.01, t-test) in: **(A)** CCC samples, **(B)** DCM samples and **(C)** IC samples, as compared to individuals without cardiomyopathy. Total number of spots: 683 spots.

Supplementary Figure 4 Venn diagrams representing the occurrence of spots differentially expressed in common or unique relationships between groups of patients with cardiomyopathy when compared with subjects without cardiomyopathy. Number of spots with increased **(A)** or decreased **(B)** levels in samples from patients when compared with samples from subjects without cardiomyopathy.

Supplementary Figure 5 Protein Classification. **(A)** Cellular component classification and **(B)** biological process classification of proteins identified in the proteomic analysis.

Supplementary Figure 6 Protein and mRNA levels of DECR1 (2,4-dienoyl-CoA reductase 1) in myocardial tissue of CCC, DCM, IC and controls. **(A)** Densitometry measurement of DECR1 protein levels using immunoblot (One-Way ANOVA p = 0.0011). **(B)**. DECR1 mRNA levels assessed using real time RT-qPCR (One-Way ANOVA p = 0.0154). The horizontal lines show statistically significant changes between groups by the Tukey-Kramer test: *p < 0.05; ***p < 0.001.

Supplementary Figure 7 Cartoons depicting energy metabolism related proteins differentially expressed in CCC, DCM and IC myocardium. **(A)** Glycolysis, **(B)** Citric Acid Cycle, **(C)** Oxidative Phosphorylation, **(D)** Creatine Kinase System. Arrows indicate up-regulated (red) or down-regulated (green) expression as compared to control myocardial samples in the proteomic analysis.

Supplementary Figure 8 Cartoon depicting sarcomeric related proteins differentially expressed in CCC, DCM and IC myocardium. Arrows indicate up-regulated (red) or down-regulated (green) expression as compared to control myocardial samples in the proteomic analysis.

Supplementary Figure 9 Representative images from Immunoblotting analysis. **(A)** One-dimensional electrophoresis of the samples used for immunoblotting experiments. SDS-PAGE 12.5%, 30μg of proteins, gel stained by “Coomasie Blue Colloidal”. **(B)** Representative immunoblotting (Western-blotting) of ACADVL (Very long-chain specific acyl-CoA dehydrogenase), DERC (2,4-dienoyl-CoA reductase 1) and CATA (Catalase) used for densitometry analysis. Relative abundance of a specific protein across the lanes of the blot was given by the normalization to the total amount of protein in each lane.
