# Supplementary material for: Impairment of Multiple Mitochondrial Energy Metabolism Pathways in the Heart of Chagas Disease Cardiomyopathy Patients
Source: Front Immunol. 2021 Nov 12;12:755782. doi: 10.3389/fimmu.2021.755782 (PMC8633876; doi:10.3389/fimmu.2021.755782)
Supplement: Supplementary Figure 1 — Scheme of the 2D-DIGE approach for the differential proteomic analysis. The approach relies on multiplexing, and simultaneous co-separation of multiple, fluorescently labeled samples, including a pooled internal standard on each gel (Scheme adapted from Ettan DIGE brochure – GE Healthcare). [file DataSheet_1.pdf]

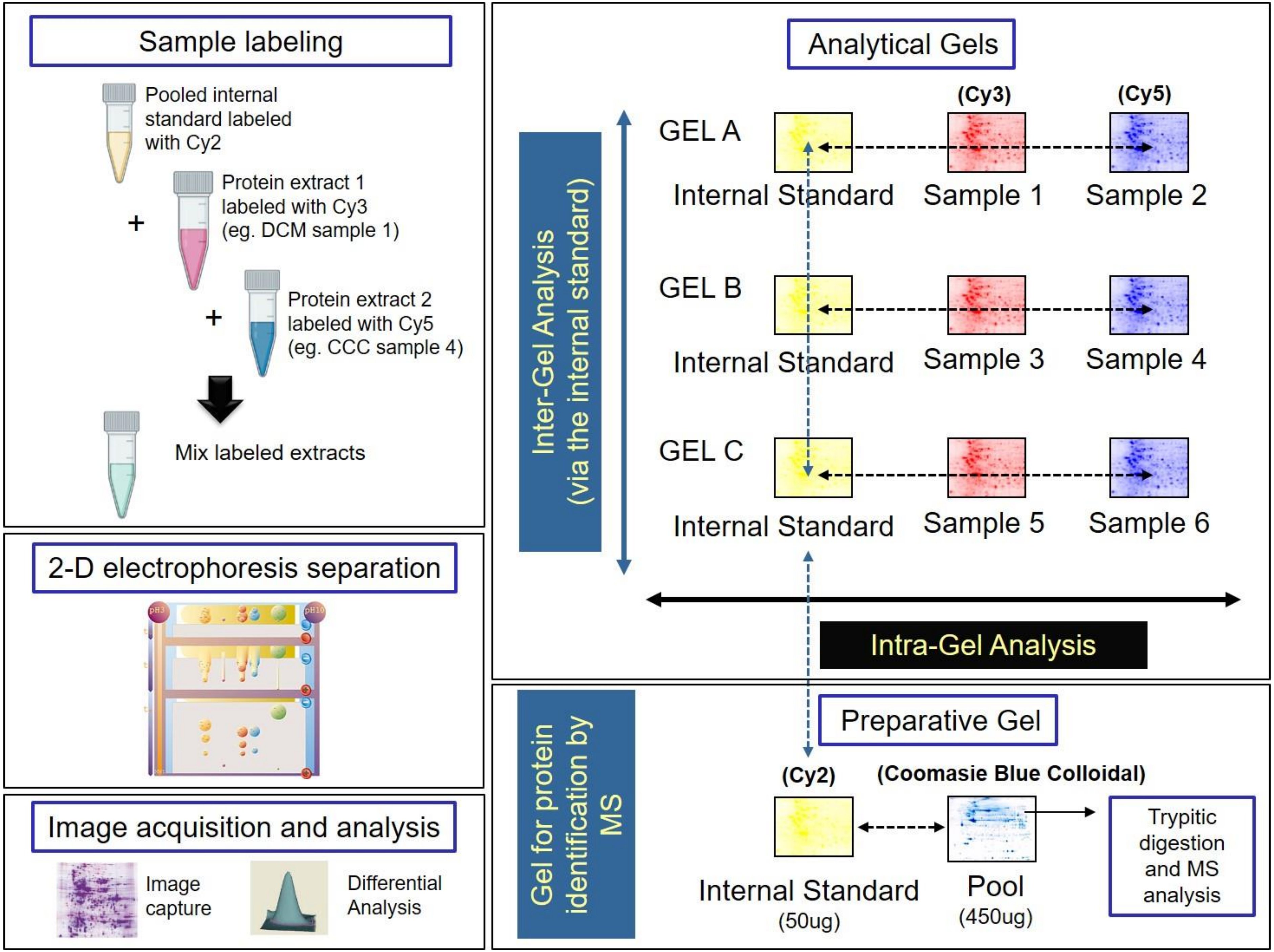

Supplemental Figure S1

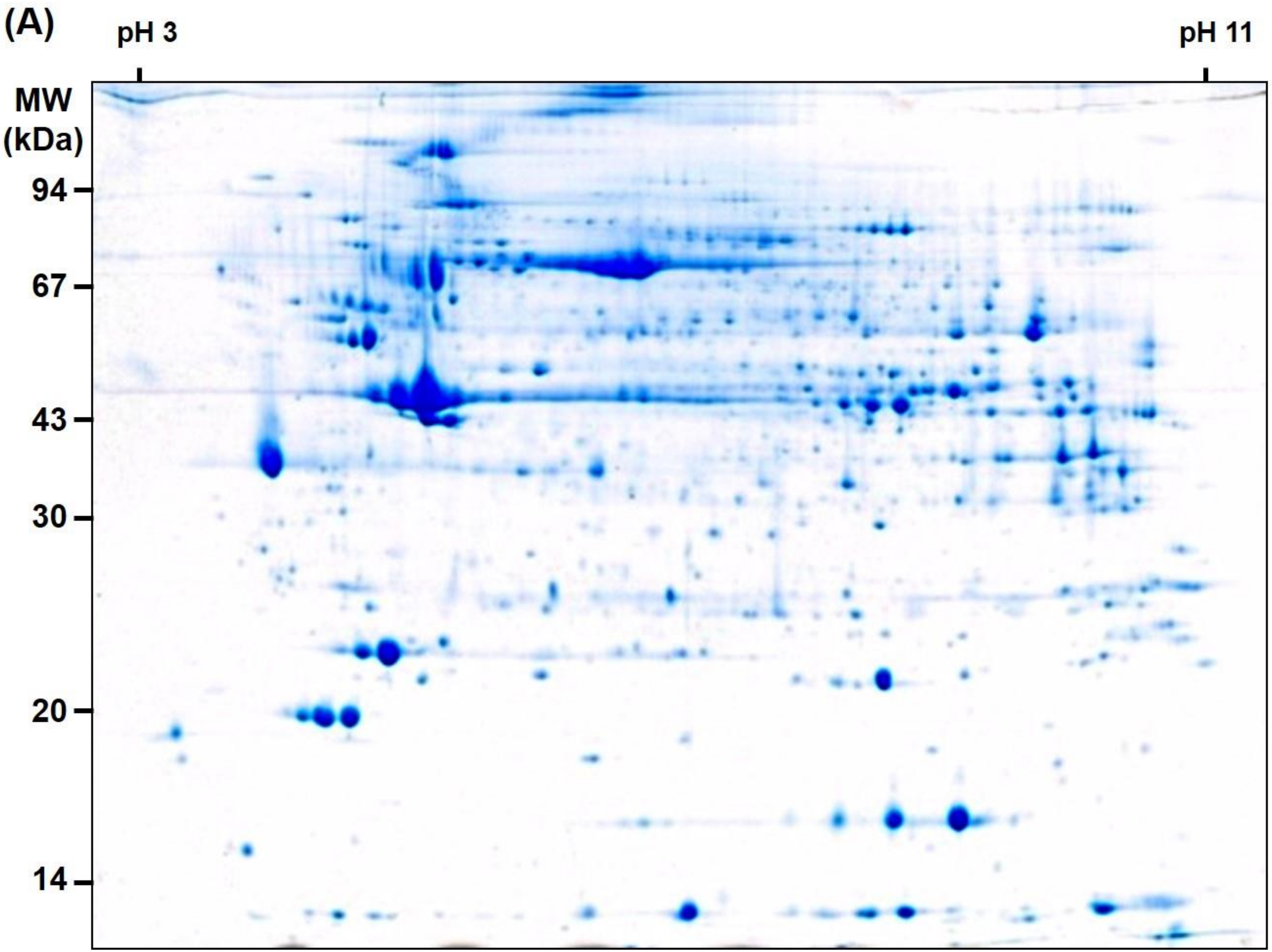

Supplemental Figure S2a

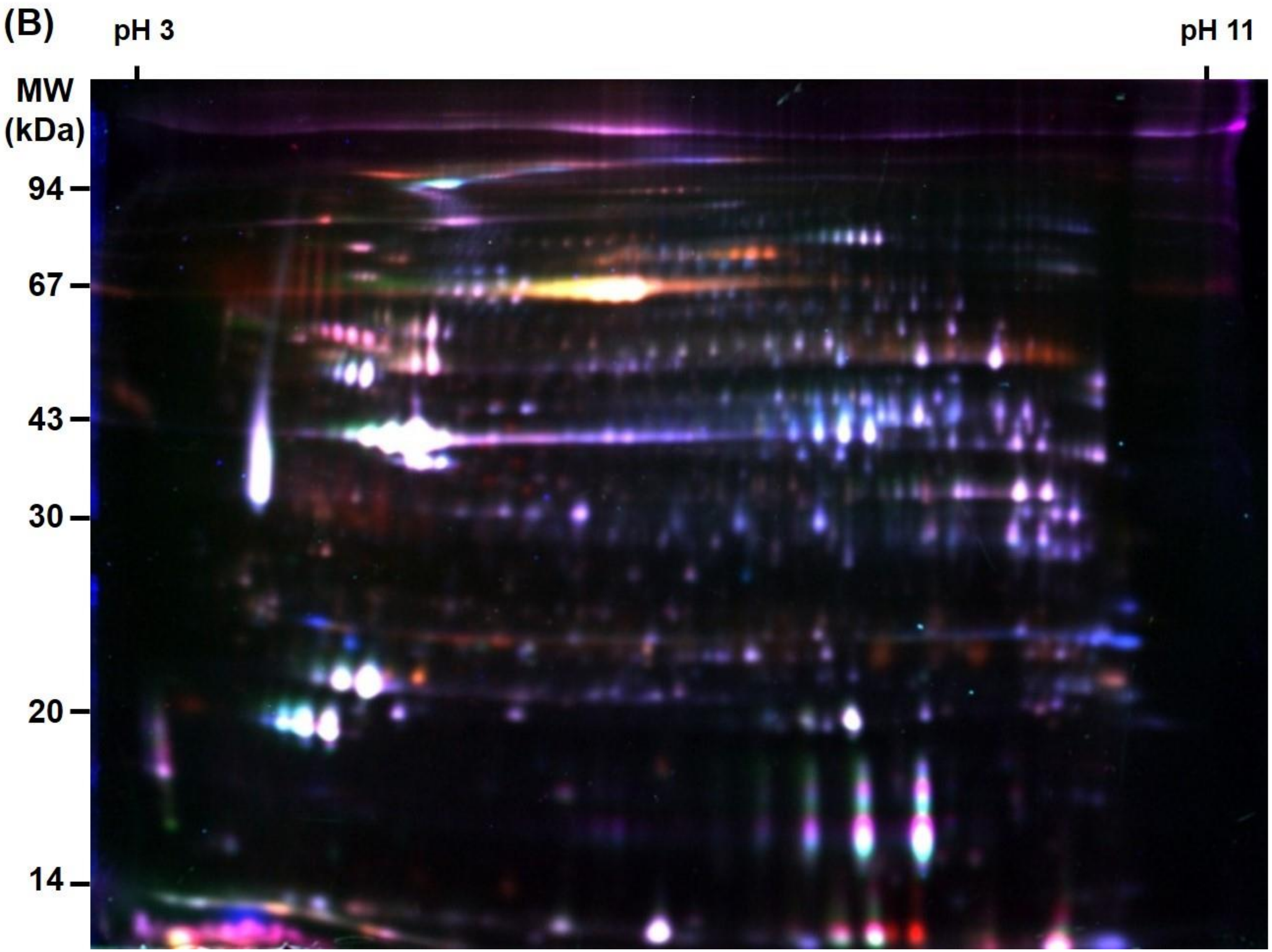

Supplemental Figure S2b

(C)

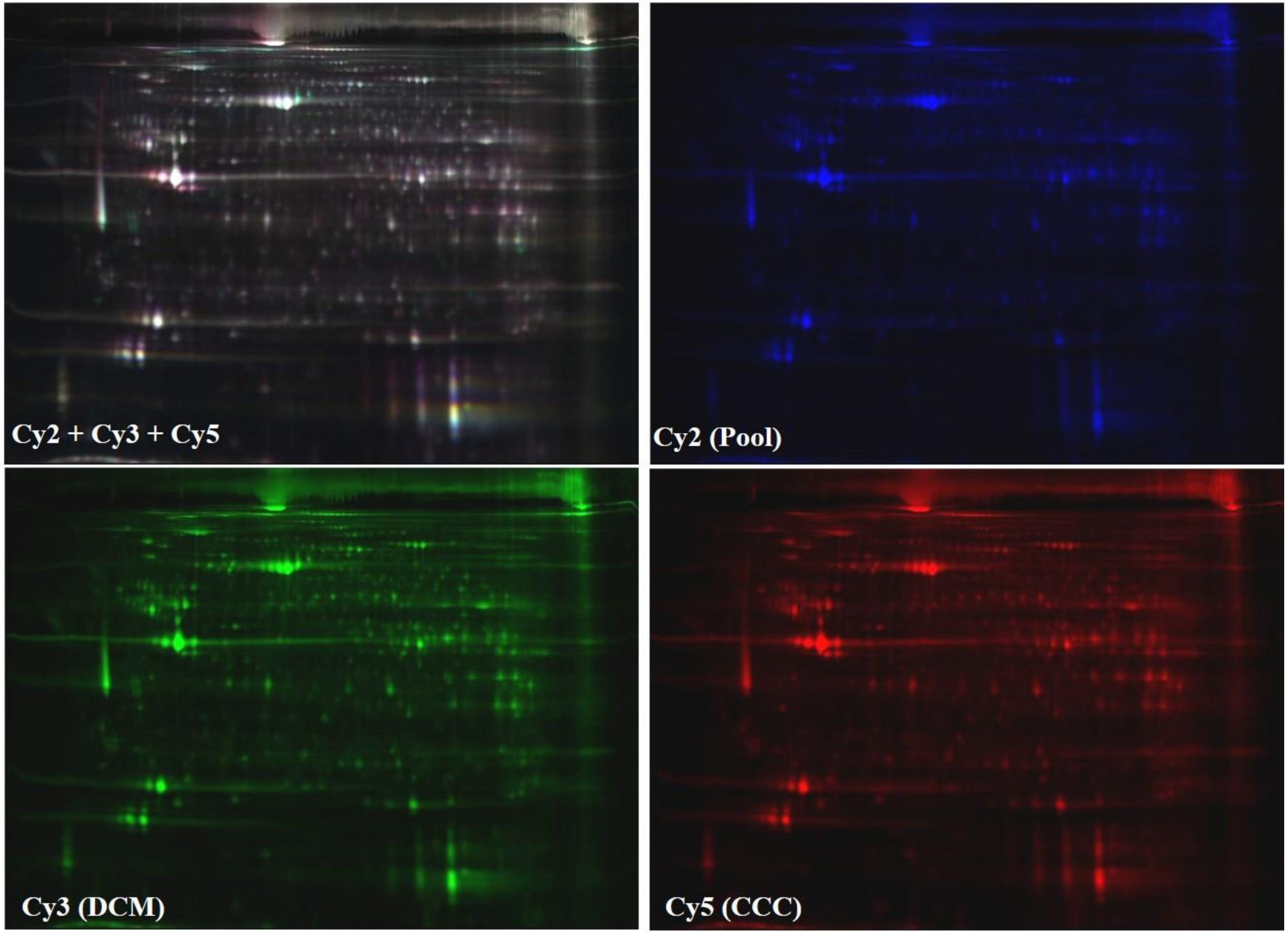

Supplemental Figure S2c

Supplemental Figure S3a

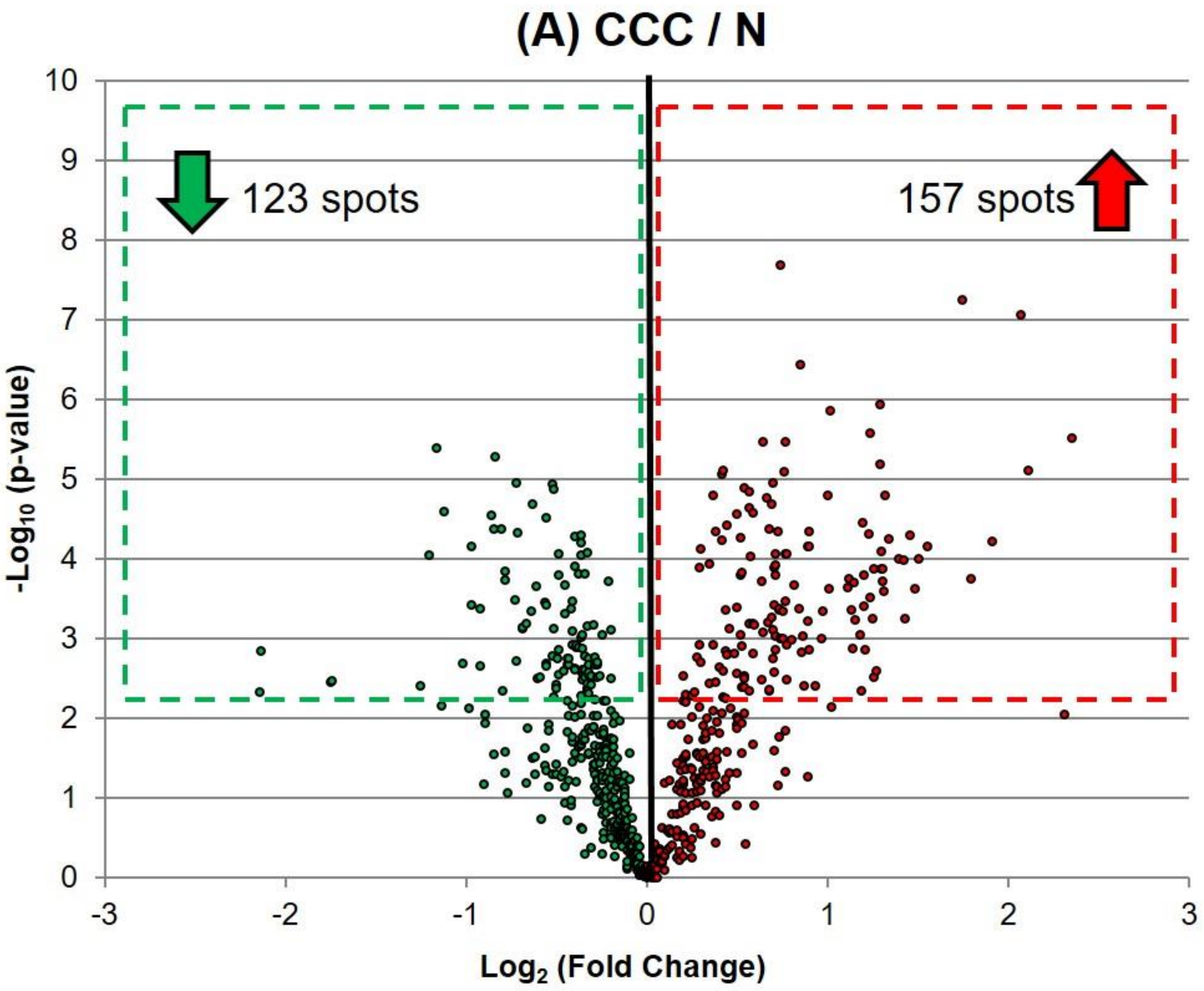

Supplemental Figure S3b

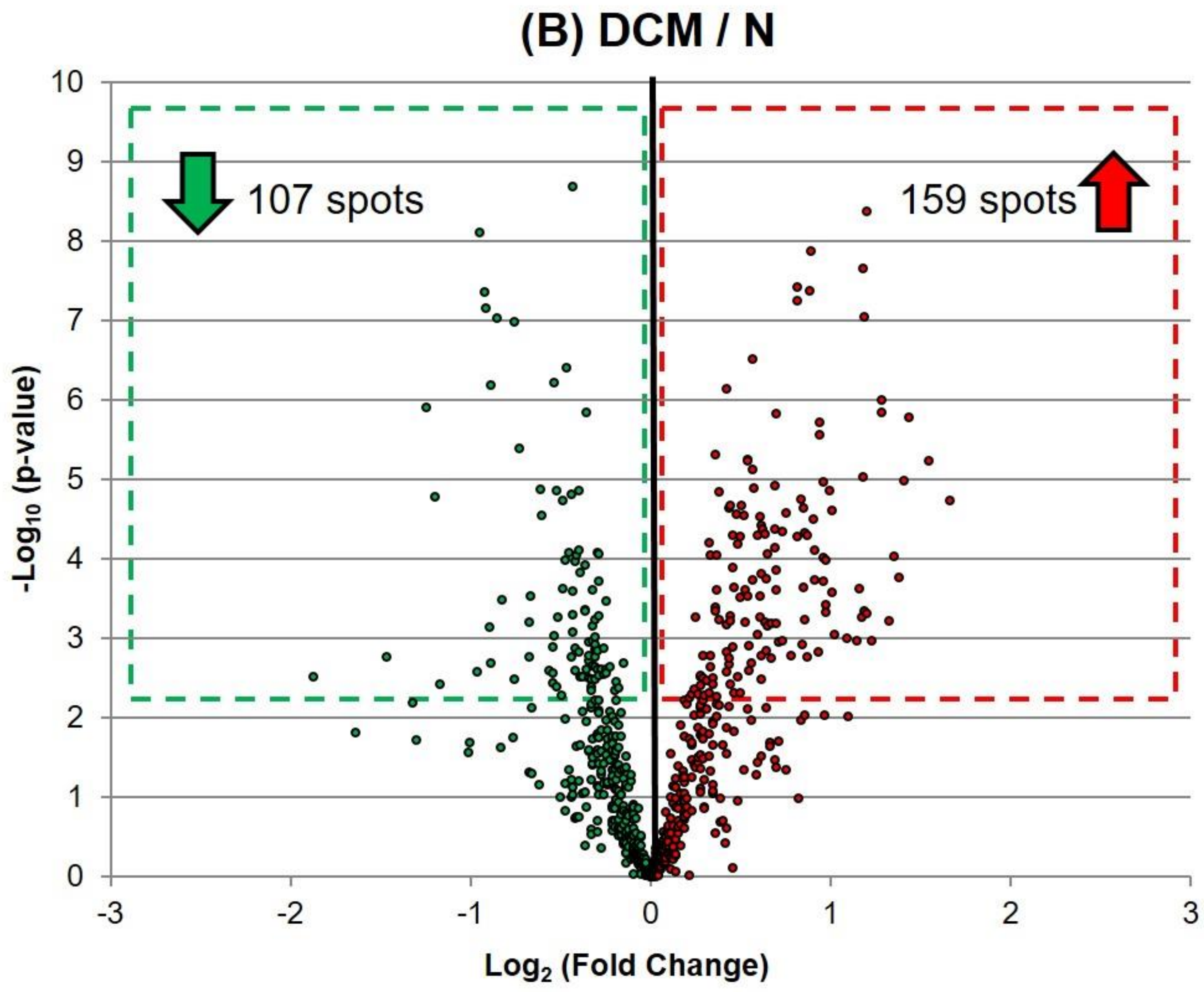

Supplemental Figure S3c

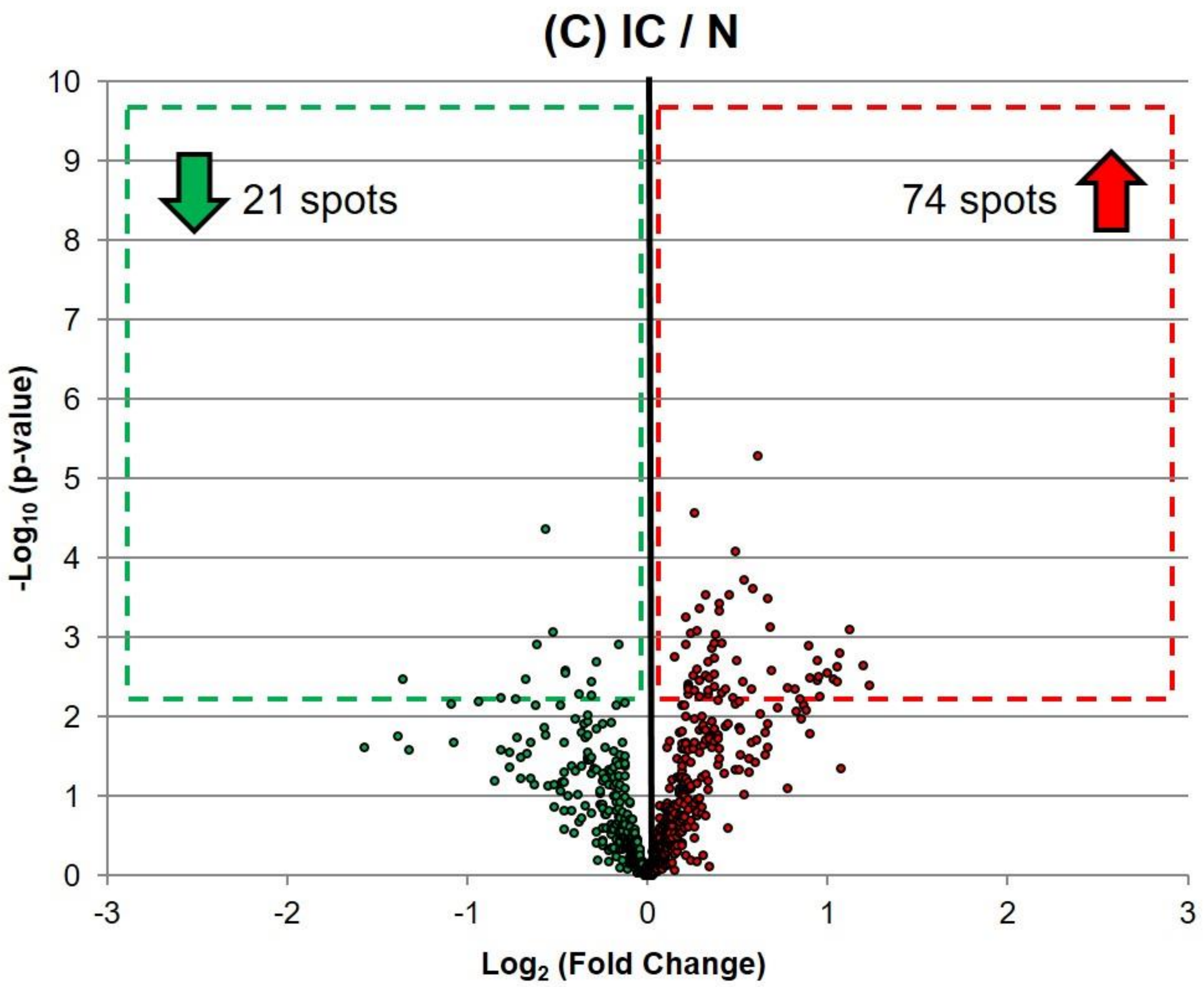

Supplemental
Figure S4

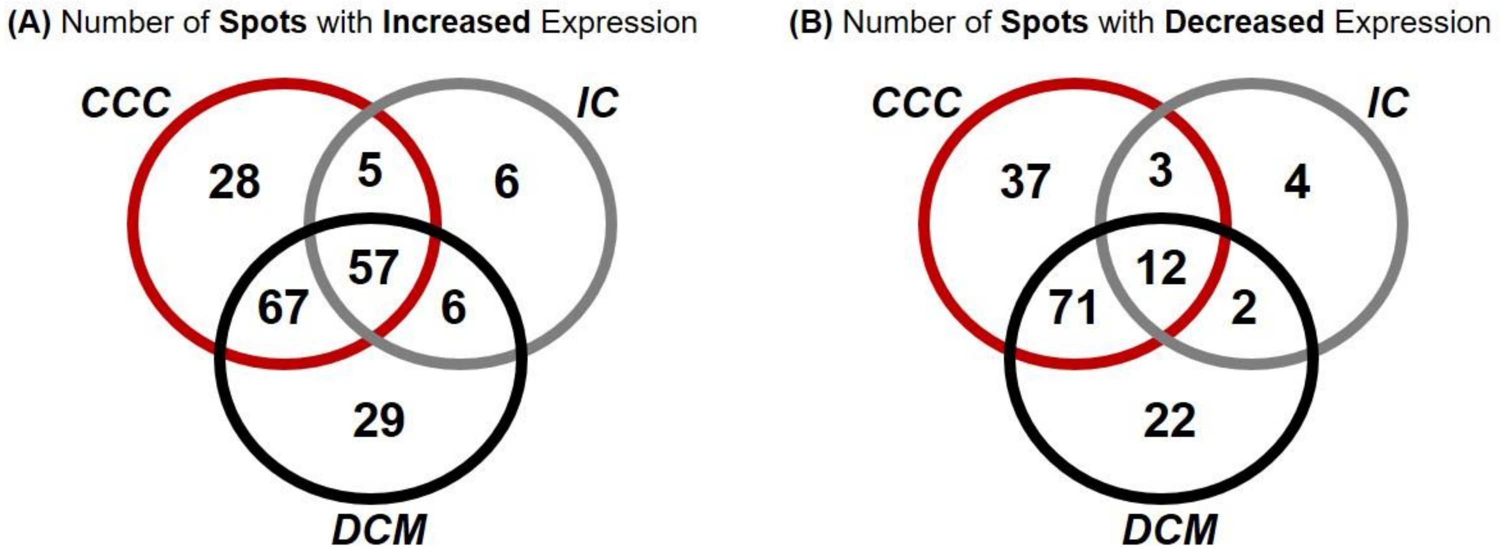

Supplemental Figure S5a

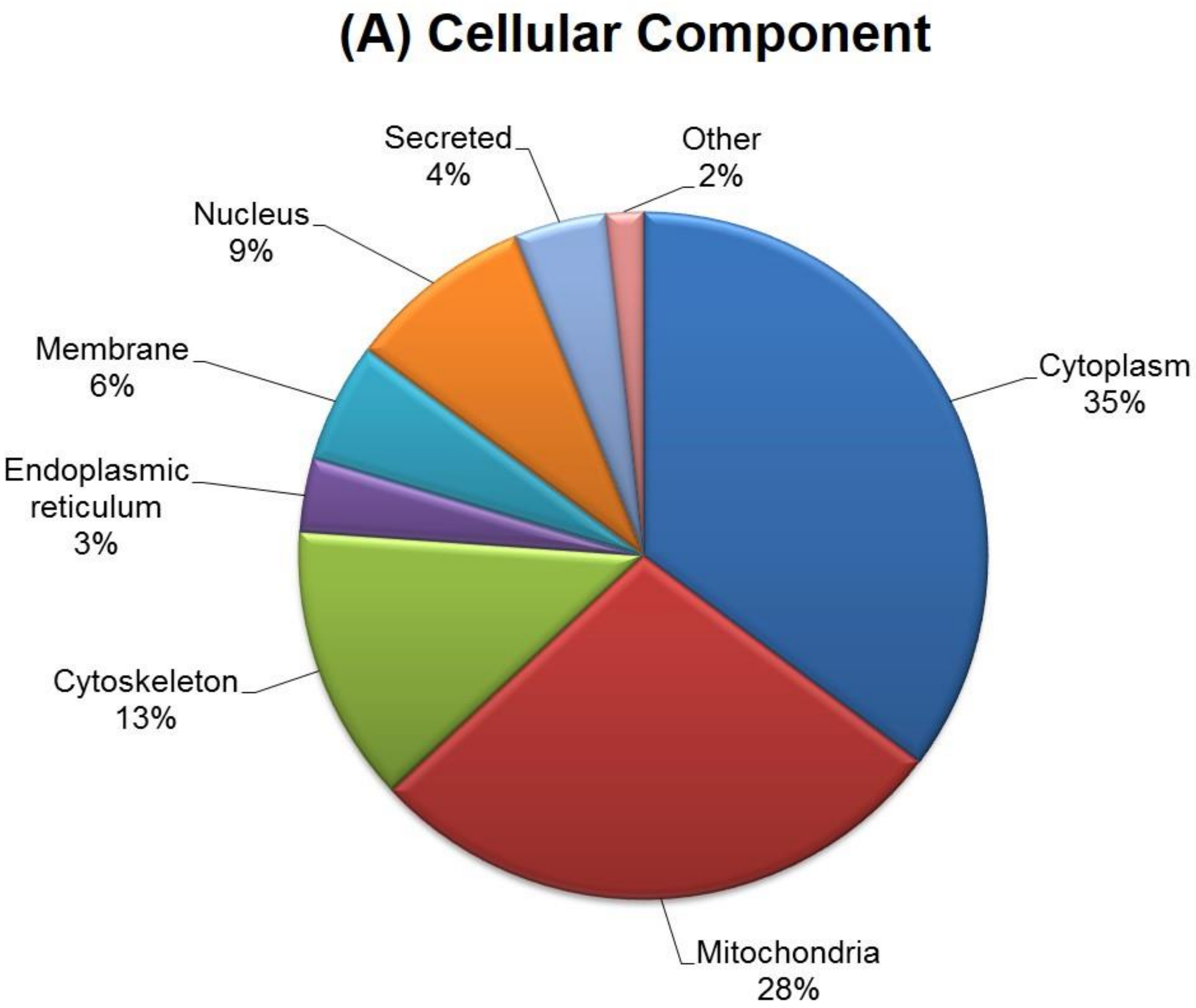

Supplemental Figure S5b

(B) Biological Process

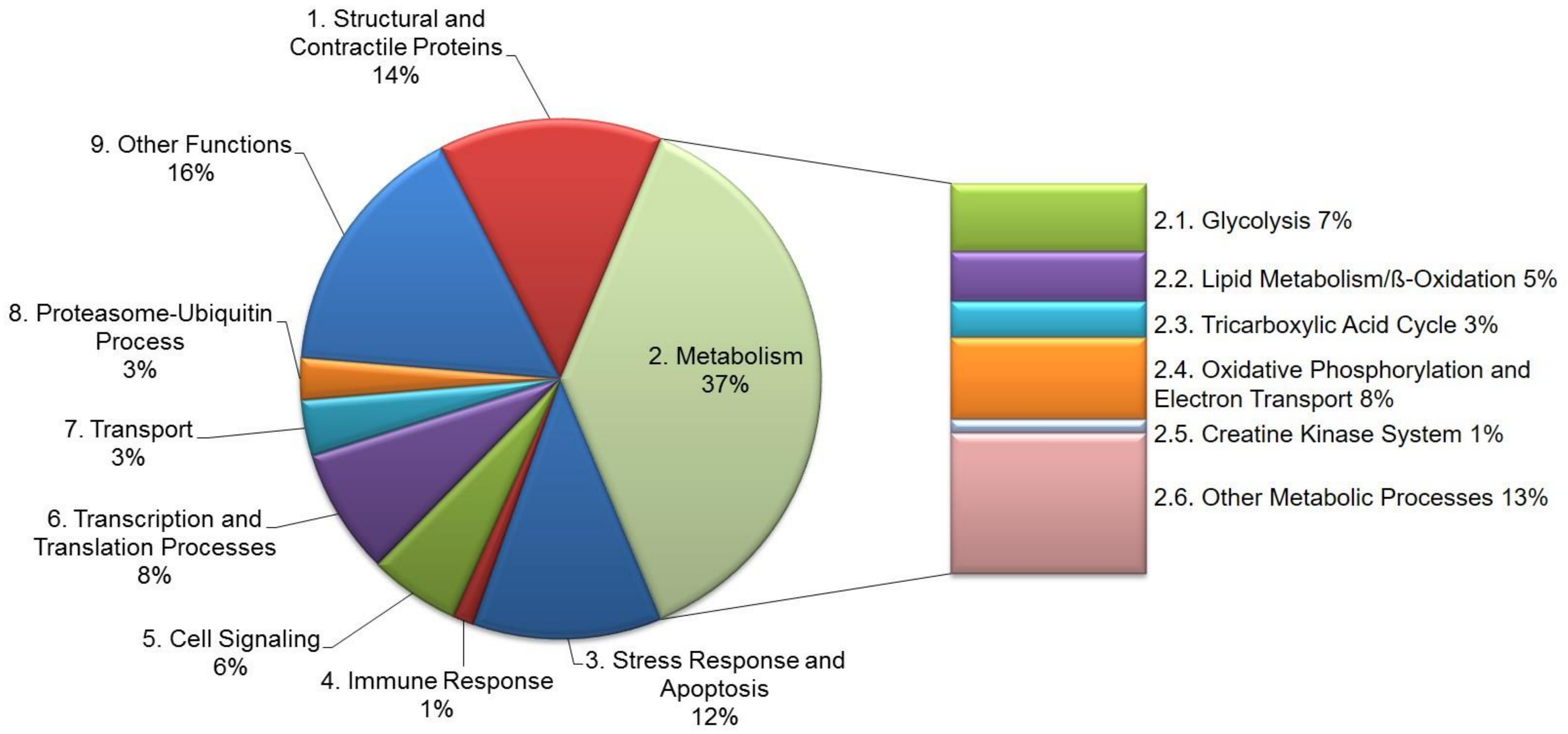

Supplemental Figure S6

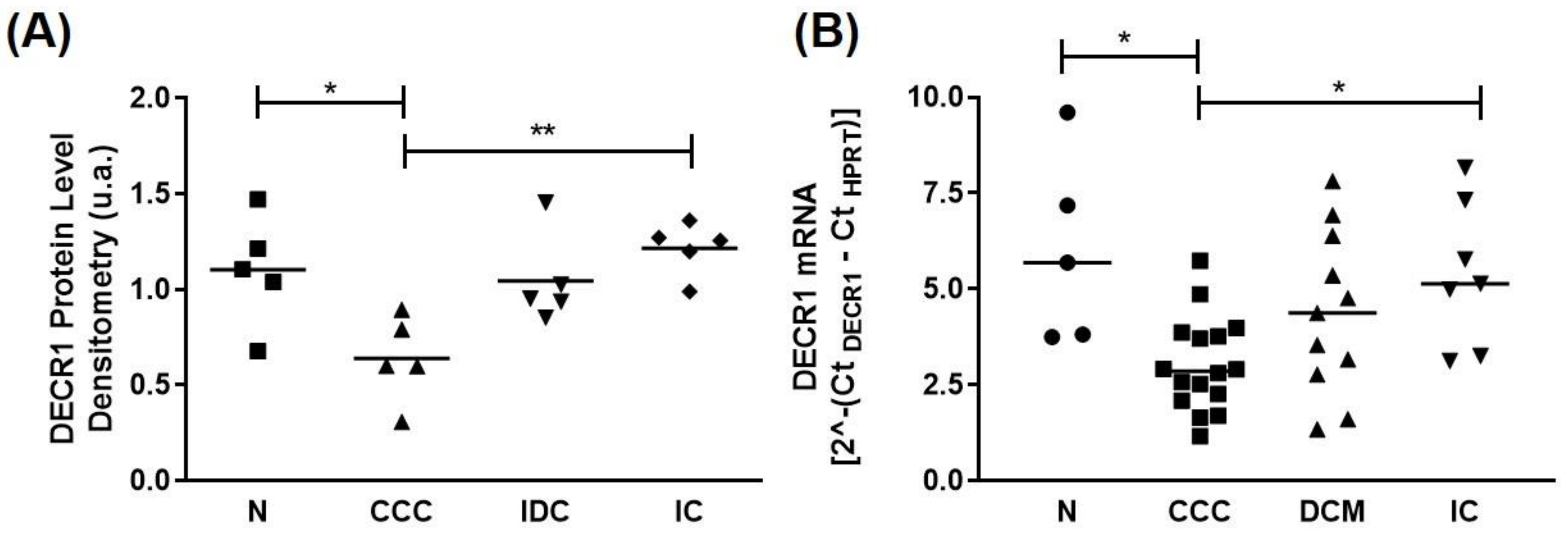

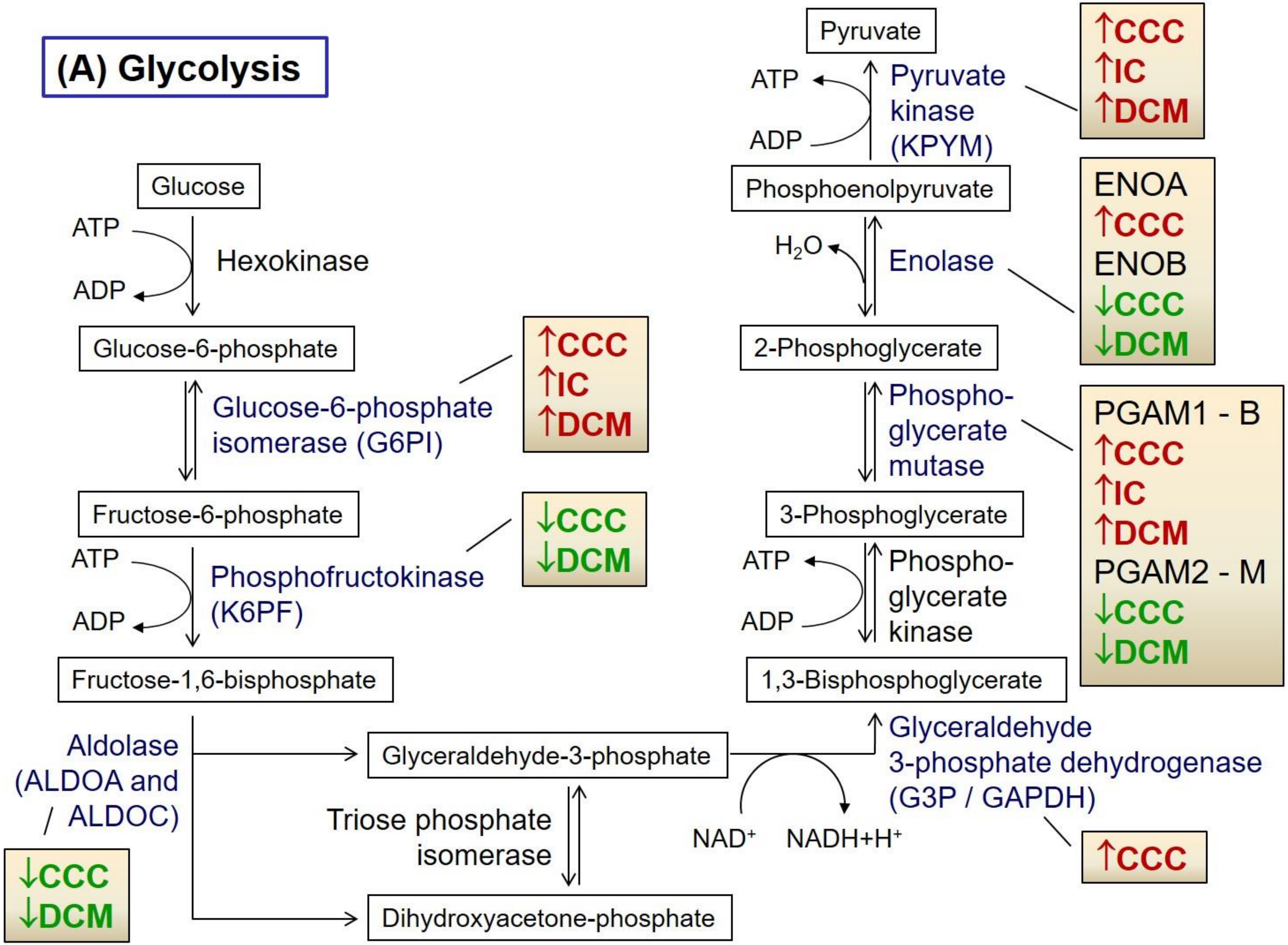

Supplemental Figure S7a

Supplemental Figure S7b

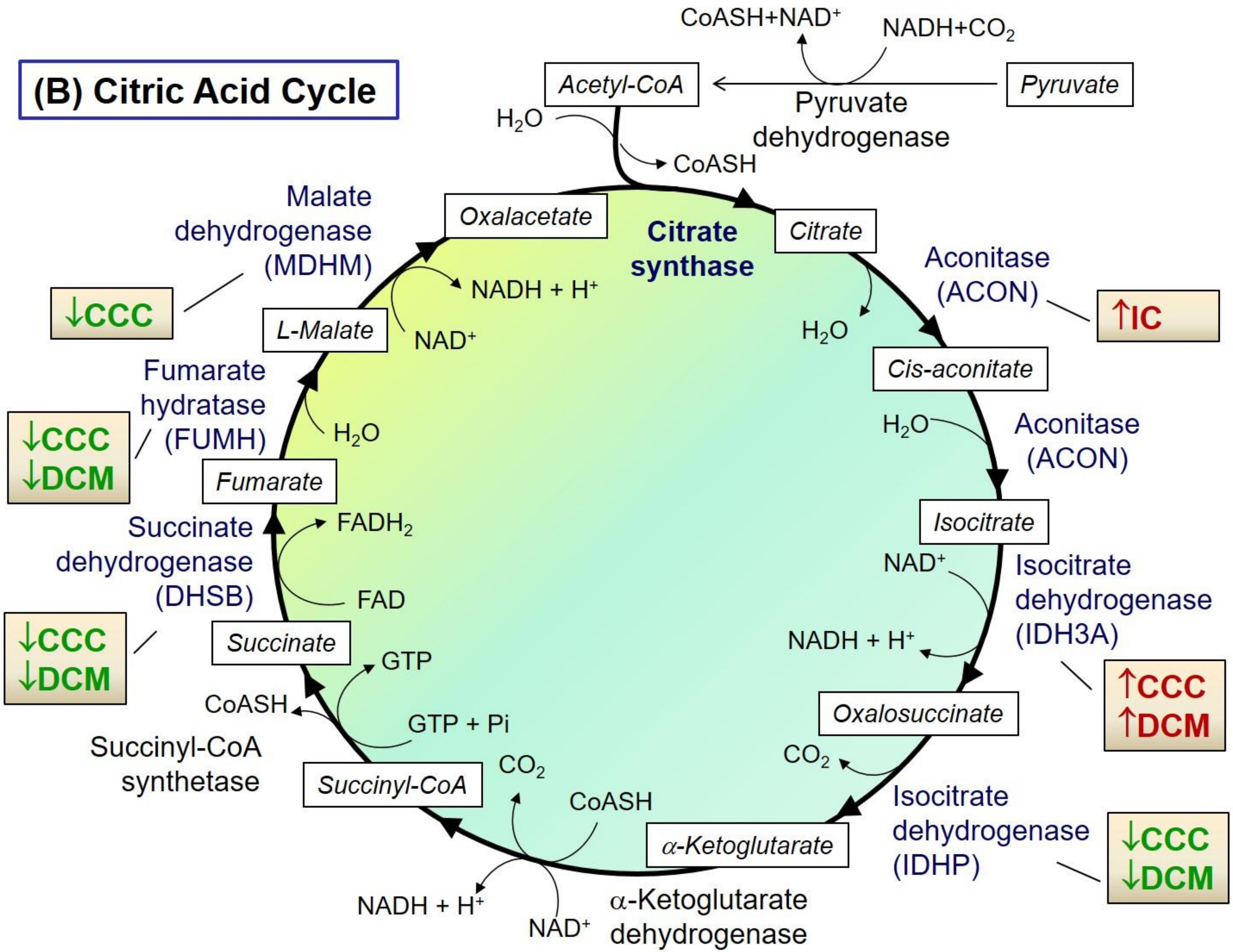

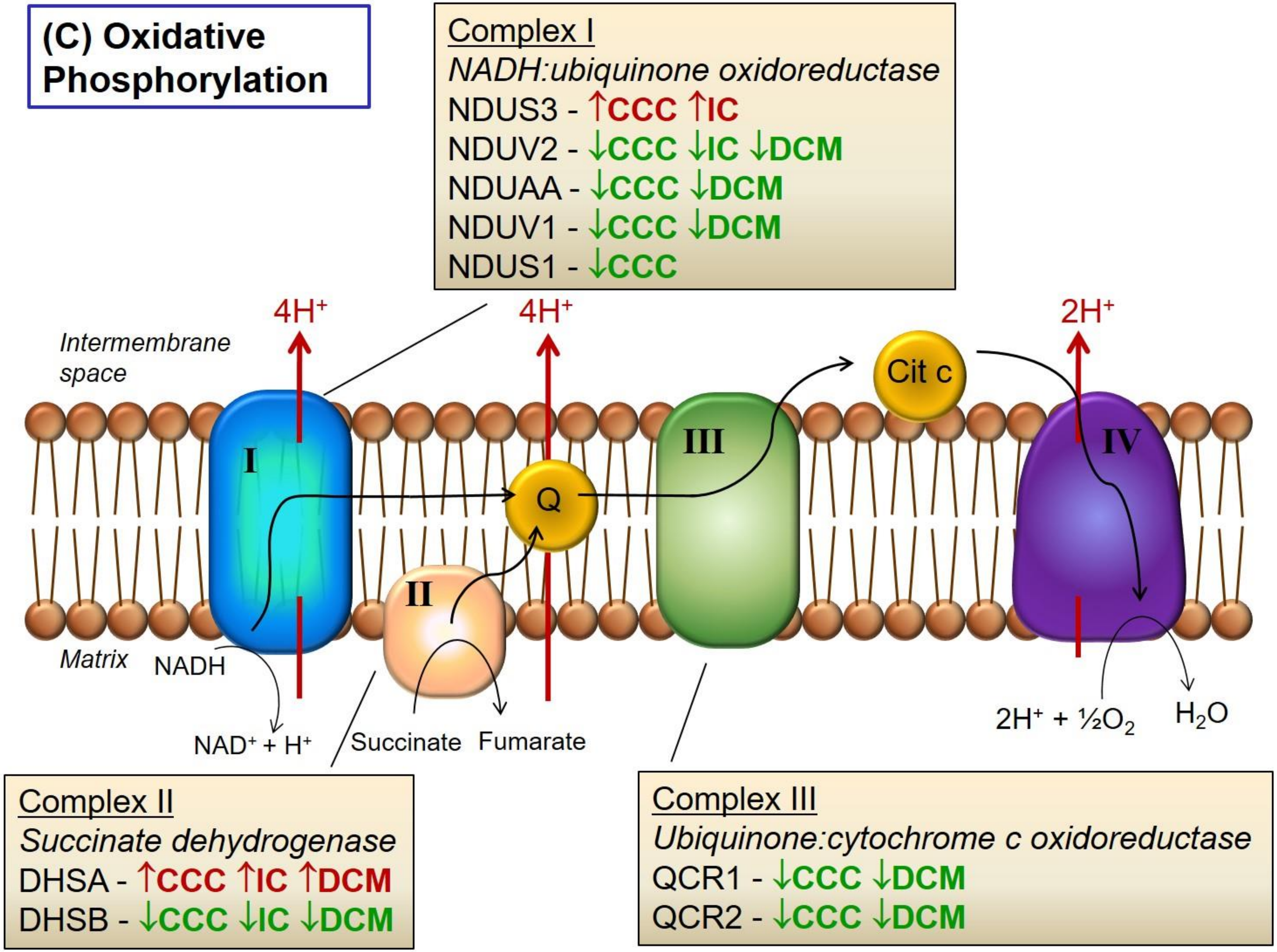

Supplemental Figure S7c

Supplemental
Figure S7d

(D) Creatine Kinase System

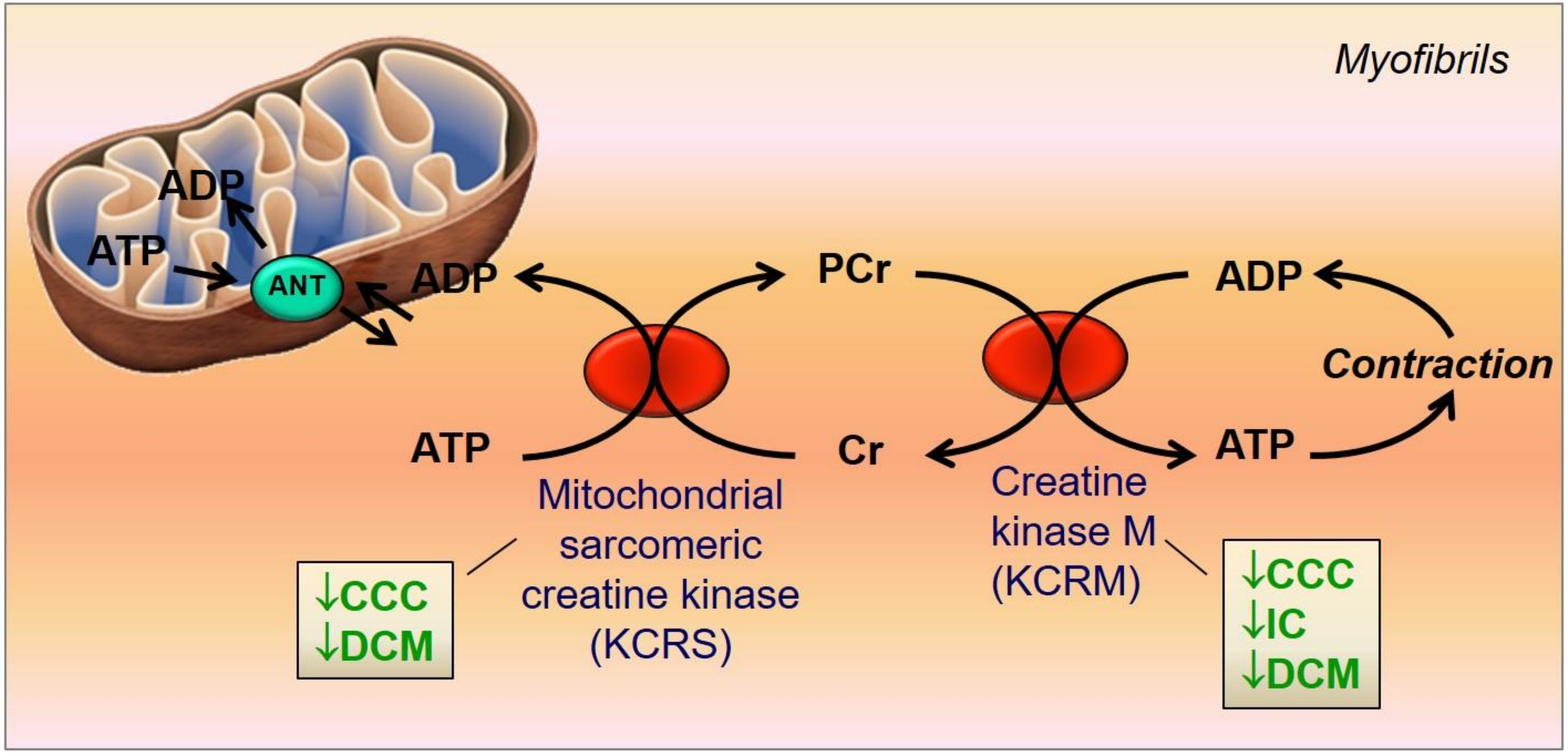

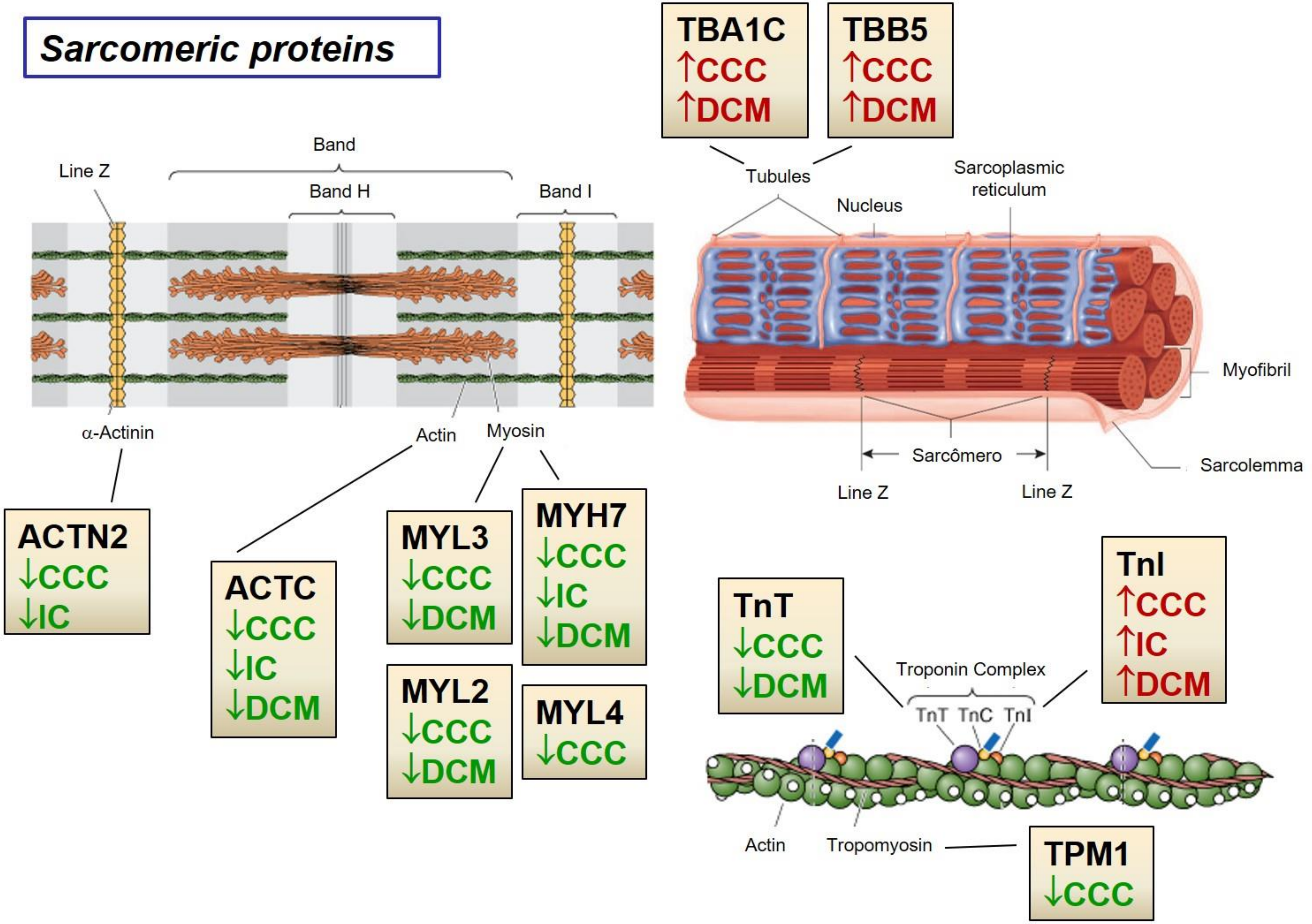

Supplemental Figure S8

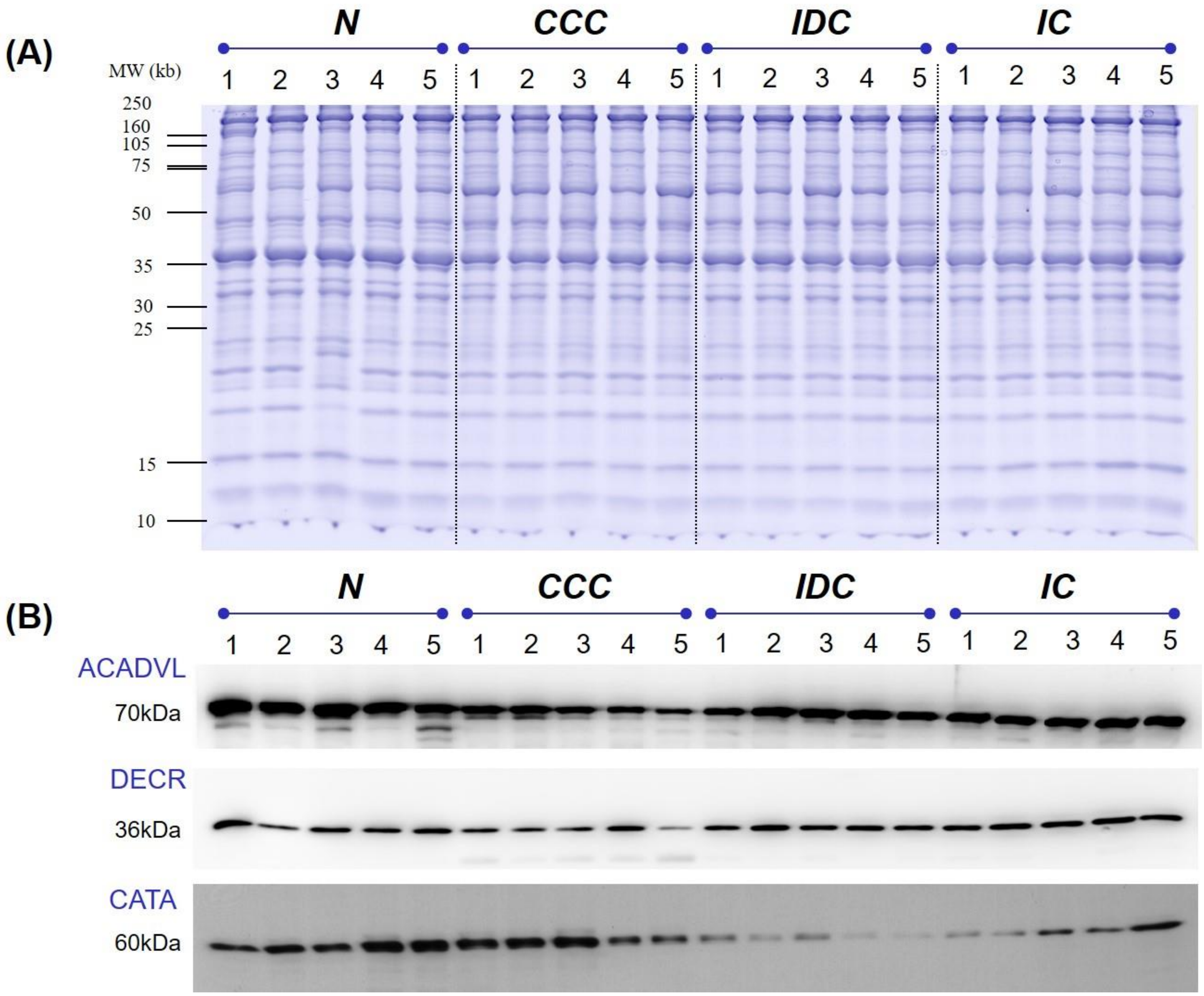

Supplemental Figure S9
