# Supplementary material for: Impairment of Multiple Mitochondrial Energy Metabolism Pathways in the Heart of Chagas Disease Cardiomyopathy Patients
Source: Front Immunol. 2021 Nov 12;12:755782. doi: 10.3389/fimmu.2021.755782 (PMC8633876; doi:10.3389/fimmu.2021.755782)
Supplement: Supplementary file 2 [file Table_1.docx]

**Table S1.** Baseline characteristics

| Etiol.-Patient ID* | Gender | Age | EF (%)^†^ | RVDD (cm)^‡^ | BMI (kg/m^2^) | Fibrosis^§^ | Myocarditis^\|\|^ |
| --- | --- | --- | --- | --- | --- | --- | --- |
| CCC #1 | M | 50 | 11% | 82 | 1.453 | 2+ | 2/3+ |
| CCC #2 | M | 57 | 29% | 71 | 1.642 | 1+ | 2/3+ |
| CCC #3 | M | 58 | 29% | 64 | 1.872 | 2+ | 2+ |
| CCC #4 | M | 59 | 17% | 64 | 1.375 | 2+ | 3+ |
| CCC #5 | M | 36 | 25% | 68 | 1.67 | 1+ | 2+ |
| CCC #6 | F | 49 | 37% | 77 | 1.435 | 3+ | 3+ |
| CCC #7 | F | 23 | 39% | 78 | 1.658 | 2+ | 2/3+ |
| CCC #8 | F | 61 | 15% | 76 | 1.747 | 1+ | 1+ |
| CCC #9 | F | 44 | 21% | 70 | 1.475 | 1+ | 0/1+ |
| CCC #10 | M | 15 | 17% | 62 | 1.613 | 1+ | 1+ |
| CCC #11 | F | 49 | 15% | 83 | 1.425 | 2+ | 3+ |
| CCC #12 | M | 28 | 21% | 68 | 1.848 | 2+ | 2/3+ |
| CCC #13 | F | 54 | 36% | 62 | 1.71 | 2+ | 2+ |
| CCC #14 | F | 60 | 20% | 72 | 1.614 | 2+ | 3+ |
| CCC #15 | F | 61 | 27% | 77 | 1.738 | 1/2+ | 0 |
| CCC #16 | F | 50 | 23% | 61 | 1.665 | 2/3+ | 3+ |
| CCC #17 | M | 32 | 12% | 75 | 1.844 | 2/3+ | 3+ |
| DCM #1 | M | 53 | 19% | 77 | 1.566 | 1/2+ | 0 |
| DCM #2 | M | 55 | 25% | 51 | 1.866 | 3+ | 0 |
| DCM #3 | M | 56 | 16% | 99 | 1.848 | 2+ | 0 |
| DCM #4 | M | 61 | 27% | 76 | 1.913 | na | na |
| DCM #5 | M | 36 | 14% | 62 | 1.844 | 0/1+ | 0 |
| DCM #6 | M | 38 | 16% | 88 | 1.784 | 0/1+ | 0 |
| DCM #7 | M | 15 | na | na | 1.772 | na | na |
| DCM #8 | F | 12 | 22% | 75 | 1.334 | 0/1+ | 1+ |
| DCM #9 | F | 58 | 17% | 78 | 1.555 | na | na |
| DCM #10 | F | 53 | 27% | 74 | 1.435 | na | na |
| DCM #11 | M | 15 | 29% | 94 | 1.918 | na | na |
| IC #1 | M | 49 | 25% | 76 | 1.79 | 1+ | 0 |
| IC #2 | M | 61 | 33% | 79 | 1.892 | 3+ | 0 |
| IC #3 | M | 52 | 20% | 62 | 1.859 | 3+ | 0 |
| IC #4 | M | 55 | 16% | 83 | 1.776 | na | na |
| IC #5 | M | 63 | 25% | 74 | 1.847 | 2+ | 0 |
| IC #6 | M | 62 | 37% | 75 | 1.491 | 2/3+ | 0 |
| N #1 | M | 17 | na | na | na | na | na |
| N #2 | M | 22 | na | na | na | na | na |
| N #3 | M | 28 | na | na | na | na | na |
| N #4 | M | 40 | na | na | na | na | na |
| N #5 | M | 46 | na | na | na | na | na |

* Patient ID: Patient Identification (#1 to #4 – included in the Proteomic Analysis and Malondialdehyde Quantification / #1 to #5 - included in the Immunoblotting analysis / All patients included in mRNA quantification). * Etiol.: Etiology, † EF: Ejection Fraction (reference value: ≥55%), ‡ LVDD: Left Ventricular Diastolic Diameter (reference value: 39-53mm), § and || as rated by histopatology (0: absent, 1 +: mild, 2 +: moderate, 3 +: intense), BMI: Body Mass Index, N: individuals without cardiomyopathies, CCC: chronic Chagas disease cardiomyopathy, DCM: idiopathic dilated cardiomyopathy, IC: ischemic cardiomyopathy, M: Male, na: not applicable or not available.
