# Supplementary material for: Impairment of Multiple Mitochondrial Energy Metabolism Pathways in the Heart of Chagas Disease Cardiomyopathy Patients
Source: Front Immunol. 2021 Nov 12;12:755782. doi: 10.3389/fimmu.2021.755782 (PMC8633876; doi:10.3389/fimmu.2021.755782)
Supplement: Supplementary file 3 [file Table_2.pdf]

Table S2. List of analysed spots by 2D-DIGE and protein identification by mass spectrometry in the myocardium from patients with CCC, IC, DCM and individuals without cardiomyopathies

Index	*UniProt*	*Symbol*	*Protein description*	*Cellular Component*	*Function*	*Theoretical Values*		*MS Identification*		*CCC / N*		*IC / N*		*DCM / N*		*1-ANOVA*
						pI	*MW (kDa)*	*Score*	*Peptides*	*Av. Ratio*	*T-test*	*Av. Ratio*	*T-test*	*Av. Ratio*	*T-test*	
1	Q96BH1	RNF25	E3 ubiquitin-protein ligase	Cytoplasm	8. Proteasome-Ubiquitin Process	6.10	51.22	72*	7	1.6	4.50E-03	1.02	0.821	1.53	3.79E-05	8.86E-06
2	P18206	VINC	Vinculin (Metavinculin)	Cytoskeleton	1. Structural and Contractile Proteins	5.51	123.67	65*	9	1.42	0.0104	1.02	0.975	1.48	1.84E-04	1.10E-05
3	P18206	VINC	Vinculin (Metavinculin)	Cytoskeleton	1. Structural and Contractile Proteins	5.51	123.67	6.33	5	1.24	0.0546	-1.05	0.541	1.3	6.98E-03	3.40E-04
4	NI	NI	NI - Spot not identified by MS	NI	10. NI - Spot not identified by MS	NI	NI	NI	NI	1.04	0.591	-1.18	0.0243	1.03	0.864	0.0574
5	NP	NP	NP - Spot not present in the preparative gel	NP	11. NP - Spot not present in the preparative gel	NP	NP	NP	NP	1.5	1.54E-03	1.2	0.122	1.46	2.59E-03	1.52E-03
6	NP	NP	NP - Spot not present in the preparative gel	NP	11. NP - Spot not present in the preparative gel	NP	NP	NP	NP	1.3	0.0691	1.23	0.165	1.32	0.197	0.0557
7	P14625	ENPL	Endoplasmin precursor (Heat shock protein 90 kDa beta member 1)	Endoplasmic reticulum	3. Stress Response and Apoptosis	4.73	90.18	25.86	5	2.74	5.10E-05	1.48	0.0332	2.21	1.07E-03	3.39E-07
8	NI	NI	NI - Spot not identified by MS	NI	10. NI - Spot not identified by MS	NI	NI	NI	NI	1.06	0.235	1.08	0.0242	1.22	1.66E-03	2.48E-03
9	NI	NI	NI - Spot not identified by MS	NI	10. NI - Spot not identified by MS	NI	NI	NI	NI	1.02	0.666	1.05	0.314	1.17	5.02E-03	0.0134
10	NI	NI	NI - Spot not identified by MS	NI	10. NI - Spot not identified by MS	NI	NI	NI	NI	-1.04	0.454	-1.09	0.0411	1.06	0.155	0.198
11	Q02218	ODO1	2-oxoglutarate dehydrogenase E1 component, mitochondrial	Mitochondria	2.1. Energy Metabolism - Glycolysis	6.07	111.34	10.44	10	-1.04	0.47	-1.07	0.199	1.06	0.295	0.531
12	Q6WKZ4	RFIP1	Rab11 family-interacting protein 1	Cytoplasm	9. Other Functions	5.32	137.20	54*	6	1.07	0.552	-1	0.979	1.06	0.649	0.942
13	O75112	LDB3	LIM domain-binding protein 3	Cytoskeleton	1. Structural and Contractile Proteins	8.47	77.13	59*	5	-1.72	1.88E-04	-1.44	8.52E-04	-1.46	1.29E-03	1.25E-05
14	O75112	LDB3	LIM domain-binding protein 3	Cytoskeleton	1. Structural and Contractile Proteins	8.47	77.13	63*	7	-1.96	6.89E-05	-1.53	1.22E-03	-1.6	6.34E-04	1.76E-06
15	O75112	LDB3	LIM domain-binding protein 3	Cytoskeleton	1. Structural and Contractile Proteins	8.47	77.13	70*	8	-2.18	2.55E-05	-1.6	3.34E-03	-1.77	3.28E-04	1.45E-06
16	O75112	LDB3	LIM domain-binding protein 3	Cytoskeleton	1. Structural and Contractile Proteins	8.47	77.13	5.43	7	-2.31	8.93E-05	-1.66	6.00E-03	-1.86	7.19E-04	3.45E-06
17	O75112	LDB3	LIM domain-binding protein 3	Cytoskeleton	1. Structural and Contractile Proteins	8.47	77.13	56*	5	-1.96	3.83E-04	-1.54	7.11E-03	-1.6	1.71E-03	5.62E-05
18	NP	NP	NP - Spot not present in the preparative gel	NP	11. NP - Spot not present in the preparative gel	NP	NP	NP	NP	1.07	0.796	-1.2	0.127	1.03	0.939	0.0216
19	P49588	SYAC	Alanyl-tRNA synthetase, cytoplasmic	Cytoplasm	6. Transcription and Translation Processes	5.34	106.81	53*	4	1.15	0.0678	1.15	0.172	1.02	0.847	0.114
20	NP	NP	NP - Spot not present in the preparative gel	NP	11. NP - Spot not present in the preparative gel	NP	NP	NP	NP	1.65	0.0711	1.11	0.453	1.51	0.036	0.0269
21	P14625	ENPL	Endoplasmin precursor (Heat shock protein 90 kDa beta member 1)	Endoplasmic reticulum	3. Stress Response and Apoptosis	4.73	90.18	7.06	6	1.13	0.596	-1.19	0.353	1.1	0.866	0.0241
22	P29323	EPHB2	Ephrin type-B receptor 2	Membrane	9. Other Functions	6.00	115.63	59*	7	1.51	0.126	-1.04	0.927	1.43	0.0446	0.043
23	NI	NI	NI - Spot not identified by MS	NI	10. NI - Spot not identified by MS	NI	NI	NI	NI	1.19	0.125	1.14	0.197	1.14	0.0895	0.323
24	NP	NP	NP - Spot not present in the preparative gel	NP	11. NP - Spot not present in the preparative gel	NP	NP	NP	NP	1.46	0.372	-1.13	0.581	1.28	0.284	0.0449
25	NP	NP	NP - Spot not present in the preparative gel	NP	11. NP - Spot not present in the preparative gel	NP	NP	NP	NP	1.7	0.0466	1.09	0.717	1.45	7.78E-03	9.75E-04
26	Q9UJD0	RIMS3	Regulating synaptic Membrane exocytosis protein 3	Membrane	9. Other Functions	9.38	32.80	53*	5	1.44	0.0117	1.21	0.166	1.15	0.165	0.0147
27	NI	NI	NI - Spot not identified by MS	NI	10. NI - Spot not identified by MS	NI	NI	NI	NI	1.31	0.0882	1.06	0.661	1	0.877	0.261
28	Q96GE5	ZN799	Zinc finger protein 799	Nucleus	6. Transcription and Translation Processes	9.29	74.29	53*	6	1.03	0.879	1.13	0.259	1.79	1.21E-03	0.0225
29	NP	NP	NP - Spot not present in the preparative gel	NP	11. NP - Spot not present in the preparative gel	NP	NP	NP	NP	-1.03	0.947	-1.05	0.787	1.33	0.38	0.684
30	P35609	ACTN2	Alpha-actinin-2 (Alpha actinin skeletal muscle isoform 2)	Cytoskeleton	1. Structural and Contractile Proteins	5.31	103.85	62*	7	-1.15	0.207	1.07	0.623	1.08	0.797	0.528
31	P06396	GELS	Gelsolin precursor (Actin-depolymerizing factor)	Cytoskeleton	1. Structural and Contractile Proteins	5.72	82.96	40*	5	-1.02	0.826	1.02	0.748	1.09	0.459	0.864
32	NI	NI	NI - Spot not identified by MS	NI	10. NI - Spot not identified by MS	NI	NI	NI	NI	1.36	0.0264	1.15	0.127	1.29	9.71E-03	7.53E-04
33	P06396	GELS	Gelsolin precursor (Actin-depolymerizing factor)	Cytoskeleton	1. Structural and Contractile Proteins	5.72	82.96	7.74	4	2.16	2.31E-04	1.28	0.0114	1.76	5.19E-05	1.76E-04
34	P06396	GELS	Gelsolin precursor (Actin-depolymerizing factor)	Cytoskeleton	1. Structural and Contractile Proteins	5.72	82.96	62*	9	2.35	3.10E-04	1.42	6.37E-03	2.01	2.46E-05	6.76E-05
35	Q96N16	JKIP1	Janus kinase and microtubule-interacting protein 1	Membrane	9. Other Functions	5.84	73.21	60*	8	-1.9	2.22E-03	-1.65	0.0181	-1.95	2.61E-03	4.62E-03

36	NI	NI	NI - Spot not identified by MS	NI	10. NI - Spot not identified by MS	NI	NI	NI	NI	1.55	3.30E-03	1.16	0.0595	1.4	6.43E-05	6.35E-05
37	P06396	GELS	Gelsolin precursor (Actin-depolymerizing factor)	Cytoskeleton	1. Structural and Contractile Proteins	5.72	82.96	10.29	8	1.63	2.62E-03	1.13	0.08	1.48	3.04E-07	5.39E-06
38	P06396	GELS	Gelsolin precursor (Actin-depolymerizing factor)	Cytoskeleton	1. Structural and Contractile Proteins	5.72	82.96	26.39	7	1.85	5.99E-04	1.21	0.0698	1.62	1.50E-06	4.86E-06
39	P06396	GELS	Gelsolin precursor (Actin-depolymerizing factor)	Cytoskeleton	1. Structural and Contractile Proteins	5.72	82.96	26.39	7	1.74	1.04E-03	1.1	0.335	1.54	4.16E-05	3.45E-05
40	P21980	TGM2	Protein-glutamine gamma-glutamyltransferase 2	Cytoplasm	2.6. Other Metabolic Processes	5.11	77.20	9.19	8	2.2	1.36E-03	1.27	0.0176	2.27	8.87E-08	4.55E-09
41	NI	NI	NI - Spot not identified by MS	NI	10. NI - Spot not identified by MS	NI	NI	NI	NI	-1.12	0.294	-1.31	0.0936	1.37	0.775	0.274
42	NP	NP	NP - Spot not present in the preparative gel	NP	11. NP - Spot not present in the preparative gel	NP	NP	NP	NP	-1.33	0.0195	-1.05	0.798	-1.23	0.0723	0.0126
43	NI	NI	NI - Spot not identified by MS	NI	10. NI - Spot not identified by MS	NI	NI	NI	NI	-1.03	0.548	-1.04	0.472	1.01	0.944	0.998
44	P21980	TGM2	Protein-glutamine gamma-glutamyltransferase 2	Cytoplasm	2.6. Other Metabolic Processes	5.11	77.20	11.07	6	2.41	2.58E-03	1.29	2.93E-03	2.3	4.18E-09	3.58E-09
45	NP	NP	NP - Spot not present in the preparative gel	NP	11. NP - Spot not present in the preparative gel	NP	NP	NP	NP	1.15	0.0387	1.22	5.46E-03	1.17	0.0218	0.0963
46	Q99798	ACON	Aconitate hydratase, mitochondrial (Aconitase)	Mitochondria	2.3. Energy Metabolism - Tricarboxylic Acid Cycle	6.85	82.43	8.74	5	-1.07	0.393	1.05	0.335	-1.01	0.915	0.723
47	Q99798	ACON	Aconitate hydratase, mitochondrial (Aconitase)	Mitochondria	2.3. Energy Metabolism - Tricarboxylic Acid Cycle	6.85	82.43	34.41	5	1.01	0.975	1.15	0.025	1.04	0.417	0.0526
48	Q99798	ACON	Aconitate hydratase, mitochondrial (Aconitase)	Mitochondria	2.3. Energy Metabolism - Tricarboxylic Acid Cycle	6.85	82.43	7.51	4	-1.19	0.0482	1.1	0.177	-1.08	0.308	0.122
49	NP	NP	NP - Spot not present in the preparative gel	NP	11. NP - Spot not present in the preparative gel	NP	NP	NP	NP	-1.61	7.47E-04	-1.25	0.0351	-1.48	2.52E-03	1.79E-03
50	P21980	TGM2	Protein-glutamine gamma-glutamyltransferase 2	Cytoplasm	2.6. Other Metabolic Processes	5.11	77.20	11.86	12	2.39	3.04E-03	1.24	0.0141	2.26	2.25E-08	2.35E-09
51	NP	NP	NP - Spot not present in the preparative gel	NP	11. NP - Spot not present in the preparative gel	NP	NP	NP	NP	-1.21	0.0819	-1.34	0.0417	1.16	0.976	0.209
52	NP	NP	NP - Spot not present in the preparative gel	NP	11. NP - Spot not present in the preparative gel	NP	NP	NP	NP	1.48	1.42E-05	1.32	4.57E-04	1.45	5.71E-06	8.59E-07
53	NP	NP	NP - Spot not present in the preparative gel	NP	11. NP - Spot not present in the preparative gel	NP	NP	NP	NP	1.35	1.45E-03	1.29	1.20E-03	1.37	5.13E-05	6.25E-05
54	NP	NP	NP - Spot not present in the preparative gel	NP	11. NP - Spot not present in the preparative gel	NP	NP	NP	NP	1.44	1.47E-04	1.4	8.26E-05	1.45	5.91E-06	3.20E-06
55	NP	NP	NP - Spot not present in the preparative gel	NP	11. NP - Spot not present in the preparative gel	NP	NP	NP	NP	1.25	0.0179	1.22	0.0281	1.25	4.90E-03	8.80E-03
56	Q99798	ACON	Aconitate hydratase, mitochondrial (Aconitase)	Mitochondria	2.3. Energy Metabolism - Tricarboxylic Acid Cycle	6.85	82.43	8.08	7	-1.04	0.535	1.09	0.159	-1.03	0.53	0.0904
57	Q99798	ACON	Aconitate hydratase, mitochondrial (Aconitase)	Mitochondria	2.3. Energy Metabolism - Tricarboxylic Acid Cycle	6.85	82.43	11.26	14	-1.01	0.747	1.17	3.91E-03	1.02	0.506	0.0181
58	P35609	ACTN2	Alpha-actinin-2 (Alpha actinin skeletal muscle isoform 2)	Cytoskeleton	1. Structural and Contractile Proteins	5.31	103.85	76	8	-1.74	4.51E-03	-1.76	5.82E-03	-1.35	0.0927	0.0134
59	Q99798	ACON	Aconitate hydratase, mitochondrial (Aconitase)	Mitochondria	2.3. Energy Metabolism - Tricarboxylic Acid Cycle	6.85	82.43	8.84	5	-1.04	0.38	1.16	1.24E-03	1.02	0.434	0.0213
60	P49674	KC1E	Casein kinase I isoform epsilon	Cytoplasm	5. Cell Signaling	9.68	47.32	56*	5	-1.56	4.53E-04	-1.23	0.0448	-1.43	5.37E-04	6.74E-03
61	NP	NP	NP - Spot not present in the preparative gel	NP	11. NP - Spot not present in the preparative gel	NP	NP	NP	NP	-1.4	2.86E-03	-1.13	0.227	-1.41	5.19E-03	4.90E-03
62	NP	NP	NP - Spot not present in the preparative gel	NP	11. NP - Spot not present in the preparative gel	NP	NP	NP	NP	1.34	7.77E-06	1.2	2.67E-05	1.34	7.26E-07	2.01E-06
63	Q99798	ACON	Aconitate hydratase, mitochondrial (Aconitase)	Mitochondria	2.3. Energy Metabolism - Tricarboxylic Acid Cycle	6.85	82.43	13.06	23	-1.05	0.337	1.17	4.15E-03	1.01	0.723	0.053
64	P29400	CO4A5	Collagen alpha-5(IV) chain	Cytoskeleton	1. Structural and Contractile Proteins	7.53	158.35	51*	6	1.03	0.608	-1.04	0.75	-1.02	0.934	0.955
65	Q8WWW8	GAB3	GRB2-associated-binding protein 3	Cytoplasm	9. Other Functions	6.80	65.59	58*	4	-1.33	6.97E-03	-1.26	0.0167	-1.44	4.13E-03	0.0437
66	NP	NP	NP - Spot not present in the preparative gel	NP	11. NP - Spot not present in the preparative gel	NP	NP	NP	NP	2.27	4.54E-03	1.45	0.0947	1.64	0.0196	2.76E-03
67	Q99798	ACON	Aconitate hydratase, mitochondrial (Aconitase)	Mitochondria	2.3. Energy Metabolism - Tricarboxylic Acid Cycle	6.85	82.43	14.69	21	-1.08	0.194	1.14	0.0361	-1	0.905	0.151
68	Q16891	IMMT	Mitochondrial inner Membrane protein (Mitofilin)	Mitochondria	9. Other Functions	6.08	83.68	16.4	5	1.29	1.61E-05	1.16	5.52E-04	1.28	4.87E-06	6.90E-06
69	P13010	KU86	ATP-dependent DNA helicase 2 subunit 2	Nucleus	2.6. Other Metabolic Processes	5.55	82.57	57*	4	-1.53	2.21E-04	-1.18	0.0591	-1.52	2.83E-05	8.16E-07
70	P08237	K6PF	6-phosphofructokinase, muscle type	Cytoplasm	2.1. Energy Metabolism - Glycolysis	8.23	85.05	72*	6	-1.27	3.02E-03	-1.09	0.101	-1.25	6.87E-04	4.85E-04
71	Q9H223	EHD4	EH domain-containing protein 4	Membrane	9. Other Functions	6.33	61.18	53*	5	-1.44	1.16E-05	-1.14	0.0982	-1.45	6.04E-07	2.98E-08
72	P08237	K6PF	6-phosphofructokinase, muscle type	Cytoplasm	2.1. Energy Metabolism - Glycolysis	8.23	85.05	60*	4	-1.09	0.268	-1.24	0.0506	-1.23	0.0255	0.253

73	Q16891	IMMT	Mitochondrial inner Membrane protein (Mitofilin)	Mitochondria	9. Other Functions	6.08	83.68	32.39	8	1.3	4.47E-05	1.18	8.89E-04	1.3	1.43E-05	8.89E-06
74	Q5TYW1	ZN658	Zinc finger protein 658	Nucleus	6. Transcription and Translation Processes	8.63	122.27	57*	11	1.59	6.39E-04	1.31	0.0166	1.53	1.54E-04	2.13E-04
75	Q6ZNA5	FRRS1	Ferric-chelate reductase 1	Membrane	7. Transport and Transmembrane Transport	7.16	63.81	52*	6	1.69	8.20E-06	1.59	3.19E-04	1.84	4.31E-08	3.05E-07
76	Q16891	IMMT	Mitochondrial inner Membrane protein (Mitofilin)	Mitochondria	9. Other Functions	6.08	83.68	6.49	7	1.3	3.59E-03	1.29	0.0144	1.41	5.32E-05	4.81E-04
77	P02787	TRFE	Serotransferrin precursor (Transferrin)	Secreted	9. Other Functions	6.70	75.20	6.52	8	2.84	1.01E-04	1.82	7.18E-03	2.6	1.74E-04	8.64E-06
78	P02787	TRFE	Serotransferrin precursor (Transferrin)	Secreted	9. Other Functions	6.70	75.20	9.35	9	2.69	5.59E-04	1.93	3.08E-03	2.23	2.40E-04	1.15E-03
79	Q16891	IMMT	Mitochondrial inner Membrane protein (Mitofilin)	Mitochondria	9. Other Functions	6.08	83.68	6.16	9	1.26	0.0466	1.02	0.767	1.23	0.131	0.151
80	Q9H115	SNAB	Beta-soluble NSF attachment protein	Membrane	7. Transport and Transmembrane Transport	5.32	33.56	56*	5	2.93	7.08E-05	1.84	8.29E-03	2.55	9.47E-05	7.67E-06
81	P02787	TRFE	Serotransferrin precursor (Transferrin)	Secreted	9. Other Functions	6.70	75.20	11.59	10	1.66	0.0174	1.2	0.335	1.53	0.0302	0.0933
82	P02787	TRFE	Serotransferrin precursor (Transferrin)	Secreted	9. Other Functions	6.70	75.20	13.68	14	3.46	1.79E-04	2.35	4.03E-03	3.17	1.83E-05	1.88E-05
83	NP	NP	NP - Spot not present in the preparative gel	NP	11. NP - Spot not present in the preparative gel	NP	NP	NP	NP	2.31	1.39E-03	1.16	0.553	1.68	0.0453	1.02E-03
84	P28331	NDUS1	NADH-ubiquinone oxidoreductase 75 kDa subunit, mitochondrial	Mitochondria	2.4. Energy Metabolism - Oxidative Phosphorylation and Electron Transport	5.42	76.98	26.47	7	-1.2	0.0925	-1.11	0.187	-1.21	0.0522	0.0836
85	P28331	NDUS1	NADH-ubiquinone oxidoreductase 75 kDa subunit, mitochondrial	Mitochondria	2.4. Energy Metabolism - Oxidative Phosphorylation and Electron Transport	5.42	76.98	13.93	7	-1.25	2.81E-03	-1.02	0.662	-1.15	0.0789	0.013
86	P28331	NDUS1	NADH-ubiquinone oxidoreductase 75 kDa subunit, mitochondrial	Mitochondria	2.4. Energy Metabolism - Oxidative Phosphorylation and Electron Transport	5.42	76.98	44.12	10	-1.25	1.81E-03	-1.01	0.94	-1.18	0.0183	0.021
87	P11021	GRP78	78 kDa glucose-regulated protein (Heat shock 70 kDa protein 5)	Endoplasmic reticulum	3. Stress Response and Apoptosis	5.01	70.48	14.69	16	1.64	1.62E-04	1.1	0.47	1.64	1.11E-03	4.95E-06
88	P29597	TYK2	Non-receptor tyrosine-protein kinase TYK2	Cytoplasm	5. Cell Signaling	6.71	133.65	56*	7	-1.15	0.226	-1.19	0.128	-1.07	0.573	0.595
89	P11021	GRP78	78 kDa glucose-regulated protein (Heat shock 70 kDa protein 5)	Endoplasmic reticulum	3. Stress Response and Apoptosis	5.01	70.48	43.5	14	2	1.62E-05	1.23	0.134	1.94	9.52E-05	2.32E-07
90	NI	NI	NI - Spot not identified by MS	NI	10. NI - Spot not identified by MS	NI	NI	NI	NI	1.28	0.171	-1.06	0.59	1.2	0.0371	3.92E-03
91	P12883	MYH7	Myosin-7 (Myosin heavy chain, cardiac muscle beta isoform)	Cytoskeleton	1. Structural and Contractile Proteins	5.63	223.10	6.64	8	-2.2	6.88E-03	-2.56	3.34E-03	-2.76	1.73E-03	6.56E-05
92	NP	NP	NP - Spot not present in the preparative gel	NP	11. NP - Spot not present in the preparative gel	NP	NP	NP	NP	-1.02	0.862	-1.03	0.735	1	0.991	6.33E-04
93	NP	NP	NP - Spot not present in the preparative gel	NP	11. NP - Spot not present in the preparative gel	NP	NP	NP	NP	-1.86	0.0116	-2.13	6.96E-03	-2.25	3.84E-03	5.78E-05
94	P40939	ECHA	Trifunctional enzyme subunit alpha, mitochondrial	Mitochondria	2.2. Energy Metabolism - Lipid Metabolism/ß-Oxidation	8.98	79.01	9.07	8	-1.11	0.319	1.17	0.245	1.05	0.693	0.208
95	Q96MP8	KCTD7	BTB/POZ domain-containing protein KCTD7	Cytoplasm	7. Transport and Transmembrane Transport	5.58	33.13	57*	4	-1.29	0.0223	-1.2	0.0922	-1.22	0.0917	1.24E-03
96	NP	NP	NP - Spot not present in the preparative gel	NP	11. NP - Spot not present in the preparative gel	NP	NP	NP	NP	-1.13	0.239	-1	0.917	-1.16	0.195	0.301
97	Q5T5P2	SKT	Sickle tail protein homolog	Cytoplasm	9. Other Functions	6.59	214.12	53*	11	-1.32	9.70E-03	-1.26	0.0317	-1.23	0.0286	0.0435
98	P38646	GRP75	Stress-70 protein, mitochondrial	Mitochondria	3. Stress Response and Apoptosis	5.44	68.76	12.3	14	1.65	4.56E-05	1.53	5.14E-06	1.68	2.63E-05	7.92E-06
99	NP	NP	NP - Spot not present in the preparative gel	NP	11. NP - Spot not present in the preparative gel	NP	NP	NP	NP	2.03	7.27E-03	1.36	0.253	2.14	9.76E-03	0.0253
100	NI	NI	NI - Spot not identified by MS	NI	10. NI - Spot not identified by MS	NI	NI	NI	NI	2.21	2.02E-04	1.54	9.00E-03	1.94	1.90E-04	4.30E-04
101	P51884	LUM	Lumican precursor	Cytoskeleton	1. Structural and Contractile Proteins	6.17	36.66	27	4	2.68	1.04E-04	1.92	1.97E-03	2.3	4.83E-04	1.54E-03
102	P17661	DESM	Desmin	Cytoplasm	1. Structural and Contractile Proteins	5.21	53.40	6.04	11	1.82	9.20E-04	1.25	0.177	1.8	2.28E-04	1.03E-03
103	P38646	GRP75	Stress-70 protein, mitochondrial	Mitochondria	3. Stress Response and Apoptosis	5.44	68.76	76*	12	1.68	4.52E-04	1.5	2.41E-04	1.62	2.48E-04	6.74E-05
104	NI	NI	NI - Spot not identified by MS	NI	10. NI - Spot not identified by MS	NI	NI	NI	NI	2.62	9.89E-05	1.57	0.016	2.13	1.00E-03	1.01E-03
105	P01024	CO3	Complement C3 precursor	Secreted	4. Immune Response	6.00	184.95	57*	6	2.44	6.58E-06	1.65	7.74E-03	2.25	5.37E-04	2.75E-04
106	P17661	DESM	Desmin	Cytoplasm	1. Structural and Contractile Proteins	5.21	53.40	9.69	8	1.31	0.0325	1.07	0.535	1.32	0.0221	0.109
107	P11142	HSP7C	Heat shock cognate 71 kDa protein (Heat shock 70 kDa protein 8)	Cytoplasm	3. Stress Response and Apoptosis	5.37	70.77	34.98	14	1.25	0.0153	1.16	0.0208	1.26	2.29E-03	3.50E-03
108	NP	NP	NP - Spot not present in the preparative gel	NP	11. NP - Spot not present in the preparative gel	NP	NP	NP	NP	1.24	0.0446	1.05	0.606	1.12	0.222	0.335
109	P11142	HSP7C	Heat shock cognate 71 kDa protein (Heat shock 70 kDa protein 8)	Cytoplasm	3. Stress Response and Apoptosis	5.37	70.77	10.6	10	1.2	0.0557	1.13	0.0928	1.21	8.89E-03	5.14E-03

110	P02768	ALBU	Serum albumin precursor	Secreted	9. Other Functions	5.67	66.47	7.09	8	2.46	1.34E-04	1.59	0.0124	1.96	1.03E-04	3.43E-04
111	NP	NP	NP - Spot not present in the preparative gel	NP	11. NP - Spot not present in the preparative gel	NP	NP	NP	NP	2.79	2.36E-04	2.04	3.33E-03	2.28	4.50E-04	2.64E-05
112	NP	NP	NP - Spot not present in the preparative gel	NP	11. NP - Spot not present in the preparative gel	NP	NP	NP	NP	2.22	5.92E-04	1.51	0.0375	1.81	5.91E-04	2.19E-03
113	NP	NP	NP - Spot not present in the preparative gel	NP	11. NP - Spot not present in the preparative gel	NP	NP	NP	NP	2.19	4.39E-04	1.48	0.0503	1.72	1.64E-03	2.88E-03
114	NP	NP	NP - Spot not present in the preparative gel	NP	11. NP - Spot not present in the preparative gel	NP	NP	NP	NP	2.29	3.59E-05	1.87	3.21E-03	1.88	1.83E-04	8.69E-05
115	P02768	ALBU	Serum albumin precursor	Secreted	9. Other Functions	5.67	66.47	12.96	12	2.45	8.20E-05	1.92	3.43E-03	2.01	2.64E-04	4.87E-05
116	NP	NP	NP - Spot not present in the preparative gel	NP	11. NP - Spot not present in the preparative gel	NP	NP	NP	NP	2.47	1.35E-04	1.8	6.03E-03	1.96	3.74E-04	1.94E-05
117	P02768	ALBU	Serum albumin precursor	Secreted	9. Other Functions	5.67	66.47	12.32	13	2.48	2.56E-04	2.29	2.27E-03	2.03	9.03E-04	3.63E-04
118	P31040	DHSA	Succinate dehydrogenase [ubiquinone] flavoprotein subunit, mitochondrial	Mitochondria	2.4. Energy Metabolism - Oxidative Phosphorylation and Electron Transport	6.25	68.01	8.47	10	2.3	1.63E-04	2.07	3.55E-03	1.96	4.69E-04	2.23E-04
119	P31040	DHSA	Succinate dehydrogenase [ubiquinone] flavoprotein subunit, mitochondrial	Mitochondria	2.4. Energy Metabolism - Oxidative Phosphorylation and Electron Transport	6.25	68.01	7.3	8	2.3	3.95E-04	1.94	5.51E-03	1.83	1.70E-03	5.79E-04
120	NI	NI	NI - Spot not identified by MS	NI	10. NI - Spot not identified by MS	NI	NI	NI	NI	-1.05	0.591	1.02	0.716	-1.01	0.966	0.672
121	O95831	AIFM1	Apoptosis-inducing factor 1, mitochondrial	Nucleus	3. Stress Response and Apoptosis	6.86	55.81	58*	7	1.03	0.512	1.09	0.148	1.06	0.318	0.499
122	NP	NP	NP - Spot not present in the preparative gel	NP	11. NP - Spot not present in the preparative gel	NP	NP	NP	NP	1.24	0.0322	1.1	0.235	1.25	0.0301	0.151
123	P08107	HSP71	Heat shock 70 kDa protein 1	Endoplasmic reticulum	3. Stress Response and Apoptosis	5.48	69.92	14.8	15	1.45	4.11E-03	1.31	0.0186	1.4	3.04E-03	3.72E-03
124	P00488	F13A	Coagulation factor XIII A chain	Cytoplasm	9. Other Functions	5.81	79.24	56*	8	1.41	9.68E-03	1.09	0.413	1.41	4.90E-03	6.12E-03
125	P08107	HSP71	Heat shock 70 kDa protein 1	Endoplasmic reticulum	3. Stress Response and Apoptosis	5.48	69.92	14.96	20	1.41	0.0119	1.28	0.0243	1.38	4.85E-03	2.00E-03
126	NI	NI	NI - Spot not identified by MS	NI	10. NI - Spot not identified by MS	NI	NI	NI	NI	1.05	0.641	-1.4	0.0851	1.58	0.0207	0.0706
127	NP	NP	NP - Spot not present in the preparative gel	NP	11. NP - Spot not present in the preparative gel	NP	NP	NP	NP	1.63	0.0256	1.11	0.538	1.34	0.242	0.0746
128	P31040	DHSA	Succinate dehydrogenase [ubiquinone] flavoprotein subunit, mitochondrial	Mitochondria	2.4. Energy Metabolism - Oxidative Phosphorylation and Electron Transport	6.25	68.01	15.94	14	1.02	1	1.29	0.0143	1.02	0.757	0.0638
129	P31040	DHSA	Succinate dehydrogenase [ubiquinone] flavoprotein subunit, mitochondrial	Mitochondria	2.4. Energy Metabolism - Oxidative Phosphorylation and Electron Transport	6.25	68.01	14.98	14	-1	0.874	1.22	0.0148	-1	0.961	0.0425
130	NP	NP	NP - Spot not present in the preparative gel	NP	11. NP - Spot not present in the preparative gel	NP	NP	NP	NP	1.7	0.0144	1.1	0.696	1.62	0.0417	0.0126
131	P12883	MYH7	Myosin-7 (Myosin heavy chain, cardiac muscle beta isoform)	Cytoskeleton	1. Structural and Contractile Proteins	5.63	223.10	61*	9	-1.71	0.0877	-1.8	0.063	-2.01	0.0204	1.22E-03
132	NP	NP	NP - Spot not present in the preparative gel	NP	11. NP - Spot not present in the preparative gel	NP	NP	NP	NP	1.15	0.298	1.03	0.762	1.05	0.441	0.489
133	NI	NI	NI - Spot not identified by MS	NI	10. NI - Spot not identified by MS	NI	NI	NI	NI	1.14	0.0122	1.05	0.248	1.07	0.247	0.0325
134	Q9H6S0	YTDC2	Probable ATP-dependent RNA helicase YTHDC2	Nucleus	2.6. Other Metabolic Processes	8.68	160.25	59*	6	-1.02	0.821	1.01	0.766	1.02	0.77	8.42E-04
135	NP	NP	NP - Spot not present in the preparative gel	NP	11. NP - Spot not present in the preparative gel	NP	NP	NP	NP	1.29	8.16E-03	1.05	0.757	1.21	3.01E-03	8.44E-03
136	P49748	ACADVL	Very long-chain specific acyl-CoA dehydrogenase, mitochondrial	Mitochondria	2.2. Energy Metabolism - Lipid Metabolism/ß-Oxidation	7.74	66.18	9.14	7	-1.52	3.16E-03	1.08	0.29	-1.15	0.198	2.33E-03
137	P49748	ACADVL	Very long-chain specific acyl-CoA dehydrogenase, mitochondrial	Mitochondria	2.2. Energy Metabolism - Lipid Metabolism/ß-Oxidation	7.74	66.18	14.69	13	-1.51	3.06E-03	-1.05	0.483	-1.22	0.0741	2.02E-03
138	NP	NP	NP - Spot not present in the preparative gel	NP	11. NP - Spot not present in the preparative gel	NP	NP	NP	NP	1.06	0.593	-1.09	0.2	1.13	0.0467	0.0198
139	NP	NP	NP - Spot not present in the preparative gel	NP	11. NP - Spot not present in the preparative gel	NP	NP	NP	NP	1	0.818	1.21	0.177	1.09	0.593	0.56
140	P42345	FRAP	Serine/threonine-protein kinase mTOR	Endoplasmic reticulum	5. Cell Signaling	6.73	288.89	60*	12	-1.39	0.0542	-1.38	0.0498	-1.1	0.506	0.0461
141	P49748	ACADVL	Very long-chain specific acyl-CoA dehydrogenase, mitochondrial	Mitochondria	2.2. Energy Metabolism - Lipid Metabolism/ß-Oxidation	7.74	66.18	26.47	12	-1.11	0.296	-1.05	0.657	1.02	0.988	2.81E-04
142	Q16555	DPYL2	Dihydropyrimidinase-related protein 2	Cytoplasm	2.6. Other Metabolic Processes	5.95	62.29	11.43	9	1.35	5.67E-03	1.12	0.0582	1.35	2.26E-05	5.06E-05
143	Q92736	RYR2	Ryanodine receptor 2	Membrane	7. Transport and Transmembrane Transport	5.73	564.57	65*	9	-1.07	0.484	-1.12	0.279	-1.06	0.573	0.272
144	NI	NI	NI - Spot not identified by MS	NI	10. NI - Spot not identified by MS	NI	NI	NI	NI	-1.1	0.297	1	0.946	1.03	0.96	4.89E-04
145	P49748	ACADVL	Very long-chain specific acyl-CoA dehydrogenase, mitochondrial	Mitochondria	2.2. Energy Metabolism - Lipid Metabolism/ß-Oxidation	7.74	66.18	13.18	10	-1.36	5.96E-03	1.04	0.56	-1.04	0.635	7.89E-04
146	P49748	ACADVL	Very long-chain specific acyl-CoA dehydrogenase, mitochondrial	Mitochondria	2.2. Energy Metabolism - Lipid Metabolism/ß-Oxidation	7.74	66.18	202*	14	-1.23	0.0436	-1.01	0.976	-1.06	0.521	6.57E-04

147	Q0VD86	INCA1	Protein INCA1	Nucleus	9. Other Functions	6.92	26.82	50*	8	-1.59	0.064	-1.76	0.0256	-1.54	0.071	0.0118
148	Q16134	ETFD	Electron transfer flavoprotein-ubiquinone oxidoreductase, mitochondrial	Mitochondria	2.4. Energy Metabolism - Oxidative Phosphorylation and Electron Transport	6.52	64.68	50*	4	1.02	0.669	1.1	0.144	1.04	0.545	0.0623
149	NP	NP	NP - Spot not present in the preparative gel	NP	11. NP - Spot not present in the preparative gel	NP	NP	NP	NP	-1.2	0.0159	-1.01	0.873	-1.08	0.301	0.0773
150	P17661	DESM	Desmin	Cytoplasm	1. Structural and Contractile Proteins	5.21	53.40	15.58	20	1.44	4.11E-03	1.44	3.58E-03	1.81	4.66E-05	1.86E-06
151	NP	NP	NP - Spot not present in the preparative gel	NP	11. NP - Spot not present in the preparative gel	NP	NP	NP	NP	-1.17	0.152	1.03	0.677	-1.04	0.702	3.96E-03
152	NP	NP	NP - Spot not present in the preparative gel	NP	11. NP - Spot not present in the preparative gel	NP	NP	NP	NP	-1.47	0.0454	-1.43	0.0722	-1.08	0.608	0.0104
153	A8MWA6	F90AM	Putative protein FAM90A22	Other	9. Other Functions	9.86	49.87	73*	5	1.26	9.96E-03	1.06	0.789	1.13	0.0543	0.164
154	NP	NP	NP - Spot not present in the preparative gel	NP	11. NP - Spot not present in the preparative gel	NP	NP	NP	NP	1.29	0.04	-1.04	0.573	-1.04	0.773	0.333
155	Q7Z3D6	CN159	UPF0317 protein C14orf159, mitochondrial	Mitochondria	9. Other Functions	5.87	63.15	60*	5	1.27	0.0538	-1.02	0.691	1.08	0.537	0.247
156	Q16134	ETFD	Electron transfer flavoprotein-ubiquinone oxidoreductase, mitochondrial	Mitochondria	2.4. Energy Metabolism - Oxidative Phosphorylation and Electron Transport	6.52	64.68	6.04	6	-1.02	0.894	1.32	0.0329	1.04	0.817	0.0761
157	Q16134	ETFD	Electron transfer flavoprotein-ubiquinone oxidoreductase, mitochondrial	Mitochondria	2.4. Energy Metabolism - Oxidative Phosphorylation and Electron Transport	6.52	64.68	7.4	9	-1.14	0.198	1.16	0.0831	-1.02	0.833	0.0229
158	Q16134	ETFD	Electron transfer flavoprotein-ubiquinone oxidoreductase, mitochondrial	Mitochondria	2.4. Energy Metabolism - Oxidative Phosphorylation and Electron Transport	6.52	64.68	6.67	5	-1.02	0.886	1.17	0.083	1.04	0.619	0.443
159	P17661	DESM	Desmin	Cytoplasm	1. Structural and Contractile Proteins	5.21	53.40	12.54	16	1.21	0.0849	1.25	0.0543	1.61	7.28E-05	1.81E-05
160	NP	NP	NP - Spot not present in the preparative gel	NP	11. NP - Spot not present in the preparative gel	NP	NP	NP	NP	-1.35	0.0604	-1.37	2.61E-03	-1.15	0.234	0.339
161	Q8TCG1	CIP2A	Protein CIP2A	Cytoplasm	9. Other Functions	5.85	102.19	53*	7	1.41	0.122	-1.11	0.8	1.61	0.0334	0.0702
162	P01009	A1AT	Alpha-1-antitrypsin precursor	Secreted	3. Stress Response and Apoptosis	5.37	44.32	11.13	7	1.32	0.167	-1.22	0.389	1.5	0.0514	0.0444
163	P01009	A1AT	Alpha-1-antitrypsin precursor	Secreted	3. Stress Response and Apoptosis	5.37	44.32	14.13	12	1.23	0.288	-1.29	0.189	1.4	0.111	0.0404
164	P27338	AOFB	Amine oxidase [flavin-containing] B	Mitochondria	2.6. Other Metabolic Processes	7.18	58.63	71*	6	1.31	0.0268	1.61	2.63E-03	1.56	1.46E-03	0.0267
165	Q13114	TRAF3	TNF receptor-associated factor 3	Cytoplasm	3. Stress Response and Apoptosis	8.23	64.49	52*	5	1.24	0.0279	1.03	0.639	1.24	3.45E-03	0.017
166	P36871	PGM1	Phosphoglucomutase-1	Cytoplasm	2.6. Other Metabolic Processes	6.32	61.32	6.53	5	1.09	0.429	1.24	0.0127	1.14	0.113	0.356
167	P01009	A1AT	Alpha-1-antitrypsin precursor	Secreted	3. Stress Response and Apoptosis	5.37	44.32	14.99	11	1.18	0.419	-1.24	0.164	1.31	0.205	0.0587
168	NI	NI	NI - Spot not identified by MS	NI	10. NI - Spot not identified by MS	NI	NI	NI	NI	1.4	1.55E-03	1.39	5.65E-03	1.57	8.64E-05	2.21E-05
169	NI	NI	NI - Spot not identified by MS	NI	10. NI - Spot not identified by MS	NI	NI	NI	NI	1.12	0.56	-1.07	0.475	1.15	0.132	0.269
170	Q13114	TRAF3	TNF receptor-associated factor 3	Cytoplasm	3. Stress Response and Apoptosis	8.23	64.49	50*	5	1.7	3.36E-06	1.35	4.42E-03	1.85	1.36E-08	2.30E-09
171	P30038	AL4A1	Delta-1-pyrroline-5-carboxylate dehydrogenase, mitochondrial	Mitochondria	2.4. Energy Metabolism - Oxidative Phosphorylation and Electron Transport	6.96	59.03	9.28	6	1.43	1.62E-04	1.32	3.73E-04	1.52	2.90E-05	7.64E-07
172	NP	NP	NP - Spot not present in the preparative gel	NP	11. NP - Spot not present in the preparative gel	NP	NP	NP	NP	1.96	4.58E-04	1.13	0.211	1.47	2.07E-03	7.71E-05
173	P10809	CH60	60 kDa heat shock protein, mitochondrial	Mitochondria	3. Stress Response and Apoptosis	5.24	57.96	18.32	12	1.09	0.275	1.25	0.0144	1.19	0.0429	1.98E-03
174	P07237	PDIA1	Protein disulfide-isomerase precursor	Endoplasmic reticulum	3. Stress Response and Apoptosis	4.69	55.29	16.16	4	2.01	2.41E-04	1.26	0.0638	1.78	1.77E-05	2.15E-07
175	Q13325	IFIT5	Interferon-induced protein with tetratricopeptide repeats 5	Cytoplasm	9. Other Functions	7.00	55.85	43*	4	1.41	0.0132	1	0.946	1.55	6.27E-04	2.75E-04
176	P01009	A1AT	Alpha-1-antitrypsin precursor	Secreted	3. Stress Response and Apoptosis	5.37	44.32	11.33	9	1.15	0.546	-1.13	0.349	1.31	0.103	0.0852
177	NP	NP	NP - Spot not present in the preparative gel	NP	11. NP - Spot not present in the preparative gel	NP	NP	NP	NP	-1.17	0.205	-1.38	0.021	-1.13	0.306	0.19
178	P04040	CATA	Catalase	Cytoplasm	3. Stress Response and Apoptosis	6.95	59.62	6.44	5	1.48	6.48E-04	1.04	0.69	1.21	0.0166	0.0212
179	NP	NP	NP - Spot not present in the preparative gel	NP	11. NP - Spot not present in the preparative gel	NP	NP	NP	NP	1.03	1	-1	0.945	-1.03	0.74	0.639
180	NP	NP	NP - Spot not present in the preparative gel	NP	11. NP - Spot not present in the preparative gel	NP	NP	NP	NP	1.28	0.0144	-1.03	0.724	1.28	4.05E-04	2.85E-05
181	P04040	CATA	Catalase	Cytoplasm	3. Stress Response and Apoptosis	6.95	59.62	45*	4	1.22	7.22E-03	1.13	0.0924	1.17	0.0338	0.029
182	P14618	KPYM	Pyruvate kinase isozymes M1/M2	Cytoplasm	2.1. Energy Metabolism - Glycolysis	7.95	57.81	72*	5	1.18	0.0421	1.13	0.0158	1.14	6.04E-03	0.0418
183	P14618	KPYM	Pyruvate kinase isozymes M1/M2	Cytoplasm	2.1. Energy Metabolism - Glycolysis	7.95	57.81	8.6	7	1.16	0.0307	1.15	7.02E-03	1.16	5.64E-03	5.19E-03

184	NI	NI	NI - Spot not identified by MS	NI	10. NI - Spot not identified by MS	NI	NI	NI	NI	1.63	3.78E-04	1.25	0.0193	1.34	2.65E-03	3.78E-04
185	P14618	KPYM	Pyruvate kinase isozymes M1/M2	Cytoplasm	2.1. Energy Metabolism - Glycolysis	7.95	57.81	59*	4	1.02	0.748	1.13	0.0573	1.05	0.409	8.39E-03
186	NP	NP	NP - Spot not present in the preparative gel	NP	11. NP - Spot not present in the preparative gel	NP	NP	NP	NP	-1.46	0.012	-1.49	0.0138	-1.37	0.0461	0.147
187	Q9BQE3	TBA1C	Tubulin alpha-1C chain	Cytoskeleton	1. Structural and Contractile Proteins	4.96	49.90	12.22	8	1.81	1.51E-03	1.32	0.0255	1.92	2.79E-06	2.97E-04
188	NI	NI	NI - Spot not identified by MS	NI	10. NI - Spot not identified by MS	NI	NI	NI	NI	1.62	7.70E-04	1.03	0.613	1.45	3.00E-04	2.21E-07
189	Q76G19	PDZD4	PDZ domain-containing protein 4	Cytoplasm	9. Other Functions	5.80	86.17	58*	8	1.63	1.28E-04	1.15	0.0862	1.37	1.30E-03	3.74E-05
190	P30101	PDIA3	Protein disulfide-isomerase A3 precursor	Endoplasmic reticulum	3. Stress Response and Apoptosis	5.61	54.27	8.67	9	1.67	1.02E-03	1.13	0.119	1.51	4.99E-05	1.12E-07
191	P30101	PDIA3	Protein disulfide-isomerase A3 precursor	Endoplasmic reticulum	3. Stress Response and Apoptosis	5.61	54.27	11.54	11	1.64	8.83E-05	1.06	0.387	1.48	7.49E-06	9.70E-09
192	NP	NP	NP - Spot not present in the preparative gel	NP	11. NP - Spot not present in the preparative gel	NP	NP	NP	NP	1.5	0.0214	-1.06	0.616	1.47	0.0106	4.39E-03
193	Q9HCC0	MCCB	Methylcrotonoyl-CoA carboxylase beta chain, mitochondrial	Mitochondria	2.6. Other Metabolic Processes	6.47	58.99	9.78	6	1.22	1.31E-04	1	0.949	1.14	0.243	0.0864
194	Q96AX9	MIB2	E3 ubiquitin-protein ligase MIB2	Cytoplasm	8. Proteasome-Ubiquitin Process	8.81	109.94	64*	9	1.32	2.31E-03	1.02	0.787	1.29	6.79E-03	9.46E-03
195	NI	NI	NI - Spot not identified by MS	NI	10. NI - Spot not identified by MS	NI	NI	NI	NI	1.64	1.39E-03	1.35	0.0133	1.59	6.54E-04	7.01E-06
196	P09622	DLDH	Dihydrolipoyl dehydrogenase, mitochondrial precursor	Mitochondria	2.4. Energy Metabolism - Oxidative Phosphorylation and Electron Transport	6.50	50.17	8.12	4	1.41	2.78E-05	1.14	0.0649	1.34	1.47E-03	1.01E-05
197	P09622	DLDH	Dihydrolipoyl dehydrogenase, mitochondrial precursor	Mitochondria	2.4. Energy Metabolism - Oxidative Phosphorylation and Electron Transport	6.50	50.17	10.9	7	1.23	7.52E-05	1.14	7.12E-03	1.21	2.94E-03	3.85E-04
198	P09622	DLDH	Dihydrolipoyl dehydrogenase, mitochondrial precursor	Mitochondria	2.4. Energy Metabolism - Oxidative Phosphorylation and Electron Transport	6.50	50.17	8.19	4	1.21	1.72E-03	1.2	0.0105	1.22	5.98E-03	6.65E-03
199	P42345	FRAP	Serine/threonine-protein kinase mTOR	Endoplasmic reticulum	5. Cell Signaling	6.73	288.89	51*	10	-1.54	0.0501	-1.7	0.0276	-1.26	0.252	3.91E-03
200	P07437	TBB5	Tubulin beta-5 chain	Cytoskeleton	1. Structural and Contractile Proteins	4.78	49.67	6.28	6	1.76	2.13E-04	1.21	0.111	1.76	3.74E-08	1.63E-05
201	P07437	TBB5	Tubulin beta-5 chain	Cytoskeleton	1. Structural and Contractile Proteins	4.78	49.67	8.66	9	1.44	1.25E-03	1.1	0.507	1.42	2.15E-05	2.59E-04
202	P07437	TBB5	Tubulin beta-5 chain	Cytoskeleton	1. Structural and Contractile Proteins	4.78	49.67	9.48	10	1.16	0.142	-1.07	0.483	1.1	0.343	0.116
203	NP	NP	NP - Spot not present in the preparative gel	NP	11. NP - Spot not present in the preparative gel	NP	NP	NP	NP	-1.42	0.0509	-1.36	0.0967	-1.01	0.879	0.0307
204	NP	NP	NP - Spot not present in the preparative gel	NP	11. NP - Spot not present in the preparative gel	NP	NP	NP	NP	1.19	0.044	-1	0.733	1.09	0.0717	0.0142
205	NI	NI	NI - Spot not identified by MS	NI	10. NI - Spot not identified by MS	NI	NI	NI	NI	1.6	4.27E-05	1.26	5.20E-03	1.23	0.0605	3.11E-05
206	P06744	G6PI	Glucose-6-phosphate isomerase	Cytoplasm	2.1. Energy Metabolism - Glycolysis	8.44	63.02	10.26	6	2.39	1.32E-04	1.49	4.47E-03	1.53	1.67E-03	4.41E-06
207	Q9BSD4	YP016	Uncharacterized protein MGC16385	Other	9. Other Functions	undefined	undefined	66*	4	2.44	1.18E-06	1.33	5.01E-03	1.66	4.55E-05	1.43E-08
208	P55809	SCOT1	Succinyl-CoA:3-ketoacid-coenzyme A transferase 1, mitochondrial	Mitochondria	2.6. Other Metabolic Processes	6.00	52.09	69*	6	1.38	7.56E-03	1.02	0.997	1.21	3.86E-03	1.97E-04
209	NI	NI	NI - Spot not identified by MS	NI	10. NI - Spot not identified by MS	NI	NI	NI	NI	1.44	0.0273	1.14	0.284	1.38	2.33E-04	1.62E-03
210	NP	NP	NP - Spot not present in the preparative gel	NP	11. NP - Spot not present in the preparative gel	NP	NP	NP	NP	-1.24	0.417	-1.43	0.135	-1.21	0.433	0.0227
211	NP	NP	NP - Spot not present in the preparative gel	NP	11. NP - Spot not present in the preparative gel	NP	NP	NP	NP	3.35	5.74E-08	1.71	4.27E-03	1.92	1.94E-06	2.50E-06
212	P01857	IGHG1	Ig gamma-1 chain C region.	Secreted	4. Immune Response	undefined	undefined	9	5	4.19	8.66E-08	2	2.80E-03	2.43	9.91E-07	4.82E-07
213	P17661	DESM	Desmin	Cytoplasm	1. Structural and Contractile Proteins	5.21	53.40	65*	9	1.95	1.02E-03	1.77	8.61E-03	2.92	5.77E-06	9.02E-09
214	P01857	IGHG1	Ig gamma-1 chain C region.	Secreted	4. Immune Response	undefined	undefined	9.97	5	5.09	3.10E-06	2.07	2.33E-03	2.7	1.66E-06	8.71E-07
215	NI	NI	NI - Spot not identified by MS	NI	10. NI - Spot not identified by MS	NI	NI	NI	NI	1.43	9.00E-04	-1.03	0.868	1.06	0.38	2.07E-04
216	P00367	DHE3	Glutamate dehydrogenase 1, mitochondrial	Mitochondria	2.6. Other Metabolic Processes	6.71	56.01	7.84	5	1.5	2.65E-05	1.16	9.86E-03	1.29	2.44E-04	1.16E-06
217	NP	NP	NP - Spot not present in the preparative gel	NP	11. NP - Spot not present in the preparative gel	NP	NP	NP	NP	1.85	6.94E-05	1.31	6.09E-03	1.51	9.06E-04	4.25E-05
218	Q02252	MMSA	Methylmalonate-semialdehyde dehydrogenase [acylating], mitochondrial	Mitochondria	2.6. Other Metabolic Processes	8.01	54.39	5.99	6	1.86	4.60E-05	1.3	9.31E-04	1.43	2.82E-05	9.27E-07
219	NI	NI	NI - Spot not identified by MS	NI	10. NI - Spot not identified by MS	NI	NI	NI	NI	1.58	1.72E-05	1.14	0.0477	1.34	6.75E-04	1.52E-08
220	P01857	IGHG1	Ig gamma-1 chain C region.	Secreted	4. Immune Response	undefined	undefined	7.65	5	4.32	7.87E-06	1.86	1.27E-03	2.43	1.42E-06	1.71E-06

221	NP	NP	NP - Spot not present in the preparative gel	NP	11. NP - Spot not present in the preparative gel	NP	NP	NP	NP	1.36	3.84E-05	1.05	0.19	1.18	4.40E-03	6.35E-07
222	Q8NEB9	PK3C3	Phosphatidylinositol 3-kinase catalytic subunit type 3	Cytoplasm	5. Cell Signaling	6.37	101.55	67*	10	-1.08	0.459	-1.09	0.451	1.21	0.0431	0.0485
223	P17661	DESM	Desmin	Cytoplasm	1. Structural and Contractile Proteins	5.21	53.40	11.01	7	1.29	0.0446	1.15	0.254	1.87	3.21E-05	5.17E-06
224	NP	NP	NP - Spot not present in the preparative gel	NP	11. NP - Spot not present in the preparative gel	NP	NP	NP	NP	1.61	5.36E-04	1.17	0.0766	1.34	0.0132	4.28E-04
225	P25705	ATPA	ATP synthase subunit alpha, mitochondrial	Mitochondria	2.4. Energy Metabolism - Oxidative Phosphorylation and Electron Transport	8.28	55.21	8.74	6	1.86	6.97E-05	1.29	4.08E-03	1.37	1.31E-04	9.37E-05
226	P17661	DESM	Desmin	Cytoplasm	1. Structural and Contractile Proteins	5.21	53.40	6.4	5	-1.01	0.924	-1.04	0.637	1.35	2.11E-03	1.56E-03
227	P25705	ATPA	ATP synthase subunit alpha, mitochondrial	Mitochondria	2.4. Energy Metabolism - Oxidative Phosphorylation and Electron Transport	8.28	55.21	111*	12	1.45	1.30E-05	1.17	5.14E-03	1.18	9.37E-03	1.88E-03
228	P05091	ALDH2	Aldehyde dehydrogenase, mitochondrial	Mitochondria	2.6. Other Metabolic Processes	5.69	54.44	6.95	6	-1.1	0.257	-1.1	0.224	1.05	0.437	0.168
229	P25705	ATPA	ATP synthase subunit alpha, mitochondrial	Mitochondria	2.4. Energy Metabolism - Oxidative Phosphorylation and Electron Transport	8.28	55.21	68*	7	1.17	0.0187	1.15	0.0674	1.03	0.714	0.0274
230	P05091	ALDH2	Aldehyde dehydrogenase, mitochondrial	Mitochondria	2.6. Other Metabolic Processes	5.69	54.44	10.81	10	-1.09	0.217	-1.1	0.155	-1.02	0.8	0.338
231	P05091	ALDH2	Aldehyde dehydrogenase, mitochondrial	Mitochondria	2.6. Other Metabolic Processes	5.69	54.44	13.65	6	-1.13	0.081	-1.07	0.245	-1.07	0.3	0.115
232	Q6P2E9	EDC4	Enhancer of mRNA-decapping protein 4	Cytoplasm	6. Transcription and Translation Processes	5.55	151.53	7.48	5	-1.13	0.12	-1.13	0.0697	-1.24	2.28E-03	2.64E-03
233	Q86TD4	SRCA	Sarcalumenin	Endoplasmic reticulum	6. Transcription and Translation Processes	4.33	98.73	92*	13	-1.01	0.866	-1.05	0.382	-1.07	0.14	2.36E-03
234	NI	NI	NI - Spot not identified by MS	NI	10. NI - Spot not identified by MS	NI	NI	NI	NI	1.62	1.10E-05	1.23	9.88E-03	1.39	2.72E-05	1.58E-06
235	NP	NP	NP - Spot not present in the preparative gel	NP	11. NP - Spot not present in the preparative gel	NP	NP	NP	NP	1.41	2.75E-03	1.08	0.143	1.21	7.42E-03	2.64E-03
236	P36957	ODO2	2-oxoglutarate dehydrogenase E2 component, mitochondrial	Mitochondria	2.3. Energy Metabolism - Tricarboxylic Acid Cycle	5.90	41.39	10.62	4	1.03	0.687	1.08	0.174	1.15	6.71E-03	0.0916
237	NI	NI	NI - Spot not identified by MS	NI	10. NI - Spot not identified by MS	NI	NI	NI	NI	1.01	0.846	-1.02	0.758	1.05	0.353	0.105
238	NP	NP	NP - Spot not present in the preparative gel	NP	11. NP - Spot not present in the preparative gel	NP	NP	NP	NP	1.35	4.46E-04	1.07	0.33	1.17	0.0208	2.17E-03
239	P28838	AMPL	Cytosol aminopeptidase	Cytoplasm	9. Other Functions	8.03	56.17	59*	6	1.49	9.24E-05	1.14	0.0252	1.23	5.25E-03	3.65E-04
240	P28838	AMPL	Cytosol aminopeptidase	Cytoplasm	9. Other Functions	8.03	56.17	60*	7	1.27	1.16E-04	1.02	0.816	1.04	0.392	2.34E-03
241	P25705	ATPA	ATP synthase subunit alpha, mitochondrial	Mitochondria	2.4. Energy Metabolism - Oxidative Phosphorylation and Electron Transport	8.28	55.21	89*	14	1.1	0.0122	1.03	0.593	1.02	0.579	1.26E-03
242	Q8IWZ8	SF04	Splicing factor 4	Nucleus	6. Transcription and Translation Processes	7.20	72.47	57*	7	1.12	0.301	1.03	0.742	1.07	0.489	0.795
243	P06576	ATPB	ATP synthase subunit beta, mitochondrial	Mitochondria	2.4. Energy Metabolism - Oxidative Phosphorylation and Electron Transport	5.00	51.77	11.71	6	-1.16	0.0635	-1.07	0.346	-1.21	0.0142	5.93E-03
244	P06576	ATPB	ATP synthase subunit beta, mitochondrial	Mitochondria	2.4. Energy Metabolism - Oxidative Phosphorylation and Electron Transport	5.00	51.77	12.43	6	-1.01	0.931	-1.11	0.366	-1.13	0.245	0.289
245	NI	NI	NI - Spot not identified by MS	NI	10. NI - Spot not identified by MS	NI	NI	NI	NI	1.64	1.20E-04	1.4	6.86E-03	1.38	0.0147	1.42E-03
246	P46734	MP2K3	Dual specificity mitogen-activated protein kinase kinase	Cytoplasm	6. Transcription and Translation Processes	7.05	39.32	63*	7	1.14	0.154	1.09	0.194	1.21	0.0293	0.0745
247	Q8NFQ5	BPIL3	Bactericidal/permeability-increasing protein-like 3	Secreted	9. Other Functions	8.85	47.79	55*	6	1.15	0.0641	1.17	4.45E-03	1.19	5.40E-04	1.63E-03
248	P56703	WNT3	Proto-oncogene protein Wnt-3	Cytoskeleton	5. Cell Signaling	7.53	37.48	58*	6	1.14	0.0831	1.11	0.29	1.21	0.0787	0.16
249	NP	NP	NP - Spot not present in the preparative gel	NP	11. NP - Spot not present in the preparative gel	NP	NP	NP	NP	-1.8	0.0281	-1.33	0.287	-1.26	0.293	0.0864
250	P11182	ODB2	Lipoamide acyltransferase, mitochondrial	Mitochondria	2.6. Other Metabolic Processes	6.17	46.37	57*	6	1.03	0.561	1.07	0.502	1.07	0.517	0.598
251	NI	NI	NI - Spot not identified by MS	NI	10. NI - Spot not identified by MS	NI	NI	NI	NI	-1.25	4.81E-03	-1.07	0.346	-1.24	5.94E-03	3.65E-04
252	P68104	EF1A1	Elongation factor 1-alpha 1	Cytoplasm	6. Transcription and Translation Processes	9.10	50.14	6.93	6	1	0.88	-1.04	0.578	1.05	0.403	0.79
253	NI	NI	NI - Spot not identified by MS	NI	10. NI - Spot not identified by MS	NI	NI	NI	NI	1.25	0.126	1.14	0.391	1.27	0.0905	0.398
254	P61158	ARP3	Actin-like protein 3	Cytoskeleton	1. Structural and Contractile Proteins	5.61	47.24	7.86	5	1.45	3.00E-03	-1.09	0.335	1.22	0.0167	1.85E-05
255	P42568	AF9	Protein AF-9	Nucleus	5. Cell Signaling	8.77	63.35	56*	6	1.12	0.258	-1.29	0.041	1.11	0.28	7.19E-03
256	P49821	NDUV1	NADH dehydrogenase [ubiquinone] flavoprotein 1, mitochondrial	Mitochondria	2.4. Energy Metabolism - Oxidative Phosphorylation and Electron Transport	7.53	48.51	14.33	10	-1.21	0.0295	1.07	0.292	-1.11	0.129	5.63E-04
257	NP	NP	NP - Spot not present in the preparative gel	NP	11. NP - Spot not present in the preparative gel	NP	NP	NP	NP	-1.46	0.073	-1.19	0.422	-1.33	0.175	0.227

258	P49821	NDUV1	NADH dehydrogenase [ubiquinone] flavoprotein 1, mitochondrial	Mitochondria	2.4. Energy Metabolism - Oxidative Phosphorylation and Electron Transport	7.53	48.51	11.29	10	-1.42	3.78E-03	-1.11	0.348	-1.25	0.0395	0.0872
259	NP	NP	NP - Spot not present in the preparative gel	NP	11. NP - Spot not present in the preparative gel	NP	NP	NP	NP	-1.33	0.0359	-1.11	0.368	-1.2	0.0676	0.248
260	Q75T13	PGAP1	GPI inositol-deacylase	Endoplasmic reticulum	9. Other Functions	9.14	105.38	56*	5	1.02	0.73	-1.07	0.704	1.03	0.736	0.936
261	P49821	NDUV1	NADH dehydrogenase [ubiquinone] flavoprotein 1, mitochondrial	Mitochondria	2.4. Energy Metabolism - Oxidative Phosphorylation and Electron Transport	7.53	48.51	10.53	7	-1.34	0.123	-1.15	0.453	-1.23	0.278	0.512
262	P49821	NDUV1	NADH dehydrogenase [ubiquinone] flavoprotein 1, mitochondrial	Mitochondria	2.4. Energy Metabolism - Oxidative Phosphorylation and Electron Transport	7.53	48.51	12.68	10	-1.4	1.40E-03	-1.12	0.211	-1.3	8.42E-03	3.55E-03
263	NI	NI	NI - Spot not identified by MS	NI	10. NI - Spot not identified by MS	NI	NI	NI	NI	1.43	5.36E-05	1.06	0.235	1.29	9.15E-05	3.67E-08
264	NI	NI	NI - Spot not identified by MS	NI	10. NI - Spot not identified by MS	NI	NI	NI	NI	1.34	5.42E-03	-1.03	0.465	1.09	0.121	6.31E-05
265	P14316	IRF2	Interferon regulatory factor 2	Nucleus	6. Transcription and Translation Processes	7.07	39.35	79*	4	1.31	0.0112	1.03	0.687	1.14	0.0642	4.03E-05
266	A8MQB9	YR007	Putative ankyrin repeat domain-containing protein	Other	9. Other Functions	5.74	27.02	63*	5	-1.42	0.0374	-1.22	0.279	-1.23	0.199	0.171
267	P35609	ACTN2	Alpha-actinin-2 (Alpha actinin skeletal muscle isoform 2)	Cytoskeleton	1. Structural and Contractile Proteins	5.31	103.85	7.32	4	-1.72	0.0264	-1.55	0.0716	-1.58	0.0504	0.0163
268	P06733	ENOA	Alpha-enolase (Non- neural enolase)	Cytoplasm	2.1. Energy Metabolism - Glycolysis	6.99	47.04	72*	5	-1.04	0.5	1.01	0.758	-1.05	0.399	0.188
269	P06733	ENOA	Alpha-enolase (Non- neural enolase)	Cytoplasm	2.1. Energy Metabolism - Glycolysis	6.99	47.04	7.28	5	-1	0.924	1.02	0.488	-1.02	0.596	0.565
270	P06733	ENOA	Alpha-enolase (Non- neural enolase)	Cytoplasm	2.1. Energy Metabolism - Glycolysis	6.99	47.04	61*	4	1.12	0.0766	-1.03	0.681	1.01	0.755	0.414
271	P06733	ENOA	Alpha-enolase (Non- neural enolase)	Cytoplasm	2.1. Energy Metabolism - Glycolysis	6.99	47.04	9.25	9	-1.06	0.404	1.05	0.26	-1.03	0.606	0.386
272	P06733	ENOA	Alpha-enolase (Non- neural enolase)	Cytoplasm	2.1. Energy Metabolism - Glycolysis	6.99	47.04	10.44	8	1.36	1.58E-03	1.01	0.945	1.08	0.182	8.55E-03
273	P48735	IDHP	Isocitrate dehydrogenase [NADP], mitochondrial	Mitochondria	2.3. Energy Metabolism - Tricarboxylic Acid Cycle	8.32	46.61	6.84	4	-1.38	2.58E-03	-1.27	0.0183	-1.4	2.35E-04	1.18E-05
274	P06733	ENOA	Alpha-enolase (Non- neural enolase)	Cytoplasm	2.1. Energy Metabolism - Glycolysis	6.99	47.04	11.29	9	1.15	0.128	-1.09	0.205	-1	0.987	0.0752
275	NI	NI	NI - Spot not identified by MS	NI	10. NI - Spot not identified by MS	NI	NI	NI	NI	-1.04	0.319	1.03	0.63	-1.05	0.14	0.0184
276	O75822	EIF3J	Eukaryotic translation initiation factor 3 subunit	Cytoplasm	6. Transcription and Translation Processes	4.72	28.93	55*	4	-1.27	1.55E-04	-1.12	0.0786	-1.22	5.29E-04	1.63E-04
277	P13929	ENOB	Beta-enolase (Skeletal muscle enolase)	Cytoplasm	2.1. Energy Metabolism - Glycolysis	7.73	46.80	6.55	6	-1.66	3.34E-04	-1.28	0.0124	-1.59	2.97E-04	8.21E-04
278	NP	NP	NP - Spot not present in the preparative gel	NP	11. NP - Spot not present in the preparative gel	NP	NP	NP	NP	-1.64	4.80E-05	-1.2	0.0839	-1.31	0.0223	4.88E-03
279	P31930	QCR1	Cytochrome b-c1 complex subunit 1, mitochondrial	Mitochondria	2.4. Energy Metabolism - Oxidative Phosphorylation and Electron Transport	5.43	49.13	26.47	6	-1.08	0.136	1.01	0.793	-1.1	0.0711	0.065
280	NI	NI	NI - Spot not identified by MS	NI	10. NI - Spot not identified by MS	NI	NI	NI	NI	-1.26	4.29E-03	-1.05	0.361	-1.24	3.23E-03	1.02E-04
281	P31930	QCR1	Cytochrome b-c1 complex subunit 1, mitochondrial	Mitochondria	2.4. Energy Metabolism - Oxidative Phosphorylation and Electron Transport	5.43	49.13	10.75	7	-1.18	6.06E-03	-1	0.982	-1.12	0.0452	1.90E-03
282	P07954	FUMH	Fumarate hydratase, mitochondrial	Mitochondria	2.3. Energy Metabolism - Tricarboxylic Acid Cycle	6.99	50.08	13.19	8	-1.09	0.229	1.1	0.194	-1.01	0.967	0.0226
283	NI	NI	NI - Spot not identified by MS	NI	10. NI - Spot not identified by MS	NI	NI	NI	NI	-1.41	1.78E-03	-1.19	0.141	-1.25	0.0185	5.03E-03
284	NI	NI	NI - Spot not identified by MS	NI	10. NI - Spot not identified by MS	NI	NI	NI	NI	-1.5	0.187	1.18	0.645	-1.29	0.404	0.309
285	NI	NI	NI - Spot not identified by MS	NI	10. NI - Spot not identified by MS	NI	NI	NI	NI	-1.07	0.374	1.06	0.437	-1.06	0.425	0.149
286	P07954	FUMH	Fumarate hydratase, mitochondrial	Mitochondria	2.3. Energy Metabolism - Tricarboxylic Acid Cycle	6.99	50.08	14.21	8	-1.09	0.188	1.08	0.218	-1.02	0.801	0.0869
287	P07954	FUMH	Fumarate hydratase, mitochondrial	Mitochondria	2.3. Energy Metabolism - Tricarboxylic Acid Cycle	6.99	50.08	45	9	-1.47	3.04E-05	-1.14	0.0273	-1.34	1.34E-03	3.07E-04
288	P60842	IF4A1	Eukaryotic initiation factor 4A-I	Cytoplasm	6. Transcription and Translation Processes	5.32	46.15	57*	4	-1.01	0.854	-1.05	0.724	1.01	0.983	0.892
289	P48735	IDHP	Isocitrate dehydrogenase [NADP], mitochondrial	Mitochondria	2.3. Energy Metabolism - Tricarboxylic Acid Cycle	8.32	46.61	11.31	13	-1.3	1.52E-04	-1.19	0.012	-1.32	7.78E-05	8.44E-05
290	P48735	IDHP	Isocitrate dehydrogenase [NADP], mitochondrial	Mitochondria	2.3. Energy Metabolism - Tricarboxylic Acid Cycle	8.32	46.61	44.27	13	-1.27	1.66E-03	-1.14	0.0438	-1.27	1.11E-03	3.34E-04
291	P17540	KCRS	Creatine kinase, sarcomeric mitochondrial	Mitochondria	2.5. Energy Metabolism - Creatine Kinase System (Energy Transduction)	7.28	43.34	5.76	7	-1.18	0.145	1.17	0.146	-1.12	0.29	3.40E-03
292	P17540	KCRS	Creatine kinase, sarcomeric mitochondrial	Mitochondria	2.5. Energy Metabolism - Creatine Kinase System (Energy Transduction)	7.28	43.34	56*	4	-1.27	0.0184	-1.13	0.116	-1.3	3.10E-03	0.0339
293	P17540	KCRS	Creatine kinase, sarcomeric mitochondrial	Mitochondria	2.5. Energy Metabolism - Creatine Kinase System (Energy Transduction)	7.28	43.34	13.25	14	-1.24	0.0142	-1.09	0.339	-1.14	0.0247	0.0326
294	P49411	EFTU	Elongation factor Tu, mitochondrial precursor (EF-Tu) (P43).	Mitochondria	6. Transcription and Translation Processes	6.31	45.05	11.21	8	-1.12	0.128	1.03	0.593	1.02	0.726	6.26E-03

295	P49411	EFTU	Elongation factor Tu, mitochondrial precursor (EF-Tu) (P43).	Mitochondria	6. Transcription and Translation Processes	6.31	45.05	15.47	14	-1.17	0.0216	-1.02	0.812	-1.09	0.254	0.143
296	P42765	THIM	3-ketoacyl-CoA thiolase, mitochondrial	Mitochondria	2.2. Energy Metabolism - Lipid Metabolism/ß-Oxidation	8.32	41.92	10.7	9	-1.29	6.39E-03	1.06	0.268	-1.13	0.117	8.74E-05
297	P42765	THIM	3-ketoacyl-CoA thiolase, mitochondrial	Mitochondria	2.2. Energy Metabolism - Lipid Metabolism/ß-Oxidation	8.32	41.92	35.68	8	-1.21	0.111	1.05	0.464	-1.21	0.0262	9.66E-03
298	NP	NP	NP - Spot not present in the preparative gel	NP	11. NP - Spot not present in the preparative gel	NP	NP	NP	NP	-1.19	0.0605	-1.08	0.453	-1.22	0.038	0.0319
299	P00558	PGK1	Phosphoglycerate kinase 1	Cytoplasm	2.1. Energy Metabolism - Glycolysis	8.30	44.48	25.56	8	-1.08	0.331	1.03	0.761	-1.12	0.165	0.6
300	P00558	PGK1	Phosphoglycerate kinase 1	Cytoplasm	2.1. Energy Metabolism - Glycolysis	8.30	44.48	10.47	7	-1.03	0.75	1.04	0.769	-1.09	0.311	0.799
301	NP	NP	NP - Spot not present in the preparative gel	NP	11. NP - Spot not present in the preparative gel	NP	NP	NP	NP	-1.2	0.0368	-1.01	0.922	-1.01	0.852	0.0365
302	P68032	ACTC	Actin, alpha cardiac muscle 1 (Alpha-cardiac actin)	Cytoskeleton	1. Structural and Contractile Proteins	5.23	41.78	8.57	6	-1.29	9.68E-04	-1.13	0.151	-1.18	0.0228	0.0368
303	O75390	CISY	Citrate synthase, mitochondrial	Mitochondria	2.3. Energy Metabolism - Tricarboxylic Acid Cycle	7.39	49.01	10.18	8	-1.08	0.357	1.04	0.549	1.01	0.872	0.364
304	O75390	CISY	Citrate synthase, mitochondrial	Mitochondria	2.3. Energy Metabolism - Tricarboxylic Acid Cycle	7.39	49.01	7.68	5	-1.17	0.0235	1.01	0.79	-1.04	0.58	0.219
305	P17540	KCRS	Creatine kinase, sarcomeric mitochondrial	Mitochondria	2.5. Energy Metabolism - Creatine Kinase System (Energy Transduction)	7.28	43.34	10.1	10	-1.11	0.199	1.06	0.415	-1.02	0.863	4.55E-03
306	P68032	ACTC	Actin, alpha cardiac muscle 1 (Alpha-cardiac actin)	Cytoskeleton	1. Structural and Contractile Proteins	5.23	41.78	35.68	5	-1.04	0.817	-1.05	0.719	-1.05	0.687	0.729
307	P68032	ACTC	Actin, alpha cardiac muscle 1 (Alpha-cardiac actin)	Cytoskeleton	1. Structural and Contractile Proteins	5.23	41.78	9.99	5	-1.06	0.387	-1.04	0.63	-1.05	0.507	0.161
308	P12277	KCRB	Creatine kinase B-type	Cytoplasm	2.5. Energy Metabolism - Creatine Kinase System (Energy Transduction)	5.35	42.51	7.43	7	-1.09	0.0739	1.12	0.0335	1.01	0.665	1.24E-04
309	NP	NP	NP - Spot not present in the preparative gel	NP	11. NP - Spot not present in the preparative gel	NP	NP	NP	NP	-1	0.835	1.05	0.13	-1.04	0.319	0.0595
310	P68032	ACTC	Actin, alpha cardiac muscle 1 (Alpha-cardiac actin)	Cytoskeleton	1. Structural and Contractile Proteins	5.23	41.78	11.55	6	-1.12	0.0734	1.03	0.558	-1.11	0.0434	2.13E-03
311	P68032	ACTC	Actin, alpha cardiac muscle 1 (Alpha-cardiac actin)	Cytoskeleton	1. Structural and Contractile Proteins	5.23	41.78	10.83	6	-1.3	1.30E-03	-1.01	0.821	-1.24	5.75E-04	3.29E-05
312	P68032	ACTC	Actin, alpha cardiac muscle 1 (Alpha-cardiac actin)	Cytoskeleton	1. Structural and Contractile Proteins	5.23	41.78	10.9	6	-1.33	1.21E-03	-1.04	0.574	-1.29	4.44E-04	2.04E-05
313	P08865	RSSA	40S ribosomal protein AS	Membrane	6. Transcription and Translation Processes	4.79	32.72	9.14	4	1.03	0.653	-1.05	0.698	-1.02	0.959	0.612
314	NP	NP	NP - Spot not present in the preparative gel	NP	11. NP - Spot not present in the preparative gel	NP	NP	NP	NP	-1.06	0.178	1.02	0.647	-1.07	0.126	0.11
315	NP	NP	NP - Spot not present in the preparative gel	NP	11. NP - Spot not present in the preparative gel	NP	NP	NP	NP	-1.07	0.0577	1.01	0.912	-1.12	0.0173	0.0244
316	NP	NP	NP - Spot not present in the preparative gel	NP	11. NP - Spot not present in the preparative gel	NP	NP	NP	NP	-1.24	3.22E-03	-1.05	0.425	-1.24	9.55E-04	2.21E-04
317	NP	NP	NP - Spot not present in the preparative gel	NP	11. NP - Spot not present in the preparative gel	NP	NP	NP	NP	-1.26	3.64E-03	-1.04	0.566	-1.22	2.93E-03	1.06E-04
318	P68032	ACTC	Actin, alpha cardiac muscle 1 (Alpha-cardiac actin)	Cytoskeleton	1. Structural and Contractile Proteins	5.23	41.78	10.47	6	-1.03	0.413	1.05	0.402	-1.06	0.185	0.103
319	P68032	ACTC	Actin, alpha cardiac muscle 1 (Alpha-cardiac actin)	Cytoskeleton	1. Structural and Contractile Proteins	5.23	41.78	10.74	6	-1.12	0.0321	1.01	0.87	-1.08	0.0635	0.0735
320	P68032	ACTC	Actin, alpha cardiac muscle 1 (Alpha-cardiac actin)	Cytoskeleton	1. Structural and Contractile Proteins	5.23	41.78	9.77	6	-1.35	2.17E-03	-1.05	0.567	-1.35	4.98E-04	2.70E-06
321	P68032	ACTC	Actin, alpha cardiac muscle 1 (Alpha-cardiac actin)	Cytoskeleton	1. Structural and Contractile Proteins	5.23	41.78	9.3	6	-1.32	2.34E-03	-1.06	0.41	-1.34	1.08E-04	1.38E-07
322	P68032	ACTC	Actin, alpha cardiac muscle 1 (Alpha-cardiac actin)	Cytoskeleton	1. Structural and Contractile Proteins	5.23	41.78	9.82	6	-1.29	5.26E-03	-1.03	0.724	-1.27	3.15E-03	2.23E-05
323	P68032	ACTC	Actin, alpha cardiac muscle 1 (Alpha-cardiac actin)	Cytoskeleton	1. Structural and Contractile Proteins	5.23	41.78	11.12	5	-1.21	9.34E-03	1.03	0.663	-1.14	0.0172	1.39E-05
324	P06732	KCRM	Creatine kinase M-type	Cytoplasm	2.5. Energy Metabolism - Creatine Kinase System (Energy Transduction)	6.77	43.10	12.87	9	-1.72	1.43E-04	-1.29	0.0155	-1.85	6.44E-07	7.60E-10
325	P06732	KCRM	Creatine kinase M-type	Cytoplasm	2.5. Energy Metabolism - Creatine Kinase System (Energy Transduction)	6.77	43.10	11.77	10	-1.8	4.20E-05	-1.26	0.0115	-1.9	4.42E-08	2.79E-10
326	P06732	KCRM	Creatine kinase M-type	Cytoplasm	2.5. Energy Metabolism - Creatine Kinase System (Energy Transduction)	6.77	43.10	11.23	12	-1.75	4.23E-05	-1.26	9.62E-03	-1.81	9.24E-08	3.69E-10
327	P06732	KCRM	Creatine kinase M-type	Cytoplasm	2.5. Energy Metabolism - Creatine Kinase System (Energy Transduction)	6.77	43.10	9.16	8	-1.48	3.56E-04	-1.13	0.097	-1.44	1.41E-05	0.0213
328	P68032	ACTC	Actin, alpha cardiac muscle 1 (Alpha-cardiac actin)	Cytoskeleton	1. Structural and Contractile Proteins	5.23	41.78	11.75	6	-1.04	0.412	-1.04	0.442	-1.08	0.0526	0.307
329	P04075	ALDOA	Fructose-bisphosphate aldolase A (Muscle-type aldolase)	Cytoplasm	2.1. Energy Metabolism - Glycolysis	8.39	39.29	7.33	10	-1.15	0.018	1.08	0.122	-1.16	0.0193	3.99E-04
330	P04075	ALDOA	Fructose-bisphosphate aldolase A (Muscle-type aldolase)	Cytoplasm	2.1. Energy Metabolism - Glycolysis	8.39	39.29	10.61	14	-1.14	0.0283	1.09	0.235	-1.18	0.023	3.78E-03
331	NI	NI	NI - Spot not identified by MS	NI	10. NI - Spot not identified by MS	NI	NI	NI	NI	-1.19	0.0298	1.09	0.0783	-1.13	0.0389	1.35E-03

332	P04075	ALDOA	Fructose-bisphosphate aldolase A (Muscle-type aldolase)	Cytoplasm	2.1. Energy Metabolism - Glycolysis	8.39	39.29	7.13	4	-1.24	2.97E-03	1.03	0.622	-1.25	1.06E-03	4.43E-05
333	P04075	ALDOA	Fructose-bisphosphate aldolase A (Muscle-type aldolase)	Cytoplasm	2.1. Energy Metabolism - Glycolysis	8.39	39.29	7.63	9	-1.15	0.0392	1.05	0.311	-1.18	0.0145	4.96E-04
334	P04075	ALDOA	Fructose-bisphosphate aldolase A (Muscle-type aldolase)	Cytoplasm	2.1. Energy Metabolism - Glycolysis	8.39	39.29	35.18	9	-1.18	0.0166	1.07	0.334	-1.16	0.0631	2.29E-03
335	P00505	AATM	Aspartate aminotransferase, mitochondrial	Mitochondria	2.6. Other Metabolic Processes	8.98	44.74	9.75	4	-1.08	0.237	1	0.951	1	0.985	0.441
336	P00505	AATM	Aspartate aminotransferase, mitochondrial	Mitochondria	2.6. Other Metabolic Processes	8.98	44.74	10.67	8	-1.07	0.264	1.04	0.395	1.03	0.616	0.271
337	P24752	THIL	Acetyl-CoA acetyltransferase, mitochondrial	Mitochondria	2.6. Other Metabolic Processes	8.16	41.39	72*	6	-1.28	1.36E-03	1.01	0.719	-1.21	2.50E-03	1.86E-04
338	P00505	AATM	Aspartate aminotransferase, mitochondrial	Mitochondria	2.6. Other Metabolic Processes	8.98	44.74	10.27	11	-1.06	0.253	1.08	0.187	1.07	0.411	0.446
339	P00505	AATM	Aspartate aminotransferase, mitochondrial	Mitochondria	2.6. Other Metabolic Processes	8.98	44.74	9.86	9	-1.09	0.0926	1.07	0.213	1.03	0.888	0.573
340	NP	NP	NP - Spot not present in the preparative gel	NP	11. NP - Spot not present in the preparative gel	NP	NP	NP	NP	-1.1	0.289	-1.02	0.828	-1.11	0.219	0.308
341	NP	NP	NP - Spot not present in the preparative gel	NP	11. NP - Spot not present in the preparative gel	NP	NP	NP	NP	-1	0.945	1.09	0.23	-1.04	0.504	0.306
342	P04075	ALDOA	Fructose-bisphosphate aldolase A (Muscle-type aldolase)	Cytoplasm	2.1. Energy Metabolism - Glycolysis	8.39	39.29	103*	7	-1.15	0.0734	-1.03	0.511	-1.2	2.83E-03	6.52E-03
343	Q15019	SEPT2	Septin-2	Cytoplasm	9. Other Functions	6.15	41.49	9.46	7	1.1	0.157	1.11	0.127	1.03	0.612	0.534
344	P09972	ALDOC	Fructose-bisphosphate aldolase C (Brain-type aldolase)	Cytoplasm	2.1. Energy Metabolism - Glycolysis	6.46	39.32	5.84	6	-1.31	1.30E-03	-1.06	0.319	-1.26	4.06E-03	3.26E-04
345	P45379	TNNT2	Troponin T, cardiac muscle	Cytoskeleton	1. Structural and Contractile Proteins	4.94	35.79	7.41	7	-1.2	0.0856	-1.08	0.419	-1.07	0.464	9.84E-03
346	Q9UJZ1	STML2	Stomatin-like protein 2	Membrane	1. Structural and Contractile Proteins	6.87	38.53	8.09	3	-1.42	4.23E-03	-1.11	0.283	-1.26	0.0605	0.0757
347	P45379	TNNT2	Troponin T, cardiac muscle	Cytoskeleton	1. Structural and Contractile Proteins	4.94	35.79	24.36	7	-1.15	0.312	1.21	0.652	-1.33	0.0225	0.0427
348	Q99471	PFD5	Prefoldin subunit 5	Cytoskeleton	1. Structural and Contractile Proteins	5.96	17.20	56*	7	-1.17	0.0856	-1.1	0.327	-1.04	0.581	0.0352
349	P00505	AATM	Aspartate aminotransferase, mitochondrial	Mitochondria	2.6. Other Metabolic Processes	8.98	44.74	6.83	4	-1.09	0.33	1.02	0.867	-1.06	0.495	0.144
350	P17174	AATC	Aspartate aminotransferase, cytoplasmic	Cytoplasm	2.6. Other Metabolic Processes	6.57	46.12	95*	6	-1.19	0.0306	-1.11	0.117	-1.16	0.0254	0.0145
351	P45379	TNNT2	Troponin T, cardiac muscle	Cytoskeleton	1. Structural and Contractile Proteins	4.94	35.79	6.98	5	-1.15	0.139	-1.06	0.472	1.03	0.68	0.0172
352	NI	NI	NI - Spot not identified by MS	NI	10. NI - Spot not identified by MS	NI	NI	NI	NI	-1.26	4.56E-03	-1.08	0.166	-1.25	2.42E-04	3.22E-05
353	P17174	AATC	Aspartate aminotransferase, cytoplasmic	Cytoplasm	2.6. Other Metabolic Processes	6.57	46.12	15.37	16	-1.4	1.59E-04	-1.22	1.99E-03	-1.35	2.55E-04	7.74E-06
354	P17174	AATC	Aspartate aminotransferase, cytoplasmic	Cytoplasm	2.6. Other Metabolic Processes	6.57	46.12	12.8	11	-1.37	2.12E-04	-1.24	3.64E-03	-1.4	1.83E-05	7.64E-07
355	P45379	TNNT2	Troponin T, cardiac muscle	Cytoskeleton	1. Structural and Contractile Proteins	4.94	35.79	10.7	7	-1.13	0.537	1.24	0.557	-1.36	0.0607	8.41E-03
356	NP	NP	NP - Spot not present in the preparative gel	NP	11. NP - Spot not present in the preparative gel	NP	NP	NP	NP	-1.17	0.101	-1.22	0.0463	-1.23	0.0158	1.25E-03
357	A8MPP1	DD11L	Putative ATP-dependent RNA helicase DDX11-like protein	Nucleus	2.6. Other Metabolic Processes	7.28	101.81	59*	7	-1.28	0.0188	-1.09	0.398	1.01	0.97	0.214
358	NI	NI	NI - Spot not identified by MS	NI	10. NI - Spot not identified by MS	NI	NI	NI	NI	-1.08	0.782	1.05	0.569	1.03	0.656	0.724
359	O76021	RL1D1	Ribosomal L1 domain-containing protein 1	Nucleus	6. Transcription and Translation Processes	10.13	54.97	60*	7	-1.29	0.233	2.1	0.0447	1.09	0.4	0.0146
360	O95299	NDUAA	NADH dehydrogenase [ubiquinone] 1 alpha subcomplex subunit 10	Mitochondria	2.4. Energy Metabolism - Oxidative Phosphorylation and Electron Transport	6.87	37.15	7.9	10	-1.28	9.14E-04	-1.07	0.121	-1.29	1.19E-04	6.17E-05
361	P58499	FAM3B	Protein FAM3B	Secreted	9. Other Functions	8.77	22.85	58*	6	-1.11	0.151	1.06	0.547	-1.02	0.762	9.76E-03
362	Q92618	ZN516	Zinc finger protein 516	Nucleus	6. Transcription and Translation Processes	9.02	124.29	50*	8	-1.07	0.404	1.12	0.199	1.03	0.627	0.0723
363	Q15056	IF4H	Eukaryotic translation initiation factor 4H	Cytoplasm	6. Transcription and Translation Processes	6.92	27.25	50*	6	-1.33	3.35E-04	-1.05	0.417	-1.27	1.88E-03	1.13E-03
364	P63218	GBG5	Guanine nucleotide-binding protein G(I)/G(S)/G(O) subunit gamma-5	Membrane	5. Cell Signaling	9.90	6.84	38		-1.87	0.0663	-1.38	0.262	-2.02	0.0272	0.0403
365	P63218	GBG5	Guanine nucleotide-binding protein G(I)/G(S)/G(O) subunit gamma-5	Membrane	5. Cell Signaling	9.90	6.84	40		-1.37	0.073	-1.57	0.0211	-1.69	3.26E-03	0.0196
366	Q68D91	MBLC2	Metallo-beta-lactamase domain-containing protein 2	Cytoplasm	9. Other Functions	6.41	31.37	49		1.23	0.0637	-1.01	0.97	1.15	0.102	2.13E-03
367	NP	NP	NP - Spot not present in the preparative gel	NP	11. NP - Spot not present in the preparative gel	NP	NP	NP	NP	-1.05	0.645	-1.06	0.574	-1.18	0.0698	0.142
368	NP	NP	NP - Spot not present in the preparative gel	NP	11. NP - Spot not present in the preparative gel	NP	NP	NP	NP	1.05	0.452	1.01	0.748	-1.03	0.68	0.62

369	NI	NI	NI - Spot not identified by MS	NI	10. NI - Spot not identified by MS	NI	NI	NI	NI	1.04	0.579	1.12	0.499	-1.1	0.504	0.488
370	P16219	ACADS	Short-chain specific acyl-CoA dehydrogenase, mitochondrial	Mitochondria	2.2. Energy Metabolism - Lipid Metabolism/ß-Oxidation	6.15	41.72	54*	4	-1.15	0.217	1.04	0.877	-1.12	0.259	0.663
371	P45379	TNNT2	Troponin T, cardiac muscle	Cytoskeleton	1. Structural and Contractile Proteins	4.94	35.79	6.65	5	-1.37	0.114	1.52	0.0194	1.17	0.15	3.36E-04
372	NP	NP	NP - Spot not present in the preparative gel	NP	11. NP - Spot not present in the preparative gel	NP	NP	NP	NP	-1.03	0.92	-1.11	0.467	1.66	1.07E-03	5.72E-03
373	P50213	IDH3A	Isocitrate dehydrogenase [NAD] subunit alpha, mitochondrial	Mitochondria	2.3. Energy Metabolism - Tricarboxylic Acid Cycle	5.71	36.64	9.8	5	1.15	6.02E-03	1.18	0.0372	1.24	7.66E-03	0.0129
374	NI	NI	NI - Spot not identified by MS	NI	10. NI - Spot not identified by MS	NI	NI	NI	NI	1.29	1.21E-03	-1.02	0.665	1.06	0.426	0.0132
375	NI	NI	NI - Spot not identified by MS	NI	10. NI - Spot not identified by MS	NI	NI	NI	NI	-1.17	0.0553	-1.03	0.687	-1.16	0.0461	0.0112
376	NI	NI	NI - Spot not identified by MS	NI	10. NI - Spot not identified by MS	NI	NI	NI	NI	1.16	0.381	1.01	0.892	1.01	0.84	0.883
377	P50213	IDH3A	Isocitrate dehydrogenase [NAD] subunit alpha, mitochondrial	Mitochondria	2.3. Energy Metabolism - Tricarboxylic Acid Cycle	5.71	36.64	10.73	4	1.2	4.69E-03	1.12	0.321	1.22	0.0146	0.0525
378	O00151	PDLI1	PDZ and LIM domain protein 1	Cytoskeleton	1. Structural and Contractile Proteins	6.55	35.94	6.66	5	1.15	0.316	1.06	0.829	1.3	5.93E-04	2.57E-03
379	NI	NI	NI - Spot not identified by MS	NI	10. NI - Spot not identified by MS	NI	NI	NI	NI	-1.25	0.0136	-1.07	0.409	-1.21	0.0478	0.0303
380	NP	NP	NP - Spot not present in the preparative gel	NP	11. NP - Spot not present in the preparative gel	NP	NP	NP	NP	2.34	4.90E-05	1.26	4.70E-03	1.61	4.21E-05	2.67E-06
381	P02689	MYP2	Myelin P2	Cytoplasm	7. Transport and Transmembrane Transport	9.84	14.78	57*	4	-1.21	0.0292	1.13	0.263	-1.02	0.77	0.0844
382	P04083	ANXA1	Annexin A1 (Annexin I)	Nucleus	3. Stress Response and Apoptosis	6.64	38.58	10.25	5	2.35	2.66E-06	1.14	0.06	1.56	1.76E-04	1.52E-07
383	P40926	MDHM	Malate dehydrogenase, mitochondrial	Mitochondria	2.3. Energy Metabolism - Tricarboxylic Acid Cycle	8.54	33.00	7.67	6	-1.22	0.0712	1.04	0.643	-1.1	0.295	0.0631
384	P04406	G3P	Glyceraldehyde-3-phosphate dehydrogenase (GAPDH)	Cytoplasm	2.1. Energy Metabolism - Glycolysis	8.58	35.92	8.72	6	-1	0.993	1.14	0.178	-1.05	0.516	0.317
385	P62140	PP1B	Serine/threonine-protein phosphatase PP1-beta catalytic subunit	Cytoplasm	9. Other Functions	5.85	37.06	7.26	5	1.05	0.443	1.08	0.151	1.1	0.0738	0.561
386	P07355	ANXA2	Annexin A2 (Annexin II)	Secreted	9. Other Functions	7.56	38.47	15.51	14	1.7	3.42E-04	1.23	0.0581	1.26	0.0464	6.03E-03
387	P07355	ANXA2	Annexin A2 (Annexin II)	Secreted	9. Other Functions	7.56	38.47	108*	6	1.7	8.78E-05	1.17	0.0663	1.29	5.49E-03	3.74E-03
388	Q14894	CRYM	Mu-crystallin homolog	Cytoplasm	2.6. Other Metabolic Processes	5.06	33.78	27.47	5	1.21	0.115	1.14	0.0151	1.27	0.0693	2.75E-03
389	P04406	G3P	Glyceraldehyde-3-phosphate dehydrogenase (GAPDH)	Cytoplasm	2.1. Energy Metabolism - Glycolysis	8.58	35.92	7.4	3	1.23	2.00E-03	1.16	0.0586	1.2	0.0313	3.84E-03
390	P04406	G3P	Glyceraldehyde-3-phosphate dehydrogenase (GAPDH)	Cytoplasm	2.1. Energy Metabolism - Glycolysis	8.58	35.92	8.07	3	1.27	3.69E-03	1.19	0.0254	1.12	0.0911	2.11E-03
391	P04406	G3P	Glyceraldehyde-3-phosphate dehydrogenase (GAPDH)	Cytoplasm	2.1. Energy Metabolism - Glycolysis	8.58	35.92	7.45	3	1.04	0.601	1.13	0.168	-1.01	0.932	0.205
392	P47755	CAZA2	F-actin capping protein subunit alpha-2 (CapZ alpha-2)	Cytoskeleton	1. Structural and Contractile Proteins	5.58	32.82	14.21	7	-1.1	0.069	-1.02	0.678	-1.12	8.57E-03	0.0152
393	Q6P3V2	Z585A	Zinc finger protein 585A	Nucleus	6. Transcription and Translation Processes	9.26	87.97	53*	7	1.06	0.641	1.04	0.798	1.14	0.191	5.05E-04
394	NI	NI	NI - Spot not identified by MS	NI	10. NI - Spot not identified by MS	NI	NI	NI	NI	1.48	2.32E-05	1.25	2.99E-03	1.28	4.59E-04	3.80E-04
395	P04406	G3P	Glyceraldehyde-3-phosphate dehydrogenase (GAPDH)	Cytoplasm	2.1. Energy Metabolism - Glycolysis	8.58	35.92	7.15	4	1.09	0.25	1.14	0.0896	1.03	0.616	3.93E-03
396	P04406	G3P	Glyceraldehyde-3-phosphate dehydrogenase (GAPDH)	Cytoplasm	2.1. Energy Metabolism - Glycolysis	8.58	35.92	58*	5	1.13	0.0673	1.15	0.0929	1.02	0.722	0.018
397	P04406	G3P	Glyceraldehyde-3-phosphate dehydrogenase (GAPDH)	Cytoplasm	2.1. Energy Metabolism - Glycolysis	8.58	35.92	27.47	5	1	0.938	1.08	0.485	-1.05	0.446	0.246
398	NI	NI	NI - Spot not identified by MS	NI	10. NI - Spot not identified by MS	NI	NI	NI	NI	1.19	9.56E-03	1.16	0.0219	1.1	0.104	1.75E-03
399	Q96L73	NSD1	Histone-lysine N-methyltransferase, H3 lysine-36 and H4 lysine-20 specific	Nucleus	6. Transcription and Translation Processes	8.40	296.65	57*	9	1.1	0.264	1.17	0.108	1.03	0.747	0.31
400	NP	NP	NP - Spot not present in the preparative gel	NP	11. NP - Spot not present in the preparative gel	NP	NP	NP	NP	-1.21	3.07E-03	-1.06	0.364	-1.15	0.0445	4.02E-03
401	NP	NP	NP - Spot not present in the preparative gel	NP	11. NP - Spot not present in the preparative gel	NP	NP	NP	NP	1.1	0.389	1.13	0.291	1.08	0.286	0.413
402	NI	NI	NI - Spot not identified by MS	NI	10. NI - Spot not identified by MS	NI	NI	NI	NI	-1.09	0.241	1.06	0.715	-1.05	0.494	0.113
403	NI	NI	NI - Spot not identified by MS	NI	10. NI - Spot not identified by MS	NI	NI	NI	NI	1.02	0.739	-1.03	0.611	-1.02	0.79	0.303
404	NI	NI	NI - Spot not identified by MS	NI	10. NI - Spot not identified by MS	NI	NI	NI	NI	1.03	0.5	1.02	0.661	1.14	0.0171	0.0187
405	Q9Y2K2	QSK	Serine/threonine-protein kinase QSK	Cytoplasm	9. Other Functions	6.22	139.98	56*	7	-1.27	0.506	-1.21	0.649	-1.02	0.894	0.759

406	P45379	TNNT2	Troponin T, cardiac muscle	Cytoskeleton	1. Structural and Contractile Proteins	4.94	35.79	64*	4	-1.65	1.92E-03	-1.39	0.0668	-1.58	7.45E-03	5.09E-04
407	NI	NI	NI - Spot not identified by MS	NI	10. NI - Spot not identified by MS	NI	NI	NI	NI	-1.31	0.0637	-1.32	0.0476	-1.16	0.223	4.45E-04
408	NP	NP	NP - Spot not present in the preparative gel	NP	11. NP - Spot not present in the preparative gel	NP	NP	NP	NP	1.37	0.0479	1.02	0.685	1.14	0.167	0.0902
409	P07355	ANXA2	Annexin A2 (Annexin II)	Secreted	9. Other Functions	7.56	38.47	51*	3	1.62	1.78E-03	1.16	0.0335	1.35	1.76E-03	3.00E-03
410	P22626	ROA2	Heterogeneous nuclear ribonucleoproteins A2/B1	Nucleus	2.6. Other Metabolic Processes	8.97	37.43	7.52	4	-1.27	2.47E-03	-1.09	0.232	-1.17	0.0337	3.11E-03
411	Q9UBT7	CTNL1	Alpha-catulin	Cytoskeleton	1. Structural and Contractile Proteins	6.22	81.90	53*	6	-1.05	0.467	-1.06	0.398	1.01	0.819	0.557
412	NP	NP	NP - Spot not present in the preparative gel	NP	11. NP - Spot not present in the preparative gel	NP	NP	NP	NP	-1.02	0.854	-1.1	0.266	1.01	0.84	0.233
413	NP	NP	NP - Spot not present in the preparative gel	NP	11. NP - Spot not present in the preparative gel	NP	NP	NP	NP	-1.22	2.11E-03	1.09	0.291	-1.12	0.0932	0.0496
414	NI	NI	NI - Spot not identified by MS	NI	10. NI - Spot not identified by MS	NI	NI	NI	NI	-1.17	0.0813	-1.14	0.0733	-1.22	0.0178	0.0564
415	P11177	ODPB	Pyruvate dehydrogenase E1 component subunit beta, mitochondrial	Mitochondria	2.1. Energy Metabolism - Glycolysis	5.38	35.90	59*	6	-1.12	0.0464	1.01	0.909	-1.07	0.134	0.177
416	P40926	MDHM	Malate dehydrogenase, mitochondrial	Mitochondria	2.3. Energy Metabolism - Tricarboxylic Acid Cycle	8.54	33.00	10.62	9	-1.28	3.27E-03	1.02	0.763	-1.14	0.107	8.55E-03
417	P40926	MDHM	Malate dehydrogenase, mitochondrial	Mitochondria	2.3. Energy Metabolism - Tricarboxylic Acid Cycle	8.54	33.00	10.03	6	-1.22	5.87E-03	1.05	0.464	-1.1	0.195	0.0169
418	P11177	ODPB	Pyruvate dehydrogenase E1 component subunit beta, mitochondrial	Mitochondria	2.1. Energy Metabolism - Glycolysis	5.38	35.90	57*	6	-1.1	0.0645	-1.01	0.931	-1.05	0.343	0.333
419	P40926	MDHM	Malate dehydrogenase, mitochondrial	Mitochondria	2.3. Energy Metabolism - Tricarboxylic Acid Cycle	8.54	33.00	12.3	9	-1.23	9.22E-03	-1.02	0.763	-1.16	0.0173	5.75E-03
420	A6NNV3	W19L5	Putative WBSCR19-like protein 5	Other	9. Other Functions	8.38	37.50	58*	4	-1.15	0.238	-1.4	7.02E-03	-1.02	0.694	1.48E-04
421	P22626	ROA2	Heterogeneous nuclear ribonucleoproteins A2/B1	Nucleus	2.6. Other Metabolic Processes	8.97	37.43	8.12	4	-1.23	0.0127	1.02	0.758	-1.16	0.021	1.55E-03
422	P45379	TNNT2	Troponin T, cardiac muscle	Cytoskeleton	1. Structural and Contractile Proteins	4.94	35.79	59*	4	-1.48	0.0391	-1.63	0.0319	-1.36	0.101	3.91E-04
423	NP	NP	NP - Spot not present in the preparative gel	NP	11. NP - Spot not present in the preparative gel	NP	NP	NP	NP	-1.22	0.0223	1.08	0.334	-1.12	0.0924	3.61E-04
424	P23276	KELL	Kell blood group glycoprotein	Membrane	9. Other Functions	8.09	82.82	54*	7	-1.21	2.11E-03	-1.04	0.426	-1.19	0.0108	1.26E-03
425	P40925	MDHC	Malate dehydrogenase, cytoplasmic	Cytoplasm	2.6. Other Metabolic Processes	6.89	36.29	7.65	6	-1.31	1.26E-03	-1.09	0.205	-1.26	7.53E-03	1.67E-03
426	P40925	MDHC	Malate dehydrogenase, cytoplasmic	Cytoplasm	2.6. Other Metabolic Processes	6.89	36.29	50*	3	-1.07	0.258	1.02	0.8	1	0.966	0.372
427	P21796	VDAC1	Voltage-dependent anion-selective channel protein 1	Mitochondria	7. Transport and Transmembrane Transport	8.63	30.64	9.3	6	-1.06	0.4	1.11	0.163	1.07	0.365	0.327
428	NP	NP	NP - Spot not present in the preparative gel	NP	11. NP - Spot not present in the preparative gel	NP	NP	NP	NP	-1.38	0.0469	-1.19	0.248	-1.32	0.0631	0.0121
429	Q16836	HCDH	Hydroxyacyl-coenzyme A dehydrogenase, mitochondrial	Mitochondria	2.2. Energy Metabolism - Lipid Metabolism/ß-Oxidation	8.38	32.84	54*	3	-1.31	1.19E-03	1.04	0.59	-1.14	0.0604	1.18E-03
430	Q16836	HCDH	Hydroxyacyl-coenzyme A dehydrogenase, mitochondrial	Mitochondria	2.2. Energy Metabolism - Lipid Metabolism/ß-Oxidation	8.38	32.84	64*	4	-1.28	2.73E-03	1.08	0.389	-1.05	0.457	3.11E-03
431	NP	NP	NP - Spot not present in the preparative gel	NP	11. NP - Spot not present in the preparative gel	NP	NP	NP	NP	-1.15	0.312	-1.07	0.679	1.05	0.66	0.589
432	NI	NI	NI - Spot not identified by MS	NI	10. NI - Spot not identified by MS	NI	NI	NI	NI	-1.17	0.0251	-1.02	0.69	-1.14	0.0266	0.0989
433	Q16836	HCDH	Hydroxyacyl-coenzyme A dehydrogenase, mitochondrial	Mitochondria	2.2. Energy Metabolism - Lipid Metabolism/ß-Oxidation	8.38	32.84	69*	5	-1.25	2.10E-03	-1.01	0.988	-1.04	0.522	8.70E-04
434	Q16352	AINX	Alpha-internexin	Cytoplasm	1. Structural and Contractile Proteins	5.34	55.39	52*	5	-1.32	2.47E-03	-1.02	0.734	-1.36	1.52E-05	9.27E-05
435	Q9NPC6	MYOZ2	Myozenin-2	Cytoskeleton	1. Structural and Contractile Proteins	7.86	29.90	6.93	5	-1.16	0.0463	1.04	0.483	-1.19	2.42E-03	1.69E-03
436	NP	NP	NP - Spot not present in the preparative gel	NP	11. NP - Spot not present in the preparative gel	NP	NP	NP	NP	-1.19	0.161	-1.02	0.745	-1.16	0.129	0.42
437	O75208	COQ9	Ubiquinone biosynthesis protein COQ9, mitochondrial	Mitochondria	2.6. Other Metabolic Processes	5.01	30.94	51*	3	-1.09	0.137	1.01	0.923	1.22	3.16E-03	1.56E-09
438	Q6WCQ1	MPRIP	Myosin phosphatase Rho-interacting protein	Cytoskeleton	1. Structural and Contractile Proteins	5.89	116.40	53*	5	-1.05	0.493	-1.02	0.795	1.05	0.458	7.50E-06
439	NP	NP	NP - Spot not present in the preparative gel	NP	11. NP - Spot not present in the preparative gel	NP	NP	NP	NP	1.21	0.0654	1.07	0.393	1.1	0.545	0.239
440	Q9ULU4	PKCB1	Protein kinase C-binding protein 1	Cytoplasm	5. Cell Signaling	6.83	131.69	56*	7	-1.15	0.0967	1.02	0.691	-1.1	0.275	7.96E-03
441	NI	NI	NI - Spot not identified by MS	NI	10. NI - Spot not identified by MS	NI	NI	NI	NI	-1.15	0.0583	1.07	0.23	-1.05	0.385	4.78E-05
442	Q6Q0C0	TRAF7	E3 ubiquitin-protein ligase TRAF7	Cytoplasm	8. Proteasome-Ubiquitin Process	6.77	74.61	53*	7	-1.1	0.17	1	0.881	-1.06	0.413	0.649

443	NP	NP	NP - Spot not present in the preparative gel	NP	11. NP - Spot not present in the preparative gel	NP	NP	NP	NP	-1.08	0.714	-1.14	0.448	1.02	0.81	0.866
444	NP	NP	NP - Spot not present in the preparative gel	NP	11. NP - Spot not present in the preparative gel	NP	NP	NP	NP	-1.18	0.273	1.08	0.727	-1.04	0.934	0.0637
445	NI	NI	NI - Spot not identified by MS	NI	10. NI - Spot not identified by MS	NI	NI	NI	NI	-1.36	2.14E-03	1.03	0.7	-1.26	3.29E-03	1.53E-05
446	NI	NI	NI - Spot not identified by MS	NI	10. NI - Spot not identified by MS	NI	NI	NI	NI	1.16	0.0282	1.18	0.0805	1.14	0.0557	0.243
447	Q16836	HCDH	Hydroxyacyl-coenzyme A dehydrogenase, mitochondrial	Mitochondria	2.2. Energy Metabolism - Lipid Metabolism/ß-Oxidation	8.38	32.84	56*	4	-1.47	2.04E-03	1.04	0.61	-1.27	0.0268	2.39E-04
448	P02144	MYG	Myoglobin	Cytoplasm	9. Other Functions	7.29	17.05	66*	4	-1.18	0.0923	1.09	0.301	-1.08	0.351	0.0264
449	Q9NPC6	MYOZ2	Myozenin-2	Cytoskeleton	1. Structural and Contractile Proteins	7.86	29.90	68*	6	-1.12	0.228	1.08	0.355	1.01	0.883	0.108
450	NP	NP	NP - Spot not present in the preparative gel	NP	11. NP - Spot not present in the preparative gel	NP	NP	NP	NP	-1.46	0.0141	-1.05	0.661	-1.27	0.0251	7.95E-03
451	Q9NPC6	MYOZ2	Myozenin-2	Cytoskeleton	1. Structural and Contractile Proteins	7.86	29.90	61*	5	-1.21	0.0579	1.1	0.18	1.05	0.355	4.97E-03
452	NP	NP	NP - Spot not present in the preparative gel	NP	11. NP - Spot not present in the preparative gel	NP	NP	NP	NP	-1.02	0.741	1.22	3.52E-03	1.09	0.228	3.31E-04
453	Q16698	DECR	2,4-dienoyl-CoA reductase, mitochondrial	Mitochondria	2.2. Energy Metabolism - Lipid Metabolism/ß-Oxidation	8.79	32.15	6.61	5	-1.27	0.0152	1.07	0.501	-1.26	4.59E-03	1.76E-03
454	Q16698	DECR	2,4-dienoyl-CoA reductase, mitochondrial	Mitochondria	2.2. Energy Metabolism - Lipid Metabolism/ß-Oxidation	8.79	32.15	25.21	3	-1.47	3.83E-04	-1.01	0.906	-1.34	2.52E-03	1.11E-03
455	NP	NP	NP - Spot not present in the preparative gel	NP	11. NP - Spot not present in the preparative gel	NP	NP	NP	NP	-1.24	3.81E-03	-1.02	0.679	-1.24	1.71E-03	5.69E-04
456	P08758	ANXA5	Annexin A5 (Annexin V)	Cytoplasm	3. Stress Response and Apoptosis	4.94	35.81	61*	6	2.49	1.58E-05	1.42	0.0134	1.83	5.08E-05	1.03E-07
457	P08758	ANXA5	Annexin A5 (Annexin V)	Cytoplasm	3. Stress Response and Apoptosis	4.94	35.81	10.98	5	2.53	5.71E-05	1.43	0.0299	1.88	7.73E-05	7.30E-07
458	NI	NI	NI - Spot not identified by MS	NI	10. NI - Spot not identified by MS	NI	NI	NI	NI	1.15	0.0607	1.12	0.203	1.11	0.136	0.261
459	NP	NP	NP - Spot not present in the preparative gel	NP	11. NP - Spot not present in the preparative gel	NP	NP	NP	NP	-1.14	0.0372	1.26	2.05E-03	1.01	0.978	1.11E-03
460	NP	NP	NP - Spot not present in the preparative gel	NP	11. NP - Spot not present in the preparative gel	NP	NP	NP	NP	1.3	0.147	1.06	0.43	1.25	0.0157	0.0229
461	P51451	BLK	Tyrosine-protein kinase BLK	Cytoplasm	5. Cell Signaling	8.02	57.58	58*	9	1.86	1.40E-03	-1.27	0.132	1.1	0.514	6.14E-05
462	NI	NI	NI - Spot not identified by MS	NI	10. NI - Spot not identified by MS	NI	NI	NI	NI	-1.43	5.41E-03	-1.07	0.573	-1.25	0.0381	0.0118
463	P31751	AKT2	RAC-beta serine/threonine-protein kinase	Cytoplasm	3. Stress Response and Apoptosis	5.98	55.77	52*	7	-4.4	1.44E-03	-2.62	0.0172	-3.67	3.10E-03	2.31E-04
464	NI	NI	NI - Spot not identified by MS	NI	10. NI - Spot not identified by MS	NI	NI	NI	NI	-1.06	0.303	1.1	0.494	-1.09	0.0746	0.466
465	Q9Y5T4	DJC15	DnaJ homolog subfamily C member 15	Membrane	3. Stress Response and Apoptosis	10.08	16.38	58*	4	-1.12	0.152	1.31	0.0406	-1.05	0.502	0.0438
466	Q13011	ECH1	Delta(3,5)-Delta(2,4)-dienoyl-CoA isomerase, mitochondrial	Mitochondria	2.2. Energy Metabolism - Lipid Metabolism/ß-Oxidation	5.99	32.21	9.06	4	-1.09	0.321	1.12	0.421	-1.07	0.373	0.255
467	Q13011	ECH1	Delta(3,5)-Delta(2,4)-dienoyl-CoA isomerase, mitochondrial	Mitochondria	2.2. Energy Metabolism - Lipid Metabolism/ß-Oxidation	5.99	32.21	12	5	1.19	0.57	1.27	0.753	1.77	0.102	0.0377
468	P62258	1433E	14-3-3 protein epsilon	Cytoplasm	5. Cell Signaling	4.63	29.17	26.88	5	1.32	0.0152	1.04	0.544	1.27	0.0112	7.80E-05
469	P25786	PSA1	Proteasome subunit alpha type-1	Cytoplasm	8. Proteasome-Ubiquitin Process	6.15	29.56	62*	4	1.14	0.0449	-1	0.941	1.05	0.269	0.0246
470	P07339	CATD	Cathepsin D	Cytoplasm	2.6. Other Metabolic Processes	5.60	37.85	58*	4	1.41	4.12E-04	1.27	3.25E-03	1.59	1.76E-03	1.88E-03
471	NI	NI	NI - Spot not identified by MS	NI	10. NI - Spot not identified by MS	NI	NI	NI	NI	1.15	0.121	1.33	1.17E-03	1.62	6.55E-04	1.72E-04
472	P49588	SYAC	Alanyl-tRNA synthetase, cytoplasmic	Cytoplasm	6. Transcription and Translation Processes	5.34	106.81	22.09	5	1.66	4.42E-04	-1.05	0.642	1.01	0.814	6.02E-05
473	P17931	LEG3	Galectin-3	Nucleus	4. Immune Response	8.60	26.02	8.17	4	1.37	7.41E-04	1.2	0.0212	1.14	0.18	0.0862
474	P07339	CATD	Cathepsin D	Cytoplasm	2.6. Other Metabolic Processes	5.60	37.85	58*	4	1.19	0.0861	1.15	0.0976	1.27	0.012	0.134
475	NI	NI	NI - Spot not identified by MS	NI	10. NI - Spot not identified by MS	NI	NI	NI	NI	1.23	0.0793	1.06	0.566	1.27	0.0838	8.93E-03
476	NI	NI	NI - Spot not identified by MS	NI	10. NI - Spot not identified by MS	NI	NI	NI	NI	1.09	0.0614	1.1	0.0614	1.2	0.0132	0.0834
477	P61981	1433G	14-3-3 protein gamma	Cytoplasm	5. Cell Signaling	4.80	28.30	85*	6	1.24	0.0126	1.08	0.314	1.18	0.0388	0.0196
478	NP	NP	NP - Spot not present in the preparative gel	NP	11. NP - Spot not present in the preparative gel	NP	NP	NP	NP	1.25	0.035	1.09	0.397	-1.01	0.916	9.68E-04
479	Q06323	PSME1	Proteasome activator complex subunit 1 (Interferon gamma up-regulated I-5111 protein)	Cytoplasm	8. Proteasome-Ubiquitin Process	5.78	28.72	8.55	3	1.67	2.06E-08	-1.07	0.118	1.02	0.682	3.60E-07

480	NP	NP	NP - Spot not present in the preparative gel	NP	11. NP - Spot not present in the preparative gel	NP	NP	NP	NP	-1.32	0.0279	-1.48	4.36E-05	1.02	0.847	3.48E-05
481	NI	NI	NI - Spot not identified by MS	NI	10. NI - Spot not identified by MS	NI	NI	NI	NI	2.17	1.77E-04	1.28	1.35E-03	1.49	1.27E-05	3.31E-06
482	NI	NI	NI - Spot not identified by MS	NI	10. NI - Spot not identified by MS	NI	NI	NI	NI	1.24	0.0182	1.04	0.577	1.04	0.752	0.0644
483	NI	NI	NI - Spot not identified by MS	NI	10. NI - Spot not identified by MS	NI	NI	NI	NI	-1.05	0.511	1.14	0.041	1	0.871	0.0117
484	NI	NI	NI - Spot not identified by MS	NI	10. NI - Spot not identified by MS	NI	NI	NI	NI	1.71	1.20E-03	1.12	0.133	1.41	3.07E-04	5.37E-06
485	P00915	CAH1	Carbonic anhydrase 1	Cytoplasm	2.6. Other Metabolic Processes	6.63	28.74	8.24	5	1.85	0.0546	1.22	0.107	1.06	0.559	0.0403
486	P63104	1433Z	14-3-3 protein zeta/delta	Cytoplasm	5. Cell Signaling	4.73	27.75	7.73	5	1.64	9.29E-04	1.05	0.426	1.36	3.78E-03	1.70E-05
487	NI	NI	NI - Spot not identified by MS	NI	10. NI - Spot not identified by MS	NI	NI	NI	NI	-1.28	0.246	-1.47	0.075	-1.42	0.0985	0.0458
488	NI	NI	NI - Spot not identified by MS	NI	10. NI - Spot not identified by MS	NI	NI	NI	NI	4.95	8.90E-03	1.36	0.0122	1.95	9.36E-03	2.15E-03
489	NI	NI	NI - Spot not identified by MS	NI	10. NI - Spot not identified by MS	NI	NI	NI	NI	1.2	0.234	-1.04	0.906	-1.02	0.962	0.284
490	NI	NI	NI - Spot not identified by MS	NI	10. NI - Spot not identified by MS	NI	NI	NI	NI	-1.13	0.397	-1.17	0.255	-1.11	0.397	0.0481
491	NP	NP	NP - Spot not present in the preparative gel	NP	11. NP - Spot not present in the preparative gel	NP	NP	NP	NP	1.19	0.323	-1.17	0.168	1.03	0.951	0.117
492	NI	NI	NI - Spot not identified by MS	NI	10. NI - Spot not identified by MS	NI	NI	NI	NI	-1.11	0.153	-1.14	0.0579	-1.16	0.0544	0.101
493	NI	NI	NI - Spot not identified by MS	NI	10. NI - Spot not identified by MS	NI	NI	NI	NI	1.55	1.91E-04	1.04	0.471	1.26	1.63E-03	1.13E-06
494	Q96P53	WDFY2	WD repeat and FYVE domain-containing protein 2	Nucleus	9. Other Functions	6.46	45.15	56*	4	-1.32	1.24E-04	-1.11	0.0295	-1.38	3.89E-07	4.90E-08
495	P55851	UCP2	Mitochondrial uncoupling protein 2	Mitochondria	7. Transport and Transmembrane Transport	9.74	33.23	46*	5	-1.9	4.23E-04	-1.38	0.0668	-2.3	1.64E-05	1.80E-04
496	NP	NP	NP - Spot not present in the preparative gel	NP	11. NP - Spot not present in the preparative gel	NP	NP	NP	NP	1.21	0.0665	1.13	0.287	1.13	0.11	0.369
497	P15259	PGAM2	Phosphoglycerate mutase 2	Cytoplasm	2.1. Energy Metabolism - Glycolysis	9.00	28.63	7.82	9	-1.79	5.32E-06	-1.26	0.0277	-1.89	7.14E-08	6.31E-10
498	P15259	PGAM2	Phosphoglycerate mutase 2	Cytoplasm	2.1. Energy Metabolism - Glycolysis	9.00	28.63	71*	4	-1.65	1.12E-05	-1.19	0.0648	-1.69	1.06E-07	8.37E-09
499	P19429	TNNI3	Troponin I, cardiac muscle	Cytoskeleton	1. Structural and Contractile Proteins	9.87	23.88	59*	5	1.33	8.55E-03	1.41	1.94E-03	1.44	6.31E-04	6.69E-05
500	NI	NI	NI - Spot not identified by MS	NI	10. NI - Spot not identified by MS	NI	NI	NI	NI	-1.72	0.0482	-1.16	0.651	1.06	0.573	3.81E-03
501	NI	NI	NI - Spot not identified by MS	NI	10. NI - Spot not identified by MS	NI	NI	NI	NI	1.03	0.668	-1.02	0.668	-1.07	0.164	0.604
502	P38117	ETFB	Electron transfer flavoprotein subunit beta	Mitochondria	2.4. Energy Metabolism - Oxidative Phosphorylation and Electron Transport	8.29	27.71	61*	4	1.16	0.0877	1.18	0.0737	1.11	0.2	0.0684
503	NI	NI	NI - Spot not identified by MS	NI	10. NI - Spot not identified by MS	NI	NI	NI	NI	1.45	8.59E-03	1.76	4.50E-03	1.99	1.37E-05	2.97E-04
504	NI	NI	NI - Spot not identified by MS	NI	10. NI - Spot not identified by MS	NI	NI	NI	NI	-2.03	2.08E-03	-1.57	0.0591	-1.39	0.147	0.0103
505	P18669	PGAM1	Phosphoglycerate mutase 1	Cytoplasm	2.1. Energy Metabolism - Glycolysis	6.75	28.67	10.92	8	1.33	5.90E-05	1.2	4.66E-03	1.27	3.16E-03	6.31E-05
506	NP	NP	NP - Spot not present in the preparative gel	NP	11. NP - Spot not present in the preparative gel	NP	NP	NP	NP	-1.05	0.545	-1.04	0.636	-1.05	0.579	0.355
507	P18669	PGAM1	Phosphoglycerate mutase 1	Cytoplasm	2.1. Energy Metabolism - Glycolysis	6.75	28.67	80*	4	1.22	1.21E-03	1.22	4.31E-04	1.25	6.36E-05	1.56E-03
508	P04792	HSPB1	Heat-shock protein beta-1 (Heat shock 27 kDa protein)	Cytoplasm	3. Stress Response and Apoptosis	5.98	22.78	53*	3	-1.55	0.0313	-1.19	0.375	-1.1	0.661	0.105
509	P30084	ECHM	Enoyl-CoA hydratase, mitochondrial	Mitochondria	2.2. Energy Metabolism - Lipid Metabolism/ß-Oxidation	5.88	28.34	9.51	3	1.03	0.891	1.37	2.94E-04	1.36	5.27E-04	3.92E-04
510	P04792	HSPB1	Heat-shock protein beta-1 (Heat shock 27 kDa protein)	Cytoplasm	3. Stress Response and Apoptosis	5.98	22.78	9.5	7	-1.22	0.0504	1.14	0.0216	1.07	0.295	8.96E-04
511	NI	NI	NI - Spot not identified by MS	NI	10. NI - Spot not identified by MS	NI	NI	NI	NI	-1.4	8.73E-05	-1.24	0.0321	-1.66	4.02E-06	8.64E-04
512	NI	NI	NI - Spot not identified by MS	NI	10. NI - Spot not identified by MS	NI	NI	NI	NI	1.02	0.902	1.01	0.925	-1.11	0.365	0.901
513	NI	NI	NI - Spot not identified by MS	NI	10. NI - Spot not identified by MS	NI	NI	NI	NI	1.03	0.825	1.07	0.743	-1.05	0.58	0.562
514	NI	NI	NI - Spot not identified by MS	NI	10. NI - Spot not identified by MS	NI	NI	NI	NI	-1.04	0.723	-1.15	0.225	-1.25	0.0319	0.176
515	NI	NI	NI - Spot not identified by MS	NI	10. NI - Spot not identified by MS	NI	NI	NI	NI	1.04	0.996	1.15	0.184	1.05	0.604	0.528
516	NI	NI	NI - Spot not identified by MS	NI	10. NI - Spot not identified by MS	NI	NI	NI	NI	1.66	4.16E-04	1.49	0.0207	1.57	6.97E-04	1.83E-04

517	NP	NP	NP - Spot not present in the preparative gel	NP	11. NP - Spot not present in the preparative gel	NP	NP	NP	NP	2.02	1.39E-06	1.6	7.48E-04	1.8	2.31E-05	3.04E-07
518	Q9NX63	CHCH3	Coiled-coil-helix-coiled-coil-helix domain-containing protein 3, mitochondrial	Mitochondria	9. Other Functions	undefined	undefined	50*	4	1.03	0.381	1.07	0.142	1.01	0.785	0.39
519	Q9UIJ7	KAD3	GTP:AMP phosphotransferase mitochondrial	Mitochondria	2.6. Other Metabolic Processes	9.16	25.43	11.73	7	-1.21	0.0603	1.03	0.589	-1.05	0.634	7.71E-03
520	P62736	ACTA	Actin, aortic smooth muscle (Alpha-actin-2)	Cytoskeleton	1. Structural and Contractile Proteins	5.24	41.77	9.68	3	-3.37	3.47E-03	-2.5	0.0256	-2.47	0.0188	1.88E-04
521	NP	NP	NP - Spot not present in the preparative gel	NP	11. NP - Spot not present in the preparative gel	NP	NP	NP	NP	1.35	0.058	1.43	0.0148	1.52	3.00E-04	0.0119
522	P12829	MYL4	Myosin light polypeptide 4 (Myosin light chain 1, embryonic muscle/atrial isoform)	Cytoskeleton	1. Structural and Contractile Proteins	4.98	21.43	6.19	5	-2.38	3.89E-03	-1.63	0.0596	-1.32	0.179	3.74E-04
523	O75489	NDUS3	NADH dehydrogenase [ubiquinone] iron-sulfur protein 3, mitochondrial	Mitochondria	2.4. Energy Metabolism - Oxidative Phosphorylation and Electron Transport	5.48	26.41	13.02	10	1.13	0.472	1.07	0.605	1.12	0.405	0.788
524	P12235	ADT1	ADP/ATP translocase 1	Mitochondria	2.6. Other Metabolic Processes	9.78	32.93	46*	3	-1.08	0.616	-1.15	0.305	-1.29	0.0859	0.0182
525	NP	NP	NP - Spot not present in the preparative gel	NP	11. NP - Spot not present in the preparative gel	NP	NP	NP	NP	-1.3	0.0229	-1.01	0.877	-1.2	0.0914	0.0226
526	Q9NX63	CHCH3	Coiled-coil-helix-coiled-coil-helix domain-containing protein 3, mitochondrial	Mitochondria	9. Other Functions	undefined	undefined	58*	4	1.15	2.92E-03	1.19	2.98E-03	1.11	0.041	9.34E-03
527	NI	NI	NI - Spot not identified by MS	NI	10. NI - Spot not identified by MS	NI	NI	NI	NI	1.12	0.158	1.21	2.49E-03	1.21	7.08E-03	3.32E-03
528	NI	NI	NI - Spot not identified by MS	NI	10. NI - Spot not identified by MS	NI	NI	NI	NI	-1.12	0.0636	1.04	0.521	-1.1	0.069	0.0134
529	P60174	TPIS	Triosephosphate isomerase	Cytoplasm	2.1. Energy Metabolism - Glycolysis	6.51	26.54	8.91	6	-1.07	0.0277	-1	0.877	-1.11	2.06E-03	1.23E-03
530	NI	NI	NI - Spot not identified by MS	NI	10. NI - Spot not identified by MS	NI	NI	NI	NI	1.03	0.695	1.08	0.228	1.02	0.671	0.429
531	P60174	TPIS	Triosephosphate isomerase	Cytoplasm	2.1. Energy Metabolism - Glycolysis	6.51	26.54	13.02	10	1.01	0.801	1	0.831	-1.07	0.247	0.537
532	P30041	PRDX6	Peroxiredoxin-6	Cytoplasm	3. Stress Response and Apoptosis	6.02	24.90	61*	3	-1.13	0.107	-1.06	0.395	-1.16	9.49E-03	0.285
533	NP	NP	NP - Spot not present in the preparative gel	NP	11. NP - Spot not present in the preparative gel	NP	NP	NP	NP	1.8	3.61E-07	1.45	1.88E-04	1.76	5.67E-08	2.53E-07
534	NI	NI	NI - Spot not identified by MS	NI	10. NI - Spot not identified by MS	NI	NI	NI	NI	1.12	0.036	1.08	0.134	1.08	0.101	0.209
535	P30041	PRDX6	Peroxiredoxin-6	Cytoplasm	3. Stress Response and Apoptosis	6.02	24.90	35.83	7	-1.2	3.00E-03	-1.05	0.293	-1.22	8.80E-05	2.30E-06
536	P30041	PRDX6	Peroxiredoxin-6	Cytoplasm	3. Stress Response and Apoptosis	6.02	24.90	12.28	6	-1.18	0.0137	-1.11	0.0711	-1.19	2.70E-03	5.14E-03
537	NI	NI	NI - Spot not identified by MS	NI	10. NI - Spot not identified by MS	NI	NI	NI	NI	1.19	5.21E-03	1.11	1.74E-03	1.1	0.0585	0.0258
538	NI	NI	NI - Spot not identified by MS	NI	10. NI - Spot not identified by MS	NI	NI	NI	NI	3.75	6.08E-05	1.87	0.0161	2.5	6.02E-04	2.66E-05
539	O75489	NDUS3	NADH dehydrogenase [ubiquinone] iron-sulfur protein 3, mitochondrial	Mitochondria	2.4. Energy Metabolism - Oxidative Phosphorylation and Electron Transport	5.48	26.41	10.61	5	1.16	4.98E-03	1.21	8.09E-04	1.16	0.0184	6.30E-03
540	P60174	TPIS	Triosephosphate isomerase	Cytoplasm	2.1. Energy Metabolism - Glycolysis	6.51	26.54	63*	4	1.21	0.0278	1.04	0.293	1.12	0.0123	0.0613
541	NP	NP	NP - Spot not present in the preparative gel	NP	11. NP - Spot not present in the preparative gel	NP	NP	NP	NP	1.23	0.0597	1.29	0.0205	1.27	3.77E-03	0.0157
542	P50461	CSRP3	Cysteine and glycine-rich protein 3	Cytoplasm	9. Other Functions	8.89	20.97	57*	4	2.26	8.87E-04	2.09	1.56E-03	2.34	1.08E-03	3.78E-07
543	NP	NP	NP - Spot not present in the preparative gel	NP	11. NP - Spot not present in the preparative gel	NP	NP	NP	NP	1.22	0.0556	1.08	0.49	1.1	0.134	0.0178
544	NI	NI	NI - Spot not identified by MS	NI	10. NI - Spot not identified by MS	NI	NI	NI	NI	-1.01	0.873	-1.08	0.28	1.03	0.497	0.079
545	NI	NI	NI - Spot not identified by MS	NI	10. NI - Spot not identified by MS	NI	NI	NI	NI	1.05	0.469	-1.08	0.344	-1.04	0.648	0.806
546	NP	NP	NP - Spot not present in the preparative gel	NP	11. NP - Spot not present in the preparative gel	NP	NP	NP	NP	-1.37	4.84E-04	-1.22	0.014	-1.27	1.63E-03	1.26E-03
547	P30042	ES1	ES1 protein homolog, mitochondrial	Mitochondria	9. Other Functions	6.63	24.02	69*	4	-1.1	0.412	1.07	0.313	-1.09	0.414	0.034
548	P24539	AT5F1	ATP synthase subunit b, mitochondrial	Mitochondria	2.4. Energy Metabolism - Oxidative Phosphorylation and Electron Transport	9.10	24.63	61*	11	-1.43	1.35E-05	-1.12	0.0556	-1.33	9.06E-05	4.58E-06
549	P28161	GSTM2	Glutathione S-transferase Mu 2	Cytoplasm	2.6. Other Metabolic Processes	6.02	25.61	48*	4	-1.14	0.0494	-1.07	0.195	-1.22	1.92E-04	2.44E-05
550	NI	NI	NI - Spot not identified by MS	NI	10. NI - Spot not identified by MS	NI	NI	NI	NI	-1.14	0.0229	-1.05	0.258	-1.09	0.158	0.12
551	NI	NI	NI - Spot not identified by MS	NI	10. NI - Spot not identified by MS	NI	NI	NI	NI	-1.05	0.534	1.02	0.772	-1.07	0.302	0.727
552	NI	NI	NI - Spot not identified by MS	NI	10. NI - Spot not identified by MS	NI	NI	NI	NI	-1.25	0.0281	1.01	0.824	-1.14	0.081	9.18E-03
553	NP	NP	NP - Spot not present in the preparative gel	NP	11. NP - Spot not present in the preparative gel	NP	NP	NP	NP	-1.32	5.27E-05	-1.15	0.0118	-1.31	3.06E-03	3.26E-04

554	NI	NI	NI - Spot not identified by MS	NI	10. NI - Spot not identified by MS	NI	NI	NI	NI	-3.35	3.43E-03	-2.11	0.0209	-2.5	6.52E-03	1.26E-03
555	Q9ULI4	KI26A	Kinesin-like protein KIF26A	Cytoskeleton	1. Structural and Contractile Proteins	9.12	194.59	72*	10	-1.36	0.19	-1.92	6.47E-03	-1.6	0.0489	0.0193
556	NP	NP	NP - Spot not present in the preparative gel	NP	11. NP - Spot not present in the preparative gel	NP	NP	NP	NP	-1.98	7.39E-03	-1.7	0.043	-1.78	0.0241	8.03E-03
557	NI	NI	NI - Spot not identified by MS	NI	10. NI - Spot not identified by MS	NI	NI	NI	NI	-1.11	0.187	-1.01	0.826	-1.09	0.169	0.197
558	P24539	AT5F1	ATP synthase subunit b, mitochondrial	Mitochondria	2.4. Energy Metabolism - Oxidative Phosphorylation and Electron Transport	9.10	24.63	68*	8	-1.17	0.122	1	0.927	-1.12	0.25	0.0366
559	P02647	APOA1	Apolipoprotein A-I precursor	Secreted	9. Other Functions	5.27	28.08	62*	4	1.01	0.964	-1.02	0.748	-1.07	0.435	0.74
560	P25787	PSA2	Proteasome subunit alpha type-2	Cytoplasm	8. Proteasome-Ubiquitin Process	7.12	25.77	56*	3	1.08	0.448	-1.07	0.64	1.02	0.705	0.38
561	P30043	BLVRB	Flavin reductase	Cytoplasm	2.6. Other Metabolic Processes	7.31	21.99	51*	3	-1.44	1.64E-03	-1.26	0.0341	-1.46	2.74E-03	1.14E-04
562	NI	NI	NI - Spot not identified by MS	NI	10. NI - Spot not identified by MS	NI	NI	NI	NI	1.06	0.571	1.03	0.913	-1.02	0.674	0.968
563	P08590	MYL3	Myosin light polypeptide 3 (Myosin light chain 1, slow-twitch muscle B/ventricular)	Cytoskeleton	1. Structural and Contractile Proteins	5.03	21.80	10.3	4	-1.05	0.578	-1.05	0.597	-1.1	0.326	0.863
564	P08590	MYL3	Myosin light polypeptide 3 (Myosin light chain 1, slow-twitch muscle B/ventricular)	Cytoskeleton	1. Structural and Contractile Proteins	5.03	21.80	13.84	7	-1.13	0.105	-1.02	0.815	-1.15	0.139	0.437
565	P08670	VIME	Vimentin	Cytoskeleton	1. Structural and Contractile Proteins	5.06	53.52	53*	5	-1.29	6.18E-05	-1.04	0.348	-1.28	1.45E-06	5.19E-06
566	P08590	MYL3	Myosin light polypeptide 3 (Myosin light chain 1, slow-twitch muscle B/ventricular isoform)	Cytoskeleton	1. Structural and Contractile Proteins	5.03	21.80	7.41	3	-1.26	6.91E-04	-1.09	0.194	-1.2	1.32E-03	0.031
567	P19404	NDUV2	NADH dehydrogenase [ubiquinone] flavoprotein 2, mitochondrial	Mitochondria	2.4. Energy Metabolism - Oxidative Phosphorylation and Electron Transport	5.71	23.76	48*	3	-1.29	5.05E-05	-1.12	1.24E-03	-1.35	2.04E-09	1.26E-06
568	NI	NI	NI - Spot not identified by MS	NI	10. NI - Spot not identified by MS	NI	NI	NI	NI	-1.11	0.0634	1.04	0.438	-1.14	3.54E-03	0.0314
569	NP	NP	NP - Spot not present in the preparative gel	NP	11. NP - Spot not present in the preparative gel	NP	NP	NP	NP	-1.18	0.332	-1.1	0.641	-1.3	0.0888	0.0718
570	NI	NI	NI - Spot not identified by MS	NI	10. NI - Spot not identified by MS	NI	NI	NI	NI	-1.09	0.156	-1.09	0.0309	-1.15	0.011	0.148
571	NI	NI	NI - Spot not identified by MS	NI	10. NI - Spot not identified by MS	NI	NI	NI	NI	-1.14	0.0187	-1.05	0.31	-1.14	5.95E-03	0.238
572	P24539	AT5F1	ATP synthase subunit b, mitochondrial	Mitochondria	2.4. Energy Metabolism - Oxidative Phosphorylation and Electron Transport	9.10	24.63	64*	7	-1.59	6.44E-04	-1.37	2.76E-03	-1.32	1.48E-03	9.16E-05
573	Q96FI4	NEIL1	Endonuclease VIII-like 1	Nucleus	3. Stress Response and Apoptosis	9.94	43.55	54*	3	-1.43	7.56E-04	-1.16	0.0706	-1.37	8.44E-05	3.50E-04
574	NI	NI	NI - Spot not identified by MS	NI	10. NI - Spot not identified by MS	NI	NI	NI	NI	-1.29	9.05E-03	-1.1	0.229	-1.36	1.70E-03	1.04E-03
575	P09211	GSTP1	Glutathione S-transferase P	Cytoplasm	2.6. Other Metabolic Processes	5.44	23.22	68*	5	-1.02	0.704	-1.13	7.24E-03	-1.15	4.53E-03	5.82E-03
576	NI	NI	NI - Spot not identified by MS	NI	10. NI - Spot not identified by MS	NI	NI	NI	NI	1.3	0.361	1.71	0.078	1.22	0.0324	0.274
577	NI	NI	NI - Spot not identified by MS	NI	10. NI - Spot not identified by MS	NI	NI	NI	NI	1.08	0.256	-1.05	0.462	1.07	0.226	0.0399
578	P08590	MYL3	Myosin light polypeptide 3 (Myosin light chain 1, slow-twitch muscle B/ventricular isoform)	Cytoskeleton	1. Structural and Contractile Proteins	5.03	21.80	11.7	7	-1.19	9.11E-04	-1.09	0.0782	-1.24	1.37E-03	9.74E-03
579	P04179	SODM	Superoxide dismutase [Mn], mitochondrial	Mitochondria	3. Stress Response and Apoptosis	6.86	22.20	7.83	6	1.03	0.819	-1.12	0.182	1.05	0.405	0.139
580	P00568	KAD1	Adenylate kinase isoenzyme 1	Cytoplasm	2.6. Other Metabolic Processes	8.73	21.63	6.75	4	-1.47	2.23E-03	-1.32	0.0106	-1.26	6.72E-03	3.91E-03
581	NI	NI	NI - Spot not identified by MS	NI	10. NI - Spot not identified by MS	NI	NI	NI	NI	-1.25	0.0273	-1.08	0.428	-1.18	0.0675	4.79E-04
582	NI	NI	NI - Spot not identified by MS	NI	10. NI - Spot not identified by MS	NI	NI	NI	NI	1.07	0.525	-1.06	0.64	1.11	0.234	0.206
583	P30086	PEBP1	Phosphatidylethanolamine-binding protein 1	Cytoplasm	9. Other Functions	7.43	20.93	9.18	5	-1.17	6.03E-03	-1.06	0.193	-1.19	3.35E-04	1.54E-03
584	NI	NI	NI - Spot not identified by MS	NI	10. NI - Spot not identified by MS	NI	NI	NI	NI	-1.23	0.0129	-1.12	0.141	-1.2	0.0383	0.0102
585	NI	NI	NI - Spot not identified by MS	NI	10. NI - Spot not identified by MS	NI	NI	NI	NI	1.71	3.31E-03	1.17	0.0261	1.36	2.15E-05	7.76E-03
586	P32119	PRDX2	Peroxiredoxin-2	Cytoplasm	3. Stress Response and Apoptosis	5.67	21.76	8.52	6	-1.16	1.93E-04	-1.1	0.0208	-1.17	8.42E-03	2.18E-03
587	P02511	CRYAB	Alpha crystallin B chain	Cytoplasm	3. Stress Response and Apoptosis	6.76	20.16	8.13	3	-1.29	0.0318	-1.04	0.564	-1.05	0.529	0.332
588	O75947	ATP5H	ATP synthase D chain, mitochondrial	Mitochondria	2.4. Energy Metabolism - Oxidative Phosphorylation and Electron Transport	5.22	18.36	9.1	7	-1.26	8.51E-05	-1.09	0.0387	-1.23	1.46E-03	1.86E-05
589	NI	NI	NI - Spot not identified by MS	NI	10. NI - Spot not identified by MS	NI	NI	NI	NI	-1.04	0.667	-1.01	0.981	1.04	0.573	0.955
590	NP	NP	NP - Spot not present in the preparative gel	NP	11. NP - Spot not present in the preparative gel	NP	NP	NP	NP	-1.09	0.117	-1.06	0.341	-1.1	0.0301	0.465

591	NI	NI	NI - Spot not identified by MS	NI	10. NI - Spot not identified by MS	NI	NI	NI	NI	-1.09	0.0528	1.03	0.589	-1.04	0.306	0.35
592	NI	NI	NI - Spot not identified by MS	NI	10. NI - Spot not identified by MS	NI	NI	NI	NI	-1.09	0.0828	1.01	0.852	-1.07	0.188	0.572
593	NP	NP	NP - Spot not present in the preparative gel	NP	11. NP - Spot not present in the preparative gel	NP	NP	NP	NP	-1.29	9.09E-05	-1.12	0.0399	-1.23	2.45E-03	6.37E-04
594	NI	NI	NI - Spot not identified by MS	NI	10. NI - Spot not identified by MS	NI	NI	NI	NI	-1.29	0.0213	-1.14	0.242	-1.15	0.03	0.459
595	P51970	NDUA8	NADH dehydrogenase [ubiquinone] 1 alpha subcomplex subunit 8	Mitochondria	2.4. Energy Metabolism - Oxidative Phosphorylation and Electron Transport	7.93	19.97	50*	3	-1.01	0.975	-1.1	0.461	-1.16	0.265	0.655
596	P63316	TNNC1	Troponin C, slow skeletal and cardiac muscles	Cytoskeleton	1. Structural and Contractile Proteins	4.04	18.40	91*	6	-1.01	0.9	-1.06	0.662	1	0.982	0.986
597	NP	NP	NP - Spot not present in the preparative gel	NP	11. NP - Spot not present in the preparative gel	NP	NP	NP	NP	-1.34	0.108	-1.59	0.0291	-1.85	2.09E-03	0.0536
598	Q9UBY9	HSPB7	Heat shock protein beta-7	Cytoplasm	3. Stress Response and Apoptosis	6.04	18.61	50*	3	1.5	6.63E-04	1.4	0.0462	1.58	0.023	3.57E-03
599	Q9Y281	COF2	Cofilin-2 (Cofilin, muscle isoform).	Cytoskeleton	1. Structural and Contractile Proteins	7.88	18.61	45.49	7	-1.12	0.212	-1.04	0.547	-1.28	0.0113	0.088
600	NP	NP	NP - Spot not present in the preparative gel	NP	11. NP - Spot not present in the preparative gel	NP	NP	NP	NP	-1.19	0.501	-1.38	0.152	-1.34	0.183	0.311
601	NP	NP	NP - Spot not present in the preparative gel	NP	11. NP - Spot not present in the preparative gel	NP	NP	NP	NP	1	0.72	-1.08	0.822	-1.07	0.928	0.721
602	NI	NI	NI - Spot not identified by MS	NI	10. NI - Spot not identified by MS	NI	NI	NI	NI	-1.25	0.0134	-1.02	0.906	-1.14	0.107	7.25E-03
603	NI	NI	NI - Spot not identified by MS	NI	10. NI - Spot not identified by MS	NI	NI	NI	NI	1.41	0.0481	-1.16	0.154	1.08	0.577	0.0358
604	NI	NI	NI - Spot not identified by MS	NI	10. NI - Spot not identified by MS	NI	NI	NI	NI	1.22	0.0309	1.02	0.91	1.27	0.0221	0.0159
605	NI	NI	NI - Spot not identified by MS	NI	10. NI - Spot not identified by MS	NI	NI	NI	NI	-1.35	1.80E-03	-1.02	0.791	-1.28	2.44E-03	7.85E-04
606	P02144	MYG	Myoglobin	Cytoplasm	9. Other Functions	7.29	17.05	42*	2	-1.48	0.0239	-1	0.737	-1.1	0.381	0.118
607	P05413	FABPH	Fatty acid-binding protein, heart	Cytoplasm	7. Transport and Transmembrane Transport	6.34	14.73	8.45	4	-1.01	0.956	-1.34	0.152	-1	0.915	0.543
608	NI	NI	NI - Spot not identified by MS	NI	10. NI - Spot not identified by MS	NI	NI	NI	NI	-1.54	0.031	1.11	0.861	-1.28	0.135	0.549
609	NP	NP	NP - Spot not present in the preparative gel	NP	11. NP - Spot not present in the preparative gel	NP	NP	NP	NP	-1.58	0.0134	-1.16	0.48	-1.39	0.0662	0.0291
610	P02144	MYG	Myoglobin	Cytoplasm	9. Other Functions	7.29	17.05	62*	3	-1.17	0.0309	1.09	0.386	-1.23	8.30E-05	1.18E-04
611	NP	NP	NP - Spot not present in the preparative gel	NP	11. NP - Spot not present in the preparative gel	NP	NP	NP	NP	1.48	4.50E-03	1.14	0.414	1.34	0.0283	1.05E-03
612	P02144	MYG	Myoglobin	Cytoplasm	9. Other Functions	7.29	17.05	58*	3	-1.44	0.0506	-1.03	0.888	-1.35	0.0747	0.101
613	P02144	MYG	Myoglobin	Cytoplasm	9. Other Functions	7.29	17.05	43*	2	-1.86	8.93E-03	-1.3	0.21	-1.7	0.0176	0.0413
614	P00568	KAD1	Adenylate kinase isoenzyme 1	Cytoplasm	2.6. Other Metabolic Processes	8.73	21.63	10.94	6	1.35	0.0719	1.07	0.471	1.09	0.425	0.365
615	NP	NP	NP - Spot not present in the preparative gel	NP	11. NP - Spot not present in the preparative gel	NP	NP	NP	NP	1.04	0.771	1.07	0.582	-1.16	0.138	0.304
616	NI	NI	NI - Spot not identified by MS	NI	10. NI - Spot not identified by MS	NI	NI	NI	NI	-4.41	4.68E-03	-2.98	0.0241	-3.11	0.0156	2.58E-04
617	NI	NI	NI - Spot not identified by MS	NI	10. NI - Spot not identified by MS	NI	NI	NI	NI	-1.23	0.0382	1.14	0.0571	-1.22	7.98E-03	1.47E-03
618	NI	NI	NI - Spot not identified by MS	NI	10. NI - Spot not identified by MS	NI	NI	NI	NI	-1.15	0.0323	-1.09	0.228	-1.18	0.0659	0.077
619	NI	NI	NI - Spot not identified by MS	NI	10. NI - Spot not identified by MS	NI	NI	NI	NI	2.47	1.93E-04	2.18	8.00E-04	1.81	9.28E-03	4.46E-05
620	Q9P0J0	NDUAD	NADH dehydrogenase [ubiquinone] 1 alpha subcomplex subunit 13	Mitochondria	2.4. Energy Metabolism - Oxidative Phosphorylation and Electron Transport	8.24	16.57	7.6	5	-1.2	0.0232	-1.04	0.586	-1.25	2.22E-03	5.94E-03
621	P31930	QCR1	Cytochrome b-c1 complex subunit 1, mitochondrial	Mitochondria	2.4. Energy Metabolism - Oxidative Phosphorylation and Electron Transport	5.43	49.13	10.8	7	-1.23	6.73E-04	-1.05	0.29	-1.17	2.26E-03	1.84E-04
622	P02647	APOA1	Apolipoprotein A-I precursor	Secreted	9. Other Functions	5.27	28.08	11.1	8	1.91	3.91E-03	1.57	0.0298	1.78	0.0106	3.61E-03
623	NI	NI	NI - Spot not identified by MS	NI	10. NI - Spot not identified by MS	NI	NI	NI	NI	-1.18	0.0152	-1.16	0.0467	-1.06	0.281	0.038
624	P07195	LDHB	L-lactate dehydrogenase B chain	Cytoplasm	2.1. Energy Metabolism - Glycolysis	5.72	36.51	62*	6	-1.18	0.023	1.04	0.536	-1.14	0.0389	6.71E-05
625	NI	NI	NI - Spot not identified by MS	NI	10. NI - Spot not identified by MS	NI	NI	NI	NI	-1.05	0.348	-1.06	0.327	-1.1	0.0422	0.426
626	P02511	CRYAB	Alpha crystallin B chain	Cytoplasm	3. Stress Response and Apoptosis	6.76	20.16	7.83	3	-1.1	0.111	-1.02	0.674	1.03	0.66	0.525
627	P07900	HS90A	Heat shock protein HSP 90-alpha	Cytoplasm	3. Stress Response and Apoptosis	4.94	84.53	11.82	15	1.56	8.26E-04	1.27	0.0189	1.55	4.84E-05	6.85E-07

628	P68032	ACTC	Actin, alpha cardiac muscle 1 (Alpha-cardiac actin)	Cytoskeleton	1. Structural and Contractile Proteins	5.23	41.78	10.55	7	-1.11	0.0108	-1.06	0.192	-1.1	0.157	0.0901
629	P42126	D3D2	3,2-trans-enoyl-CoA isomerase, mitochondrial	Mitochondria	2.2. Energy Metabolism - Lipid Metabolism/ß-Oxidation	6.00	28.74	48*	3	-1.35	9.17E-03	-1.24	5.28E-03	-1.03	0.584	1.68E-03
630	P22695	QCR2	Cytochrome b-c1 complex subunit 2, mitochondrial	Mitochondria	2.4. Energy Metabolism - Oxidative Phosphorylation and Electron Transport	7.74	46.78	12.89	8	-1.36	1.77E-03	-1.13	0.0357	-1.32	1.39E-05	4.74E-09
631	NI	NI	NI - Spot not identified by MS	NI	10. NI - Spot not identified by MS	NI	NI	NI	NI	1.16	6.24E-03	1.01	0.979	1.26	9.09E-05	2.04E-06
632	P25705	ATPA	ATP synthase subunit alpha, mitochondrial	Mitochondria	2.4. Energy Metabolism - Oxidative Phosphorylation and Electron Transport	8.28	55.21	14.88	13	1.07	0.0641	1.09	0.0205	1.02	0.611	6.14E-03
633	Q16851	UGPA	UTP--glucose-1-phosphate uridylyltransferase	Cytoplasm	2.6. Other Metabolic Processes	8.15	56.81	88*	9	1.56	3.38E-06	1.25	2.85E-04	1.36	5.98E-04	2.85E-06
634	P35609	ACTN2	Alpha-actinin-2 (Alpha actinin skeletal muscle isoform 2)	Cytoskeleton	1. Structural and Contractile Proteins	5.31	103.85	9.8	12	1.28	0.0301	1.09	0.385	1.23	0.137	0.0651
635	P21912	DHSB	Succinate dehydrogenase [ubiquinone] iron-sulfur subunit, mitochondrial	Mitochondria	2.3. Energy Metabolism - Tricarboxylic Acid Cycle	8.78	28.81	24.89	7	-1.55	2.05E-05	-1.15	0.0477	-1.53	1.32E-05	8.61E-08
636	NI	NI	NI - Spot not identified by MS	NI	10. NI - Spot not identified by MS	NI	NI	NI	NI	-2.24	4.12E-06	-1.48	0.0168	-2.37	1.24E-06	3.55E-07
637	P09622	DLDH	Dihydrolipoyl dehydrogenase, mitochondrial precursor	Mitochondria	2.4. Energy Metabolism - Oxidative Phosphorylation and Electron Transport	6.50	50.17	9.31	6	1.15	0.0314	1.13	0.085	1.17	0.0564	0.0205
638	P10916	MLRV	Myosin regulatory light chain 2, ventricular/cardiac muscle isoform	Cytoskeleton	1. Structural and Contractile Proteins	4.92	18.66	65*	5	-1.36	0.0151	-1.03	0.691	-1.46	3.63E-03	0.0942
639	P10916	MLRV	Myosin regulatory light chain 2, ventricular/cardiac muscle isoform	Cytoskeleton	1. Structural and Contractile Proteins	4.92	18.66	65*	5	-1.33	0.0173	-1.02	0.72	-1.45	9.37E-04	0.0996
640	P10916	MLRV	Myosin regulatory light chain 2, ventricular/cardiac muscle isoform	Cytoskeleton	1. Structural and Contractile Proteins	4.92	18.66	66*	5	-1.28	2.47E-03	-1.06	0.451	-1.35	8.25E-04	4.28E-03
641	P10916	MLRV	Myosin regulatory light chain 2, ventricular/cardiac muscle isoform	Cytoskeleton	1. Structural and Contractile Proteins	4.92	18.66	129*	9	-1.1	0.26	-1.14	0.238	-1.06	0.402	0.742
642	P55084	ECHB	Trifunctional enzyme subunit beta, mitochondrial	Mitochondria	2.2. Energy Metabolism - Lipid Metabolism/ß-Oxidation	9.24	47.48	7.89	5	1.06	0.619	1.42	0.0464	1.21	0.0875	0.0393
643	P17540	KCRS	Creatine kinase, sarcomeric mitochondrial	Mitochondria	2.5. Energy Metabolism - Creatine Kinase System (Energy Transduction)	7.28	43.34	13.37	13	-1.25	2.64E-03	-1.08	0.391	-1.13	0.0508	0.0412
644	P02144	MYG	Myoglobin	Cytoplasm	9. Other Functions	7.29	17.05	8.38	6	-1.19	0.0536	1.04	0.819	-1.31	1.49E-04	3.27E-03
645	P17661	DESM	Desmin	Cytoplasm	1. Structural and Contractile Proteins	5.21	53.40	9.49	14	1.3	0.0515	1.18	0.203	1.94	1.09E-05	2.62E-07
646	NP	NP	NP - Spot not present in the preparative gel	NP	11. NP - Spot not present in the preparative gel	NP	NP	NP	NP	1.6	4.42E-03	1.34	0.0523	2.26	9.44E-06	1.52E-07
647	P25705	ATPA	ATP synthase subunit alpha, mitochondrial	Mitochondria	2.4. Energy Metabolism - Oxidative Phosphorylation and Electron Transport	8.28	55.21	13.35	15	1.71	8.80E-05	1.19	0.0249	1.22	0.0184	2.09E-05
648	P25705	ATPA	ATP synthase subunit alpha, mitochondrial	Mitochondria	2.4. Energy Metabolism - Oxidative Phosphorylation and Electron Transport	8.28	55.21	15.22	14	-1.09	0.118	-1.01	0.828	-1.11	0.0575	5.99E-03
649	P14618	KPYM	Pyruvate kinase isozymes M1/M2	Cytoplasm	2.1. Energy Metabolism - Glycolysis	7.95	57.81	68*	5	1.34	2.55E-03	1.05	0.331	1.2	5.02E-03	3.68E-03
650	P11021	GRP78	78 kDa glucose-regulated protein (Heat shock 70 kDa protein 5)	Endoplasmic reticulum	3. Stress Response and Apoptosis	5.01	70.48	43.5	12	1.51	6.80E-04	1.06	0.719	1.56	7.52E-03	3.46E-04
651	P25705	ATPA	ATP synthase subunit alpha, mitochondrial	Mitochondria	2.4. Energy Metabolism - Oxidative Phosphorylation and Electron Transport	8.28	55.21	76*	7	1.61	2.07E-05	1.29	1.82E-03	1.44	2.49E-04	5.01E-06
652	P01024	CO3	Complement C3 precursor	Secreted	4. Immune Response	6.00	184.95	12.31	17	1.21	0.0289	1.21	0.158	1.52	5.37E-04	0.0186
653	O14763	TR10B	Tumor necrosis factor receptor superfamily member 10B	Membrane	3. Stress Response and Apoptosis	4.97	42.23	51*	4	1.33	8.86E-06	1.01	0.969	1.08	0.0287	1.28E-05
654	NP	NP	NP - Spot not present in the preparative gel	NP	11. NP - Spot not present in the preparative gel	NP	NP	NP	NP	1.45	3.22E-03	1.2	0.239	1.46	1.24E-03	0.0368
655	P49588	SYAC	Alanyl-tRNA synthetase, cytoplasmic	Cytoplasm	6. Transcription and Translation Processes	5.34	106.81	58*	8	1.68	1.02E-03	1.26	0.0818	1.61	1.22E-05	4.18E-03
656	Q9UHP3	UBP25	Ubiquitin carboxyl-terminal hydrolase 25	Cytoplasm	2.6. Other Metabolic Processes	5.22	122.22	53*	8	-1.03	0.771	-1.06	0.461	-1.11	0.149	0.121
657	NP	NP	NP - Spot not present in the preparative gel	NP	11. NP - Spot not present in the preparative gel	NP	NP	NP	NP	1.79	4.26E-04	1.09	0.364	1.62	1.40E-04	2.30E-07
658	P02768	ALBU	Serum albumin precursor	Secreted	9. Other Functions	5.67	66.47	9.64	8	2.38	5.65E-04	1.81	0.0107	1.91	1.49E-03	3.85E-04
659	P55809	SCOT1	Succinyl-CoA:3-ketoacid-coenzyme A transferase 1, mitochondrial	Mitochondria	2.6. Other Metabolic Processes	6.00	52.09	6.87	4	1.31	0.0724	1.08	0.449	1.35	7.18E-03	2.37E-03
660	P11310	ACADM	Medium-chain specific acyl-CoA dehydrogenase, mitochondrial	Mitochondria	2.2. Energy Metabolism - Lipid Metabolism/ß-Oxidation	7.02	43.64	9.86	8	-1.34	4.26E-04	-1.07	0.358	-1.22	5.96E-03	3.30E-06
661	P36957	ODO2	2-oxoglutarate dehydrogenase E2 component, mitochondrial	Mitochondria	2.3. Energy Metabolism - Tricarboxylic Acid Cycle	5.90	41.39	9.26	4	-1.09	0.096	-1.03	0.579	-1.04	0.408	0.0407
662	P09972	ALDOC	Fructose-bisphosphate aldolase C (Brain-type aldolase)	Cytoplasm	2.1. Energy Metabolism - Glycolysis	6.46	39.32	7.53	4	-1.22	1.73E-03	-1.04	0.365	-1.24	1.20E-03	3.06E-05
663	P00505	AATM	Aspartate aminotransferase, mitochondrial	Mitochondria	2.6. Other Metabolic Processes	8.98	44.74	8.15	5	-1.06	0.492	1.03	0.716	-1.02	0.88	0.548
664	P07195	LDHB	L-lactate dehydrogenase B chain	Cytoplasm	2.1. Energy Metabolism - Glycolysis	5.72	36.51	7.68	3	-1.2	0.0267	1.08	0.384	-1.13	0.085	2.76E-04

665	P40925	MDHC	Malate dehydrogenase, cytoplasmic	Cytoplasm	2.6. Other Metabolic Processes	6.89	36.29	50*	5	-1.14	0.0116	-1.03	0.456	-1.15	0.0294	3.18E-03
666	Q16698	DECR	2,4-dienoyl-CoA reductase, mitochondrial	Mitochondria	2.2. Energy Metabolism - Lipid Metabolism/ß-Oxidation	8.79	32.15	33.81	4	-1.28	0.0169	1.09	0.341	-1.23	0.0174	2.00E-04
667	P02743	SAMP	Serum amyloid P-component precursor	Secreted	3. Stress Response and Apoptosis	6.12	23.26	7.57	4	1.33	0.079	1.24	0.0223	1.53	3.32E-03	0.0104
668	P04792	HSPB1	Heat-shock protein beta-1 (Heat shock 27 kDa protein)	Cytoplasm	3. Stress Response and Apoptosis	5.98	22.78	11.02	7	-1.13	9.39E-03	-1.04	0.376	1.03	0.713	0.0923
669	NI	NI	NI - Spot not identified by MS	NI	10. NI - Spot not identified by MS	NI	NI	NI	NI	-1.04	0.672	1.09	0.285	1.01	0.817	0.372
670	P60709	ACTB	Actin, cytoplasmic 1 (Beta-actin).	Cytoskeleton	1. Structural and Contractile Proteins	5.29	41.74	8.9	6	1.16	0.0431	-1.09	0.0545	1	0.986	1.45E-03
671	NP	NP	NP - Spot not present in the preparative gel	NP	11. NP - Spot not present in the preparative gel	NP	NP	NP	NP	-1.15	8.17E-03	-1.03	0.548	-1.13	0.0122	0.0272
672	P02511	CRYAB	Alpha crystallin B chain	Cytoplasm	3. Stress Response and Apoptosis	6.76	20.16	7.58	4	-1.19	0.0965	-1.19	0.0523	-1.39	1.05E-04	0.203
673	Q99497	PARK7	Protein DJ-1	Nucleus	5. Cell Signaling	6.33	19.89	61*	4	1.01	0.773	-1.14	0.264	-1.13	0.188	0.09
674	P30048	PRDX3	Peroxiredoxin-3	Mitochondria	3. Stress Response and Apoptosis	5.77	21.47	7.14	4	-1.15	3.15E-03	-1	0.885	-1.13	4.16E-03	0.0246
675	P02144	MYG	Myoglobin	Cytoplasm	9. Other Functions	7.29	17.05	58*	3	-1.61	7.28E-04	-1.15	0.256	-1.39	0.0105	2.41E-03
676	P27144	KAD4	Adenylate kinase isoenzyme 4, mitochondrial	Mitochondria	2.6. Other Metabolic Processes	8.47	25.27	7.55	4	-1.05	0.546	1.04	0.557	-1.07	0.433	0.186
677	NI	NI	NI - Spot not identified by MS	NI	10. NI - Spot not identified by MS	NI	NI	NI	NI	-1.33	7.95E-04	1	0.974	-1.22	8.58E-03	6.29E-04
678	P17661	DESM	Desmin	Cytoplasm	1. Structural and Contractile Proteins	5.21	53.40	64*	11	1.83	3.94E-03	1.59	0.0246	2.65	1.04E-05	4.45E-07
679	P02144	MYG	Myoglobin	Cytoplasm	9. Other Functions	7.29	17.05	9.96	6	-1.22	0.0374	1.07	0.534	-1.29	4.56E-04	1.32E-03
680	P36871	PGM1	Phosphoglucomutase-1	Cytoplasm	2.6. Other Metabolic Processes	6.32	61.32	7.2	4	1.02	0.76	-1.18	0.171	-1.13	0.247	0.117
681	P68032	ACTC	Actin, alpha cardiac muscle 1 (Alpha-cardiac actin)	Cytoskeleton	1. Structural and Contractile Proteins	5.23	41.78	10.6	7	-1.15	7.83E-04	-1.09	6.61E-03	-1.13	0.024	0.0172
682	P09493	TPM1	Tropomyosin-1 alpha chain	Cytoskeleton	1. Structural and Contractile Proteins	4.69	32.71	8.77	10	-1.21	2.00E-03	-1.06	0.275	-1.19	0.029	0.0123
683	P06732	KCRM	Creatine kinase M-type	Cytoplasm	2.5. Energy Metabolism - Creatine Kinase System (Energy Transduction)	6.77	43.10	11.93	8	-1.82	2.86E-05	-1.3	5.14E-03	-1.93	7.81E-09	1.89E-11

Index: Number of the “spot” in the master gel (the gel with higher number of spots and used for reference for the matches).
Uniprot: Accession number of the protein in the UniProt (Universal Protein Resource) database.
Symbol: Name of protein entry in databases.
Protein Description: Protein name according to UniProt database.
Function: The metabolic process in which the protein is involved. Name: Protein name according to UniProt database.
MS Identification - Score: Identifications were performed in two ways: For values that do not have an asterisk, identification was performed using the in-house developed software “Poseidon”. The “score” value represents the probability that the identification was performed randomly. Eg.: the value of 6.33 means that the probability of such identification being incorrect is $10^{-6.33}$. For values with an asterisk, identification was performed using the Mascot program (www.matrixscience.com). In this, the “score” value represents the absolute probability that the identification was performed randomly. The Mascot program considers events that have a randomness frequency lower than 5% as significant. In the searches performed in the experiments, “scores” greater than 53 were considered significant (p<0.05).
MS Identification - Peptides: number of peptides whose mass/charge ratio was identified by mass spectrometry and which correlated with theoretical values in the databases for the specific protein.
NP: “Spot” not present in the preparative gel.
NI: “Spot” not identified by mass spectrometry.
